# Anti–miR-93-5p therapy prolongs sepsis survival by restoring the peripheral immune response

**DOI:** 10.1172/JCI158348

**Published:** 2023-07-17

**Authors:** Mihnea P. Dragomir, Enrique Fuentes-Mattei, Melanie Winkle, Keishi Okubo, Recep Bayraktar, Erik Knutsen, Aiham Qdaisat, Meng Chen, Yongfeng Li, Masayoshi Shimizu, Lan Pang, Kevin Liu, Xiuping Liu, Simone Anfossi, Huanyu Zhang, Ines Koch, Anh M. Tran, Swati Mohapatra, Anh Ton, Mecit Kaplan, Matthew W. Anderson, Spencer J. Rothfuss, Robert Silasi, Ravi S. Keshari, Manuela Ferracin, Cristina Ivan, Cristian Rodriguez-Aguayo, Gabriel Lopez-Berestein, Constantin Georgescu, Pinaki P. Banerjee, Rafet Basar, Ziyi Li, David Horst, Catalin Vasilescu, Maria Teresa S. Bertilaccio, Katayoun Rezvani, Florea Lupu, Sai-Ching Yeung, George A. Calin

**Affiliations:** 1 Department of Experimental Therapeutics, The University of Texas MD Anderson Cancer Center (MDACC), Houston, Texas, USA.; 2 Department of Surgery, Fundeni Clinical Hospital, Carol Davila University of Medicine and Pharmacy, Bucharest, Romania.; 3 Institute of Pathology, Charité-Universitätsmedizin Berlin, Corporate Member of Freie Universität Berlin, Humboldt-Universität zu Berlin, Berlin, Germany.; 4 German Cancer Consortium (DKTK), Partner Site Berlin, and German Cancer Research Center (DKFZ), Heidelberg, Germany.; 5 Berlin Institute of Health (BIH), Berlin, Germany.; 6 Department of Translational Molecular Pathology, The University of Texas MDACC, Houston, Texas, USA.; 7 Department of Medical Biology, Faculty of Health Sciences, UiT – The Arctic University of Norway, Tromsø, Norway.; 8 Department of Emergency Medicine, The University of Texas MDACC, Houston, Texas, USA.; 9 Department of Breast Surgery, Institute of Cancer and Basic Medicine (ICBM), Chinese Academy of Science; Cancer Hospital of the University of Chinese Academy of Sciences, Zhejiang Cancer Hospital, Hangzhou, Zhejiang, China.; 10 Department of Radiation Physics, The University of Texas MDACC, Houston, Texas, USA.; 11 Department of Pediatric Surgery, The Affiliated Hospital of Qingdao University, Qingdao, Shandong, China.; 12 Baylor College of Medicine, Graduate School of Biomedical Sciences, Houston, Texas, USA.; 13 Department of Stem Cell Transplantation and Cellular Therapy, The University of Texas MDACC, Houston, Texas, USA.; 14 Cardiovascular Biology Research Program, Oklahoma Medical Research Foundation, Oklahoma City, Oklahoma, USA.; 15 Department of Medical and Surgical Sciences (DIMEC), University of Bologna, Bologna, Italy.; 16 Center for RNA Interference and Non-coding RNAs, The University of Texas MDACC, Houston, Texas, USA.; 17 Genes and Human Disease Research Program, Oklahoma Medical Research Foundation, Oklahoma City, Oklahoma.; 18 Department of Biostatistics, The University of Texas MDACC, Houston, Texas, USA.

**Keywords:** Immunology, Infectious disease, Adaptive immunity, Innate immunity, Noncoding RNAs

## Abstract

Sepsis remains a leading cause of death for humans and currently has no pathogenesis-specific therapy. Hampered progress is partly due to a lack of insight into deep mechanistic processes. In the past decade, deciphering the functions of small noncoding miRNAs in sepsis pathogenesis became a dynamic research topic. To screen for new miRNA targets for sepsis therapeutics, we used samples for miRNA array analysis of PBMCs from patients with sepsis and control individuals, blood samples from 2 cohorts of patients with sepsis, and multiple animal models: mouse cecum ligation puncture–induced (CLP-induced) sepsis, mouse viral miRNA challenge, and baboon Gram^+^ and Gram^–^ sepsis models. miR-93-5p met the criteria for a therapeutic target, as it was overexpressed in baboons that died early after induction of sepsis, was downregulated in patients who survived after sepsis, and correlated with negative clinical prognosticators for sepsis. Therapeutically, inhibition of miR-93-5p prolonged the overall survival of mice with CLP-induced sepsis, with a stronger effect in older mice. Mechanistically, anti–miR-93-5p therapy reduced inflammatory monocytes and increased circulating effector memory T cells, especially the CD4^+^ subset. AGO2 IP in miR-93–KO T cells identified important regulatory receptors, such as *CD28*, as direct miR-93-5p target genes. In conclusion, miR-93-5p is a potential therapeutic target in sepsis through the regulation of both innate and adaptive immunity, with possibly a greater benefit for elderly patients than for young patients.

## Graphical abstract

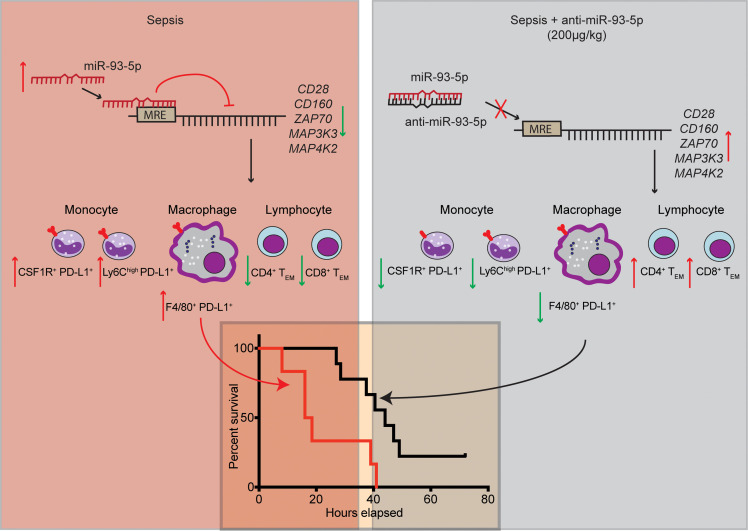

## Introduction

Sepsis is a life-threatening organ dysfunction that is mainly caused by an inadequate or dysregulated host response to infection (1). Worldwide, each year there are approximately 49 million patients diagnosed with sepsis and 11 million sepsis-related deaths (2). In the United States alone, it is estimated that 1.7 million adults are diagnosed with sepsis each year, and approximately 30%–50% of hospitalized patients die from sepsis (3, 4), making it one of the deadliest human diseases. Despite efforts to reduce its incidence and mortality, the numbers are rising every year (5), burdening society and the health care system (2).

Most patients affected by sepsis are elderly, have multiple comorbidities, and have a functionally impaired immune system (6, 7). Additionally, the physiopathology of sepsis differs according to age: young patients develop a preeminent, systemic proinflammatory response, whereas elderly patients tend to have an uncontrollable antiinflammatory response (8, 9). Correspondingly, the cause of death and the ideal therapy differ between these patient groups, and, unfortunately, clinical trials only exceptionally consider these differences (10).

Clinical trials for sepsis using therapies that target the pathogenic pathways are notoriously likely to fail (11). Recent data showed that sepsis clinical trials would have obtained better results if a stratification according to clinical phenotypes of sepsis had been done before therapy (10). Therefore, a proper selection of patients according to clinical phenotypes may increase the likelihood of success. Also, the in vivo models of sepsis used to develop many of the therapies that ultimately failed also neglected the critical biological differences of age and typically used young animals in preclinical tests for new therapies (12).

Sepsis immunobiology has yet to be completely deciphered: distinct phases of hyperinflammation and immunoparalysis have been identified that can occur sequentially or simultaneously (13). Death can occur at both stages: hyperinflammation can induce early death due to uncontrolled hyperinflammatory reactions or late death due to inflammation-induced organ injury or multiorgan failure, while immunoparalysis can induce late death due to immunosuppression-associated recurrent infections or suprainfections (14). Indeed, long-term follow-up of patients with sepsis revealed that both patients with persistently high levels of proinflammatory biomarkers and patients with high levels of immunosuppressive biomarkers had shorter long-term survival compared with those with normal levels after sepsis recovery (15). The state of hyperinflammation is mainly driven by innate immune cells (monocytes, macrophages, and neutrophils), the complement cascade, and the coagulation system (13), while immunosuppression and immunoparalysis largely affect the adaptive immune system, including CD4^+^ and CD8^+^ T lymphocytes displaying an increased expression of inhibitory receptors (16, 17). Some exceptions exist; for example, it is known that peritoneal neutrophils produce high amounts of IL-10 in the early phase of cecal ligation and puncture–induced (CLP-induced) sepsis (18).

miRNAs are small noncoding RNAs that can bind mRNA “targets” by imperfect sequence complementarity and cause the degradation of these mRNAs or inhibit their translation (19). miRNAs were initially associated with sepsis over a decade ago (20), and multiple other studies have further confirmed miRNA dysregulation (21). In addition, we showed that both endogenous miRNAs, and also viral miRNAs that can bind TLR8, are altered in sepsis (22, 23), that miRNA networks in sepsis are reorganized (24), and that the same sepsis-related miRNAs are dysregulated after splenectomy (25), a condition that increases the risk of sepsis (26).

We used 2 original strategies to develop a potentially new miRNA therapy for sepsis. First, we screened multiple animal models and clinical sample sets to select dysregulated, immune-related miRNAs as potential therapeutic targets for sepsis, and second, we tested the miRNA-based therapy in mice of different ages and of both sexes to assess their preclinical efficacy. We started the in vivo screen using the CLP-induced sepsis mouse model (also used for the therapeutic studies) and the Kaposi’s sarcoma–associated herpesvirus kshv-miR-K12-12*–induced inflammation mouse model, which recapitulates activation of TLRs relevant to sepsis (22). Next, we used samples from 2 lethal sepsis baboon models to confirm that the selected miRNA target is dysregulated in higher primates and in both Gram^+^ and Gram^–^ sepsis models. Finally, we studied the molecular mechanisms regulated by the selected miRNAs using in vitro CRISPR/Cas9-KO models.

## Results

### miR-93-5p is a potential therapeutic target in sepsis.

**Figure 1:**
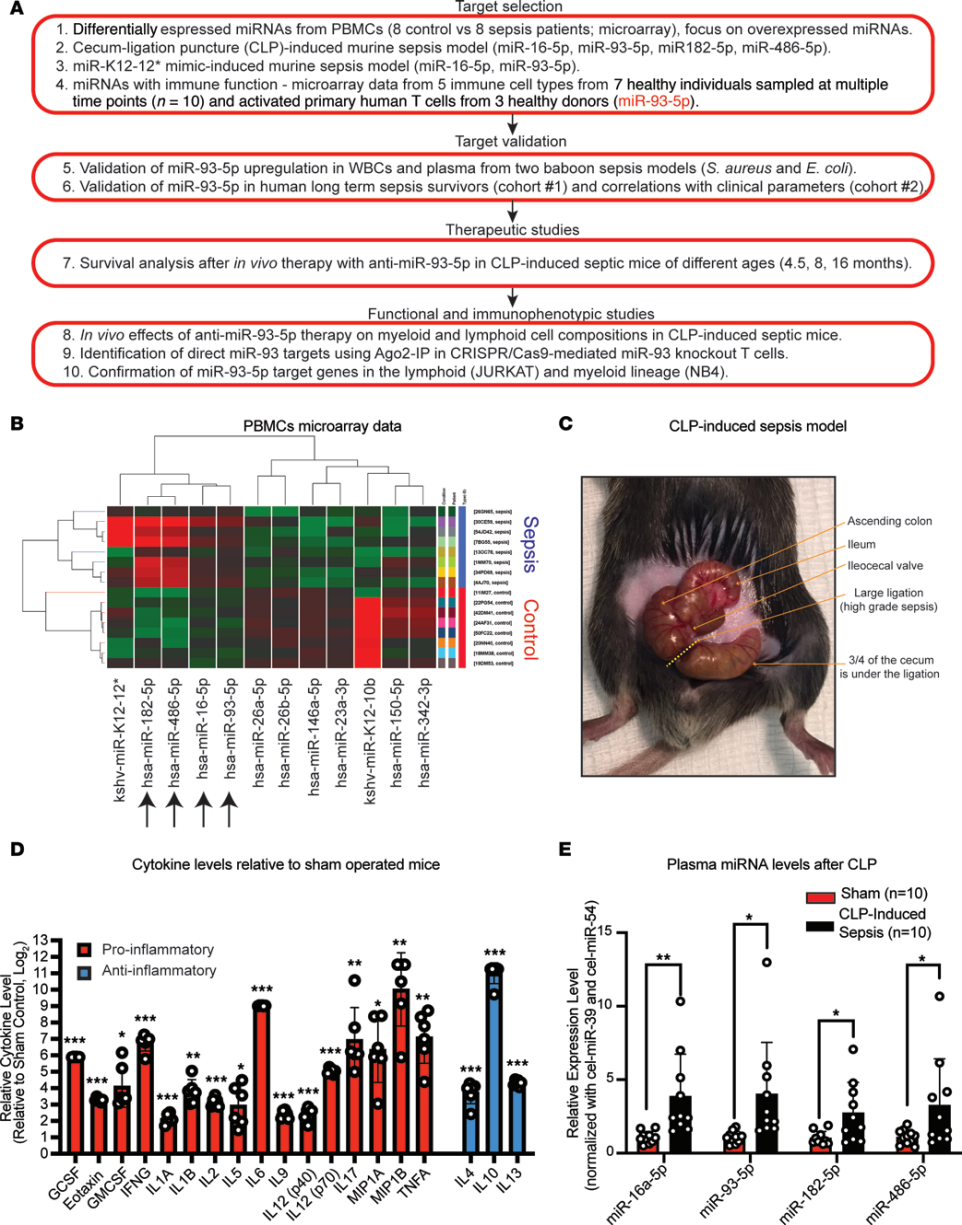
miR-93-5p is a potential therapeutic target in sepsis. (**A**) Schematic workflow of the selection, confirmation, and therapeutic and comprehensive functional characterization of an upregulated miRNA target in sepsis. (**B**) Heatmap displaying the miRNAs most differentially expressed in PBMCs from patients with sepsis (*n* = 8) compared with PBMCs from healthy controls (*n* = 8). Black arrows represent human miRNAs upregulated in PBMCs from patients with sepsis. (**C**) Representative image of the CLP-induced sepsis mouse model (8-month-old mouse). (**D**) Bar graphs of log_2_-transformed mean plasma levels of measured pro- and antiinflammatory cytokines in CLP-induced septic mice (*n* = 10) relative to sham-operated mice (*n* = 10). All mice were 8 months old. Proinflammatory cytokines: granulocyte-CSF (G-CSF), eotaxin, GM-CSF, IFN-γ (IFNG), IL-1α (IL1A), IL-1β (IL1B), IL-2 (IL2), IL-5 (IL5), IL-6 (IL6), IL-9 (IL9), IL-12 (p40) [IL12(p40)], IL-12 (p70) [IL12(p70)], IL-17 (IL17), macrophage inflammatory protein 1α (MIP1A), macrophage inflammatory protein 1β (MIP1B), TNF-α (TNFA); antiinflammatory cytokines: IL-4 (IL4), IL-10 (IL10), and IL-13 (IL13). Data represent the mean ± SD. (**E**) Plasma levels of selected miRNAs in sham-operated mice (*n* = 10) compared with CLP-induced septic mice (*n* = 10). These mice were 8 months old. The relative expression level was normalized to cel-miR-39-3p and cel-miR-54-3p. Data are presented as the mean ± SD. *P < 0.05, **P < 0.01, and ***P < 0.001, by 2-tailed Student’s *t* test.

The workflow to select the best putative candidate miRNA for the potential development of an anti-miRNA therapy is presented in Figure 1A. We started by using previously reported genome-wide miRNA expression data (20, 22), in which we compared PBMCs from patients with sepsis versus those from healthy individuals. Principal component analysis (PCA) showed a separate grouping of control and sepsis samples in the 3 main principal component dimensions (Supplemental Figure 1; supplemental material available online with this article; https://doi.org/10.1172/JCI158348DS1). We found 5 miRNAs that were markedly upregulated (miR-16-5p, miR-93-5p, miR-182-5p, miR-486-5p, and kshv-miR-K12-12*) and 7 miRNAs that were substantially downregulated (miR-26a-5p, miR-26b-5p, miR-146a-5p, miR-23a-3p, miR-150-5p, miR-342-3p, and kshv-miR-K12-10b) in PBMCs from patients with sepsis compared with control PBMCs (Figure 1B). As miRNA restoration therapy has been shown to induce multiple side effects, including a life-threatening systemic inflammatory response syndrome (27), we decided to focus on miRNAs that are overexpressed in sepsis. Moreover, viral miRNAs were not further investigated as therapeutic targets, since humans, baboons, and mice are not affected by the same viruses, thus imposing limitations for further in vivo studies.

We used the murine CLP-induced sepsis model, which is considered to more closely resemble human sepsis among the available mouse models (28) (Figure 1C). We observed the induction of 16 proinflammatory and 3 antiinflammatory cytokines in the CLP-induced sepsis group compared with the sham surgery–treated group (8-month-old mice, *n* = 10 mice per group) (Figure 1D), proving the occurrence of sepsis after CLP but not after sham surgery. All 4 aforementioned nonviral miRNAs that were upregulated in patients with sepsis were significantly overexpressed in plasma from mice in the CLP group compared with plasma from the sham-operated group (Figure 1E).

In a previous study, we showed that viral kshv-miR-K12-12* was upregulated in patients with sepsis and acted as a direct agonist of TLR8, contributing to the pathophysiology of sepsis (22). To assess whether any of the candidate miRNAs are regulated by the pathways activated by viral miRNAs, we injected 8-week-old C57BL/6J mice i.p. with kshv-miR-K12-12* or the scrambled negative control (a miRNA mimic molecule with no effect on miRNA functions) (Supplemental Figure 2A). Circulating viral kshv-miR-K12-12* was significantly increased 24 hours after i.p. injection, confirming successful delivery. This caused an induction of miR-93-5p and miR-16-5p in mice injected with kshv-miR-K12-12*, whereas miR-182-5p showed no significant changes (Supplemental Figure 2B).

To explore the involvement of these miRNAs in sepsis-related immunobiology, we used a publicly available data set of global miRNA expression by microarray in CD14^+^ monocytes, CD15^+^ granulocytes, CD56^+^ NK cells, CD3^+^ T cells, and CD19^+^ B cells from 7 healthy donors sampled at multiple time points (*n* = 10) (29) (Supplemental Figure 2C). miR-182 showed the lowest expression, followed by miR-486. miR-93 showed high and consistent expression across all samples, with the highest levels in monocytes and granulocytes and moderate yet substantial expression in B, T, and NK cells. Very high levels of miR-16 were present in all cell types from all samples. miR-16 is thus already expressed at exceptionally high levels in healthy immune cells; therefore, it is questionable that further upregulation of this miRNA in sepsis will have important functional consequences. To gain further insight into the dynamic regulation of these miRNAs in primary human T cells, we activated pan–T cells isolated from 3 healthy donors using stimulation with CD3/CD28-coated beads and IL-2 for 96 hours. As expected, T cell activation induced a significant, on average 150-fold, induction of IFN-γ (*IFNG*) transcript levels (*P* < 0.01) (Supplemental Figure 2D). miR-486-5p was expressed at low levels and showed a minor decrease in levels upon T cell activation (*P* = 0.122), whereas miR-16-5p was highly expressed and showed no difference between resting and activated T cells (*P* = 0.321) (Supplemental Figure 2, E and F). Finally, miR-93-5p was expressed at low levels in resting T cells and showed a mean 5-fold induction upon T cell activation (*P* < 0.05) (Supplemental Figure 2G), suggesting a significant involvement of miR-93-5p in T cell dynamics. On the basis of all the above sets of data, we considered miR-93-5p to be the most promising candidate for further therapeutic investigation.

### miR-93-5p is dysregulated in 2 baboon sepsis models.

**Figure 2:**
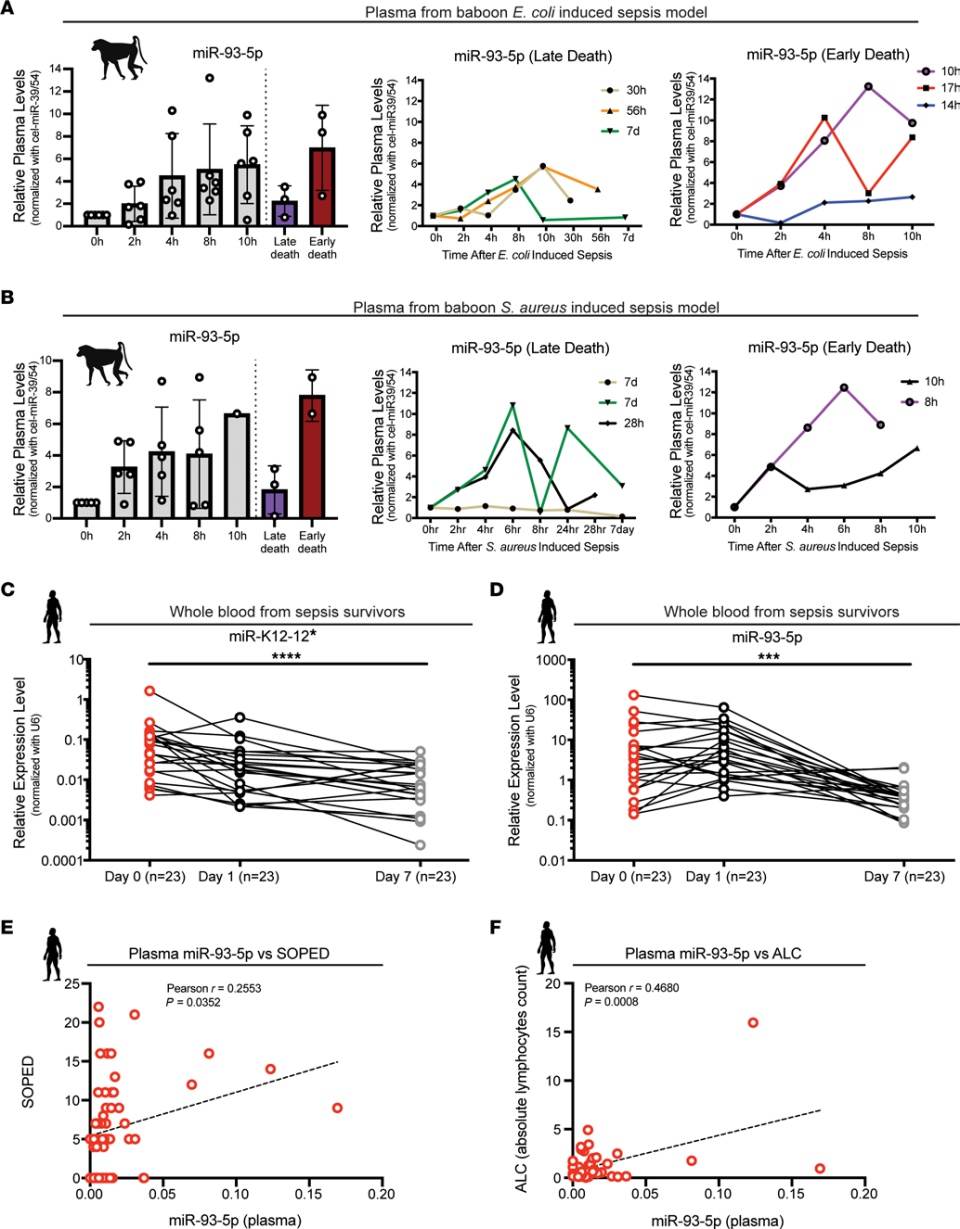
miR-93-5p expression in 2 baboon models of sepsis and in long-term sepsis survivors. (**A**) Left panel: Plasma levels of miR-93-5p at different time points in an *E. coli* (Gram–) baboon sepsis model (*n* = 6). Purple bar represents the expression of miR-93-5p before death in baboons that died late after inoculation; red bar represents the expression of miR-93-5p before death in baboons that died early after inoculation. Middle panel: miR-93-5p dynamics in *E. coli*–inoculated baboons that died late (*n* = 3). Right panel: miR-93-5p dynamics in *E. coli*–inoculated baboons that died early (*n* = 3). (B) Left panel: Plasma levels of miR-93-5p at different time points in an *S. aureus* (Gram+) baboon sepsis model (*n* = 5). Purple bar represents the expression of miR-93-5p before death in baboons that died late after inoculation; red bar represents the expression of miR-93-5p before death in baboons that died early after inoculation. Middle panel: miR-93-5p dynamics in *S. aureus*–inoculated baboons that died late (*n* = 3). Right panel: miR-93-5p dynamics in baboons *S. aureus*–inoculated baboons that died early (*n* = 2). Expression of (C) miR-K12-12* and (D) miR-93-5p in whole blood from long-term survivors of sepsis (*n* = 23) at 3 different time points: day 0 = shortly after sepsis diagnosis, day 1 = 1 day after diagnosis, and day 7 = 7 days after sepsis diagnosis. The relative expression level was normalized to U6. Data are presented as the mean ± SD. ***P < 0.001 and ****P < 0.0001, by Friedman’s test. (E) Correlation between miR-93-5p levels in plasma and SOPED score (*n* = 59). (F) Correlation between miR-93-5p levels in plasma and the ALC (*n* = 48). Data were evaluated by Pearson’s correlation test (**E** and **F**).

To confirm miR-93-5p as a potential therapeutic target, we used 2 baboon models of lethal sepsis: (a) live *Escherichia coli* (30) and (b) heat-inactivated *Staphylococcus aureus* (31), both i.v. infused. In the plasma of the baboon *E*. *coli* sepsis model (Gram^–^), the expression of miR-93-5p progressively increased from time-point 0 (just before bacterial induction), reaching maximum expression 8 to 10 hours after sepsis was induced (Figure 2A). Of the 6 analyzed baboons, 3 died within 24 hours (10 hours, 17 hours, and 14 hours, respectively), which was classified as an early death, and the other 3 survived for more than 24 hours (30 hours, 56 hours, and 7 days, which was the survival endpoint, respectively), which was classified as a late death. We observed that early death was associated with higher miR-93-5p levels when compared with levels in the late death group (Figure 2A). We also observed the same phenomenon in WBCs from animals in the *E*. *coli*–induced sepsis model: 1 of 3 animals, a baboon that survived the challenge, showed an early increase in miR-93-5p levels after induction of sepsis and a subsequent decrease as it survived (Supplemental Figure 3A).

In the plasma of the *S*. *aureus* (Gram^+^) sepsis model, the expression of miR-93-5p progressively increased from time-point 0 to a maximum expression level at 10 hours after i.v. injection of *S*. *aureus*. Two of the 5 baboons analyzed died within 24 hours (8 hours, 10 hours — early deaths), while the other 3 survived for more than 24 hours (1 for 28 hours and 2 for 7 days – late deaths). Thus, we found that early death was associated with high miR-93-5p levels also in Gram^+^ sepsis (Figure 2B). In WBCs from baboons in the *S*. *aureus*–induced sepsis model, we detected lower miR-93-5p expression after sepsis induction compared with the increase in miR-93-5p expression detected in WBCs from baboons in the *E*. *coli*–induced sepsis model, but the expression dynamics were similar (Supplemental Figure 3B). We therefore hypothesize that miR-93-5p is a potential therapeutic target and prognostic biomarker in both Gram^–^ and Gram^+^ sepsis.

miR-93-5p is located within intron 12 of the coding gene minichromosome maintenance complex component 7 (*MCM7*) at chromosome 7q22.1; hence, we wanted to check whether *MCM7* had a similar change in expression after induction of sepsis. In both baboon sepsis models, we observed a gradual decrease of *MCM7* in WBCs after induction of sepsis (Supplemental Figure 3, C and D), which did not match the observed dynamics of miR-93-5p. Therefore, we concluded that different mechanisms regulate the expression of the miRNA and the host coding gene.

### Low levels of miR-93-5p are associated with long-term survival of patients.

Twenty-three patients with sepsis from MDACC who survived more than 7 days in the intensive care unit (ICU) underwent serial sample collections at 3 different time points (cohort 1): at the diagnosis of sepsis (day 0), 1 day after the diagnosis (day 1), and 7 days after the diagnosis (day 7) (Supplemental Table 1). As death from sepsis usually occurs within 3 days of the diagnosis (8), this cohort represents a subgroup of patients who survived after sepsis, selected from an original group of more than 100 patients with sepsis. This cohort is composed solely of patients with cancer, and sepsis in these patients is characterized mainly by a dysregulated immune response (15, 32). Additionally, the median age of this group is 60 years, supporting once more the immunosuppressive sepsis model (8, 9). In whole blood from the long-term survivors, after the diagnosis of sepsis, kshv-miR-K12-12* expression gradually dropped (*P* < 0.0001) (Figure 2C), and miR-93-5p was characterized by an abrupt and significant decrease in expression (*P* < 0.001) (Figure 2D), especially between day 1 and day 7. kshv-miR-K12-12* expression dropped by 70.72% between day 0 and day 1 and by 66.85% between day 1 and day 7, while miR-93-5p dropped by 18.76% between day 0 and day 1 and by 95.33% between day 1 and day 7.

We further analyzed a larger and clinically well-annotated cohort of cancer patients with sepsis (cohort 2, *n* = 63, Supplemental Table 2), from whom we had collected both plasma and PBMCs. We observed that miR-93-5p levels in plasma at the time of sepsis diagnosis significantly and positively correlated with the Septic Oncologic Patients in the Emergency Department (SOPED) score (*r* = 0.26, *P* < 0.05) (Figure 2E). SOPED is a clinical tool that can predict the risk of 7-day mortality in cancer patients with sepsis (33). miR-93-5p levels in plasma at the time of sepsis diagnosis were also significantly and positively correlated with the absolute lymphocyte count (ALC) (*r* = 0.47, *P* < 0.001) (Figure 2F). In PBMCs, miR-93-5p levels showed no correlation with the SOPED or ALC (Supplemental Figure 4, A and B). Moreover, the Acute Physiology and Chronic Health Evaluation II (APACHE II) score was near-significantly correlated with miR-93-5p levels in PBMCs (*r* = 0.23, *P* = 0.08) (Supplemental Figure 4C), but not with miR-93-5p levels in plasma (Supplemental Figure 5A). Conversely, the absolute neutrophil count (ANC) was near-significantly correlated with miR-93-5p levels in plasma (*r* = 0.27, *P* = 0.07) (Supplemental Figure 5B), but not with miR-93-5p levels in PBMCs (Supplemental Figure 4D). We did not observe any significant correlation for PBMC or plasma miR-93-5p levels with the Sepsis-related Organ Failure Assessment (SOFA) score, C-reactive protein (CRP) levels, the Mortality in Emergency Department Sepsis (MEDS) score, or the Charlson Comorbidity Index (CCI) (Supplemental Figure 4, E–H, and Supplemental Figure 5, C–F). Therefore, miR-93-5p levels in different blood compartments correlated in a different way with some clinical prognosticators for sepsis.

miR-93 is part of a cluster of miRNA genes, including miR-25 and miR-106b, located within intron 12 of the coding gene *MCM7*. Therefore, we checked for any coregulation of the miRNA cluster transcripts in sepsis. The levels of miR-25-3p and miR-106b-5p in plasma and PBMCs showed a high correlation with miR-93-5p levels, suggesting their transcriptional coregulation (Supplemental Figure 6, A and B). On the other hand, we did not observe any correlation between levels of *MCM7* and miR-93-5p (neither in PBMCs nor plasma) (Supplemental Figure 6, A and B). Additionally, for the significant parameters (SOPED and ALC) and in the same biofluid (plasma), we also performed correlation analyses for miR-25-3p and miR-106b-5p. We found that miR-25-3p levels significantly correlated with the ALC (*r* = 0.66, *P* < 0.0001), but not with the SOPED score (*r* = 0.24, *P* = 0.07) (Supplemental Figure 6C). In contrast, miR-106b-5p levels significantly correlated with the SOPED (*r* = 0.27, *P* < 0.05), but not with the ALC (*r* = –0.07, *P* = 0.65) (Supplemental Figure 6D).

In summary, sepsis survival is associated with a gradual drop in the expression of kshv-miR-K12-12* and an abrupt drop in miR-93-5p expression, and the levels of miR-93-5p at sepsis diagnosis correlated with 7-day mortality rates. These data indicated that miR-93-5p is a suitable candidate for anti-miRNA therapy.

### Anti–miR-93-5p therapy improves survival in old mice with sepsis.

**Figure 3:**
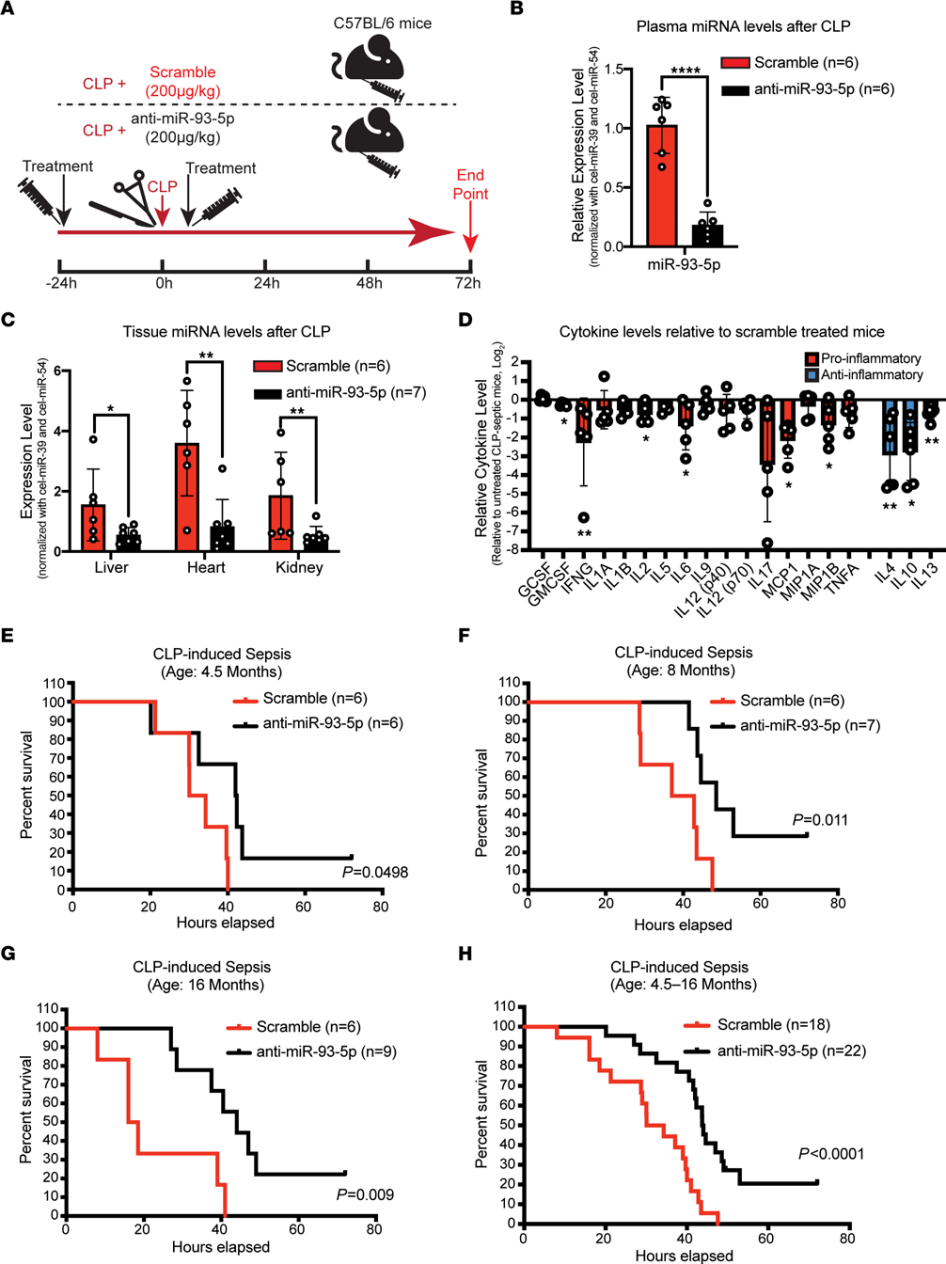
Effect of anti–miR-93-5p therapy in the CLP-induced sepsis model. (**A**) Twenty-four hours before CLP-induced sepsis, male and female C57BL/6 mice of different ages were treated i.p. with scrambled miRNA or anti–miR-93-5p. The treatment was repeated 2 hours after the induction of sepsis. Mice were followed up for 72 hours after CLP. (**B**) Plasma levels of miR-93-5p in septic mice treated with scrambled miRNA (*n* = 6) compared with septic mice treated with anti–miR-93-5p (*n* = 6). (**C**) Tissue levels of miR-93-5p in mice with sepsis treated with scrambled miRNA (*n* = 6) compared with septic mice treated with anti–miR-93-5p (*n* = 7) in organs frequently affected by sepsis: liver, heart, and kidney. (**D**) Pro- and antiinflammatory cytokine levels in anti–miR-93-5p–treated mice relative to levels in mice treated with scrambled miRNA. Data are presented as the mean ± SD. *P < 0.05, **P < 0.01, and ****P < 0.0001, by 2-tailed Student’s *t* test (**B**–**D**). (**E**) Kaplan-Meier survival analysis for 4.5-month-old CLP-induced septic mice treated with scrambled miRNA (*n* = 6) versus CLP-induced septic mice treated with anti–miR-93-5p (*n* = 6). (**F**) Kaplan-Meier survival analysis for 8-month-old CLP-induced septic mice treated with scrambled miRNA (*n* = 6) versus CLP-induced septic mice treated with anti–miR-93-5p (*n* = 7). (**G**) Kaplan-Meier survival analysis for 16-month-old CLP-induced septic mice treated with scrambled miRNA (*n* = 6) versus CLP-induced septic mice treated with anti–miR-93-5p (*n* = 9). (**H**) Kaplan-Meier survival analysis for all CLP-induced septic mice treated with scrambled miRNA together (*n* = 18) versus all CLP-induced septic mice treated with anti–miR-93-5p together (*n* = 22).

Next, we investigated the therapeutic potential of targeting miR-93-5p in the CLP-induced sepsis model (Supplemental Figure 7) in mice of different ages. Age and sex were investigated as biological variables that would influence sepsis outcome. Age- and sex-matched mice were treated i.p. with 200 μg/kg anti–miR-93-5p or scrambled control miRNA, 24 hours before and 2 hours after the induction of sepsis (Figure 3A). We first assessed the levels of miR-93-5p in plasma and the major organs frequently affected by sepsis. At the time of death, we observed a significant decrease in miR-93-5p levels in all samples from anti–miR-93-5p–treated compared with scramble-treated mice, with the largest reduction seen in plasma (fold change decrease of 0.82, *P* < 0.0001) (Figure 3B), followed by kidney and heart (fold change decrease of 0.71 and 0.77 respectively, *P* < 0.01) and liver (fold change decrease of 0.65, *P* < 0.05) (Figure 3C). Next, we compared the plasma levels of pro- and antiinflammatory cytokines between anti–miR-93-5p and scrambled control–treated mice. The levels of 6 proinflammatory cytokines (granulocyte-macrophage CSF [GM-CSF], IFN-γ, IL-2, IL-6, MCP1, and MIP1β) and of 3 antiinflammatory cytokines (IL-4, IL-10, and IL-13) were significantly lower in the plasma of mice treated with anti–miR-93-5p than in those treated with the scrambled control miRNA (Figure 3D). These data suggested that both the systemic proinflammatory and the compensatory antiinflammatory responses were diminished by anti–miR-93-5p therapy.

To examine the effect of age on sepsis outcome, we compared young (4.5-month-old) mice, middle-aged (8-month-old) mice, and old (16-month-old) mice for the CLP sepsis model. We observed an age-dependent survival benefit for mice treated with anti–miR-93-5p therapy. In young mice, the median survival was 32.18 hours for the scrambled control miRNA–treated group versus 42.18 hours for the anti–miR-93-5p–treated group (*P* = 0.0498) (Figure 3E), whereas in middle-aged mice, the median survival difference was more significant, increasing from 39.95 hours for the scrambled control miRNA–treated group to 48.57 hours for the anti–miR-93-5p–treated group (*P* = 0.011) (Figure 3F). The most robust survival benefit was seen in old mice, with a median survival of 17.25 hours in the scrambled control miRNA–treated group versus 44 hours in the anti–miR-93-5p–treated group (*P* = 0.009) (Figure 3G). A pooled analysis of mice of all ages demonstrated that anti–miR-93-5p pretreatment and continued treatment with anti–miR-93-5p improved the overall survival for mice with CLP-induced sepsis (median survival: 32.175 hours for the scrambled control group vs. 43.875 hours for anti–miR-93-5p group, *P* < 0.0001) (Figure 3H). In a multivariate Cox regression analysis of survival that included age and anti–miR-93 therapy, the therapy alone was the only factor significantly associated with prolonged survival (*P* = 0.0002) (Table 1).

To investigate whether there was an age-dependent difference in the levels of miR-93-5p expression in mice with CLP-induced sepsis, we induced sepsis in young (4.5-month-old) mice (*n* = 3) and very old (30-month-old) mice (*n* = 6). None of the mice was treated with anti–miR-93-5p during the experiment. We observed no significant difference in miR-93-5p expression levels among these age groups (*P* = 0.814) (Supplemental Figure 8).

We next analyzed the effects of anti–miR-93-5p therapy at the tissue level. We analyzed tissue damage in lungs, hearts, livers, spleens, and kidneys from the 16-month-old mice with CLP-induced sepsis in the aforementioned anti–miR-93-5p survival experiment. We observed no histopathological difference in the lungs and hearts between the scrambled control miRNA–treated and anti–miR-93-5p–treated mice (Supplemental Figure 9, A and B). In contrast, we identified significant differences in the abdominal organs: when compared with anti–miR-93-5p–treated mice, the scrambled control miRNA–treated mice showed higher liver injury scores, characterized especially by necrosis and immune infiltrates (*P* < 0.05, Supplemental Figure 9C), higher spleen injury scores (*P* < 0.05, Supplemental Figure 9D), and higher kidney injury scores (*P* < 0.05, Supplemental Figure 9E). Morphologically apoptotic cells were observed in lungs and spleens, but no difference in their numbers between the 2 groups was detected (Supplemental Figure 9, F and G). Interestingly, in the lungs and spleens of scrambled control miRNA–treated mice compared with anti–miR-93-5p–treated mice, we observed a near-significant (*P* = 0.08) (Supplemental Figure 9H) accumulation of periodic acid–Schiff (PAS) stain–positive cells with macrophage morphology in the lung interstitium and a significantly higher number of PAS^+^ cells in the splenic white pulp (*P* < 0.05) (Supplemental Figure 9I).

In conclusion, anti–miR-93-5p combined prophylaxis and therapy significantly prolonged the overall survival of mice with CLP-induced sepsis, especially if the mice were old, and this survival benefit was not linked to differences in the basal level of miR-93-5p expression when sepsis occurred, but was most probably due to the effect of anti–miR-93-5p in restoring the immune balance.

### Anti–miR-93-5p therapy affects the peripheral immune response.

**Figure 4:**
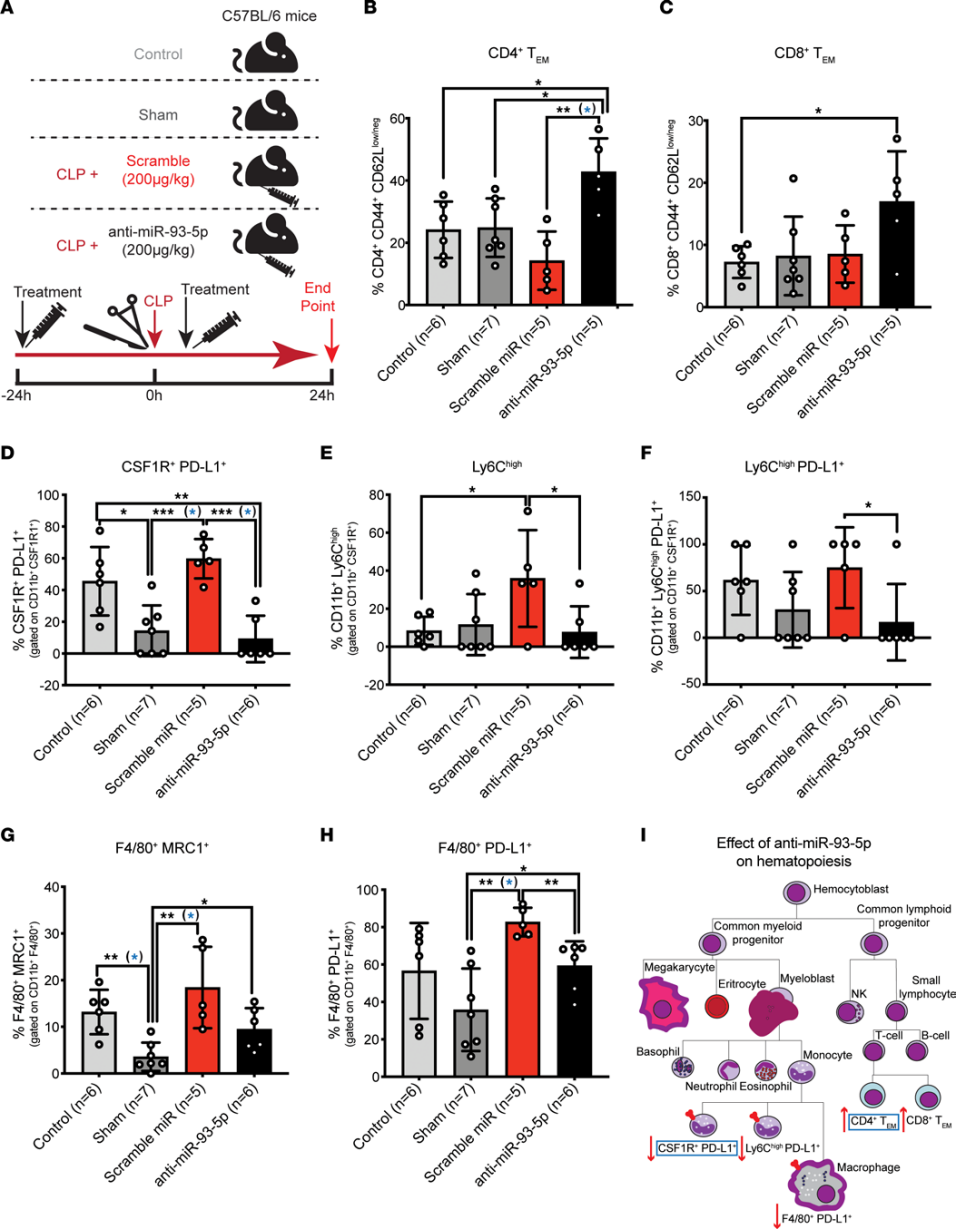
Effect of anti–miR-93-5p therapy on the lymphoid and myeloid lineages. (**A**) Flow cytometric characterization of circulating lymphoid and myeloid cells in 4 different mouse groups: control (no intervention), sham-operated, CLP-induced sepsis with scrambled miRNA treatment, and CLP-induced sepsis with anti–miR-93-5p treatment. Treatment was administrated 24 hours before and 2 hours after sepsis induction. Mice were sacrificed 24 hours after surgery (sham) or CLP-induced sepsis. Control mice were sacrificed together with mice in the other 3 groups. (**B**) Percentage of CD4^+^CD44^+^CD62L^lo/neg^ Tem cells (CD4^+^ Tem) in control mice, sham-operated mice, CLP-induced septic mice treated with scrambled miRNA, and CLP-induced septic mice treated with anti–miR-93-5p mice. (**C**) Percentage of CD8^+^ Tem cells (CD8^+^ Tem) in all 4 groups. (**D**) Percentage of CSF1R^+^PD-L1^+^ cells in the entire pool of CD11b^+^CSF1R^+^ monocytes in control mice, sham-operated mice, CLP-induced septic mice treated with scrambled miRNA, and CLP-induced septic mice treated with anti–miR-93-5p. (**E**) Percentage of LyC^hi^ cells in the entire pool of CD11b^+^CSF1R^+^ monocytes and of (**F**) LyC^hi^PD-L1^+^ monocytes in control mice, sham-operated mice, CLP-induced septic mice treated with scrambled miRNA, and CLP-induced septic mice treated with anti–miR-93-5p. (**G**) Percentage of F4/80^+^MRC1^+^ macrophages and of (**H**) F4/80^+^PD-L1^+^ macrophages in all 4 experimental mouse groups. (**I**) Schematic representation of the effect of anti–miR-93-5p therapy on hematopoiesis during sepsis. Blue rectangles mark the cell subtypes that were significantly differentially expressed in the anti–miR-93-5p–treated group versus the scrambled miRNA–treated group after adjustment for multiple testing using the FDR. Data are presented as the mean ± SD. *P < 0.05, **P < 0.01, and ***P < 0.001, by 2-tailed Student’s *t* test (**B**–**H**). *P* values that are significant after adjustment for multiple testing using the FDR are highlighted in blue.

The effect of anti–miRNA therapy on the immunobiology of the host has, to our knowledge, never been reported. Therefore, we characterized the circulating lymphoid and myeloid cells in sex-matched, mature 8-month-old mice from 4 different groups of mice: control mice (no intervention or treatment), sham-operated mice (surgery without sepsis induction), scrambled control miRNA–treated mice, and anti–miR-93-5p–treated mice (mice of both CLP-induced sepsis models were treated with scrambled control miRNA or anti–miR-93-5p, 24 hours before and 2 hours after CLP) (Figure 4A). This design helped us investigate the inflammation-specific (differentially expressed in sham-operated, scrambled control–treated, and anti–miR-93-5p–treated mice vs. control mice); the sepsis-specific (differentially expressed in scrambled control– and anti–miR-93-5p–treated mice vs. control and sham-operated mice); and the anti–miR-93-5p therapy–specific (differentially expressed in anti–miR-93-5p–treated mice vs. mice in the other 3 groups) changes in immune cells of myeloid and lymphoid lineage.

With regard to CD19^+^ B cells, we observed, as expected (34), a significant sepsis-specific decrease in scrambled miRNA and anti–miR-93-5p treatment groups compared with the control and sham groups (anti–miR-93-5p vs. sham: *P* < 0.001; scrambled control vs. sham: *P* < 0.01; anti–miR-93-5p vs. control: *P* < 0.001; scrambled control vs. control: *P* < 0.01) (Supplemental Figure 10A). The expression of the checkpoint molecule programmed death ligand 1 (PD-L1) on B cells resembled an inflammation-specific response with significant downregulation in the sham (*P* < 0.05) and anti–miR-93-5p groups (*P* < 0.05) compared with the control group, and a smaller, nonsignificant reduction in the scrambled control group (Supplemental Figure 10B).

The frequency of CD4^+^ T cells strongly increased in a sepsis-specific manner, with higher levels in both scrambled control miRNA– and anti–miR-93-5p–treated groups compared with the sham-operated and control groups (anti–miR-93-5p vs. sham: *P* < 0.0001, scrambled control vs. sham: *P* < 0.0001; anti–miR-93-5p vs. control: *P* < 0.05, scrambled control vs. control: *P* < 0.01) (Supplemental Figure 10C). Within the CD4^+^ T cell pool, we further noted a sepsis-specific decrease in CD4^+^ central memory T (Tcm) cells (anti–miR-93-5p vs. sham: *P* < 0.001, scrambled control vs. sham: *P* < 0.01; anti–miR-93-5p vs. control: *P* < 0.01, scrambled control vs. control: *P* < 0.01) (Supplemental Figure 10D). Reductions in CD4^+^ Tcm cells in spleens from the CLP-induced sepsis mouse model have been reported previously (35). Conversely, we observed a strong anti–miR-93-5p therapy–specific increase in CD4^+^ effector memory T (Tem) cells compared with control, sham-operated, and scrambled control miRNA–treated mice (*P* < 0.05, *P* < 0.05, and *P* < 0.01, respectively) (Figure 4B). Consistent with the sepsis- and therapy-specific increases observed in the whole CD4^+^ pool and the CD4^+^ Tem subset, the percentage of CD4^+^ naive cells was significantly reduced in scrambled control miRNA–treated mice and especially in the anti–miR-93-5p–treated mice compared with the sham-operated and control mice (anti–miR-93-5p vs. sham: *P* < 0.05, scrambled control vs. sham: *P* < 0.01; anti–miR-93-5p vs. control: *P* < 0.05) (Supplemental Figure 10E). We observed no differences in CD4^+^CD25^+^ Treg percentages (Supplemental Figure 10F).

The frequency of CD8^+^ T cells was not modified among the groups, including expression of the programmed cell death protein 1 (PD-1) molecule (Supplemental Figure 10, G and H). Consistent with CD4^+^ Tcm cell dynamics, we observed a sepsis-specific decrease in CD8^+^ Tcm cells (scrambled control vs. sham: *P* < 0.05; anti–miR-93-5p vs. control: *P* < 0.05, scrambled control vs. control: *P* < 0.01) (Supplemental Figure 10I). Furthermore, CD8^+^ Tem cells were significantly increased in the anti–miR-93-5p–treated group compared with the control group (*P* < 0.05), but when compared with the sham-operated and scrambled control miRNA–treated groups, the expression appeared to be higher, but not statistically significant (Figure 4C). We detected no differences in PD-1 expression on CD8^+^ Tcm and Tem cell subsets among the groups (Supplemental Figure 10, J and K), and CD8^+^ naive cells were slightly reduced in a sepsis-specific manner (Supplemental Figure 10L). Thus, our results suggest that anti–miR-93-5p therapy regulated T cell dynamics and caused specific expansion of effector cell subsets, which may partially explain the prolonged survival of the anti–miR-93-5p–treated mice.

Regarding innate immune cells, the frequency of CD11b^+^CSF1R^+^ monocytes was constant among the groups (Supplemental Figure 11A), whereas we observed a relevant decrease in CSF1R^+^PD-L1^+^ monocytes in the anti–miR-93-5p–treated and sham-operated mice compared with the sepsis mouse model mice treated with scrambled control miRNA (*P* < 0.001 for both comparisons) and control mice (*P* < 0.01 and *P* < 0.05, respectively) (Figure 4D). Our data confirm recent reports of increased PD-L1 expression on monocytes in sepsis (36), which was an independent predictor of death (37). Within the whole pool of monocytes, the percentage of Ly6C^hi^ inflammatory monocytes was significantly higher in the scrambled control miRNA–treated group compared with the control group (scrambled control vs. control: *P* < 0.05), suggesting a sepsis-specific increase. This increase was alleviated by anti–miR-93-5p therapy (scrambled control vs. anti–miR-93-5p: *P* <0.05) (Figure 4E). PD-L1 expression on Ly6C^hi^ cells also showed a significant therapy-specific reduction (scrambled control vs. anti–miR-93-5p: *P* < 0.05) (Figure 4F). In accordance with earlier reports (38), we observed a sepsis-specific decrease in Ly6C^lo^ monocytes (scrambled control vs. control: *P* < 0.001; scrambled control vs. sham: *P* < 0.05; anti–miR-93-5p vs. control: *P* < 0.05), while the PD-L1 expression on Ly6C^lo^ monocytes was constant between groups (Supplemental Figure 11, B and C).

The role of macrophages in sepsis is not well established, although tumor-associated macrophages were reported to be responsible for tumor progression in mice after surviving sepsis (39). In our model, the percentage of circulating CD11b^+^F4/80^+^ macrophages was not modified by the treatment (Supplemental Figure 11D), but F4/80^+^MRC1^+^ macrophage percentages were significantly higher in mice with sepsis that were treated with scrambled miRNA or anti–miR-93-5p compared with sham-operated mice (*P* < 0.01 and *P* < 0.05). Additionally, we observed a clear tendency toward lower F4/80^+^MRC1^+^ macrophages levels in the anti–miR-93-5p–treated group compared with the scrambled control miRNA–treated group (Figure 4G). The percentage of F4/80^+^PD-L1^+^ macrophages was significantly higher in mice treated with scrambled control miRNA compared with the percentage in anti–miR-93-5p–treated mice or sham-operated mice (*P* < 0.01 for both). The levels of PD-L1 expression were similar between the control and anti–miR-93-5p groups (Figure 4H).

We also analyzed myeloid-derived suppressor cells (MDSCs), both granulocytic (G-MDSCs) and monocytic (M-MDSCs), and did not observe any significant differences between groups (Supplemental Figure 11, E and F). Finally, the percentage of CSF1R^–^ neutrophils slightly increased during sepsis, especially in the anti–miR-93-5p–treated group compared with the control group (*P* < 0.05) (Supplemental Figure 11G), while the percentage of CSF1R^+^ neutrophils did not change between groups (Supplemental Figure 11H).

By adjusting for multiple testing using the FDR (for lymphoid and myeloid cell lineages separately), we observed that anti–miR-93-5p therapy mainly regulated 2 subsets of cells compared with scrambled control miRNA treatment: it increased the CD4^+^ Tem subset (FDR < 0.05) (Figure 4B), and it decreased CSF1R^+^PD-L1^+^ monocytes (FDR < 0.05) (Figure 4D). Anti–miR-93-5p therapy interfered with both the innate and adaptive immune cell–mediated phases of the response during sepsis: the inflammation phase was driven by innate immune cells, and the immunoparalysis by the dysfunctional T cell response (Figure 4I).

Furthermore, in this cohort of mice, we analyzed the histopathology of the essential organs of CLP-induced sepsis model mice treated with anti–miR-93-5p or scrambled miRNA and of control mice 24 hours after induction of sepsis. We observed no specific histopathological changes between the 3 groups, indicating no anti–miR-93-5p–induced drug toxicity (Supplemental Figure 12).

### Identification of sepsis-relevant miR-93 target genes.

In order to directly identify miR-93 target genes in immune cells, we created a miR-93–KO cell line. To this end, we consulted the Cancer Cell Line Encyclopedia (CCLE) database and found that B and T cell–derived cell lines most highly express miR-93. Among hematological malignancies, high levels of miR-93 are especially reported in B and T cell acute lymphoblastic leukemia (B/T-ALL), acute and chronic myeloid leukemia (AML and CML), and diffuse large B cell lymphoma (DLBCL) (Supplemental Figure 13A). Next, we validated the expression of miR-93-5p in JURKAT (T-ALL), THP-1 (AML), OCI-LY10 (DLBCL), and MEC-1, MEC-2, and HG3 (CLL) cells by reverse transcription quantitative PCR (RT-qPCR). We found that, by far, the highest expression of miR-93-5p was in JURKAT cells (Supplemental Figure 13B) and therefore selected these cells to generate the KO model.

Next, we used the CRISPR/Cas9 system to create miR-93–KO clones from parental JURKAT T cells. As mentioned above, miR-93 is part of a cluster that includes miR-25 and miR-106b within intron 12 of *MCM7* (Supplemental Figure 13C). To create miR-93–KO cells, we designed 2 sgRNAs spanning the mature sequence of miR-93-5p. Transfection of these sgRNAs together with the Cas9 protein into JURKAT T cells created heterozygous and homozygous deletions that were 24 bp in size (Supplemental Figure 13D). Two homozygous miR-93–KO clones (KO 1 and KO 2) as well as a CRISPR-treated, but unaffected, miR-93^+/+^ control clone (control) were selected for further analysis. Sanger sequencing confirmed the respective 24 bp deletion encompassing the miR-93-5p mature sequence in each miR-93–KO clone (Supplemental Figure 13E). The expression of miR-93-5p was completely abolished in KO 1 and KO 2 cells compared with parental and control cells, while the levels of neighboring miR-25-3p and miR-106b-5p (Supplemental Figure 13F) and the level of the protein encoded by the host gene *MCM7* (Supplemental Figure 13G) remained unaffected. The growth rate of KO clones was slightly slower (doubling time of 27.7 ± 4.3 hours in KO 1 and 28.3 ± 5.3 hours in KO 2) than that of parental cells (23.2 ± 2.7 hours) and control cells (25.3 ± 3.3 hours) (Supplemental Figure 13H). miR-93-5p KO did not cause noticeable changes in cell morphology (Supplemental Figure 13I).

**Figure 5:**
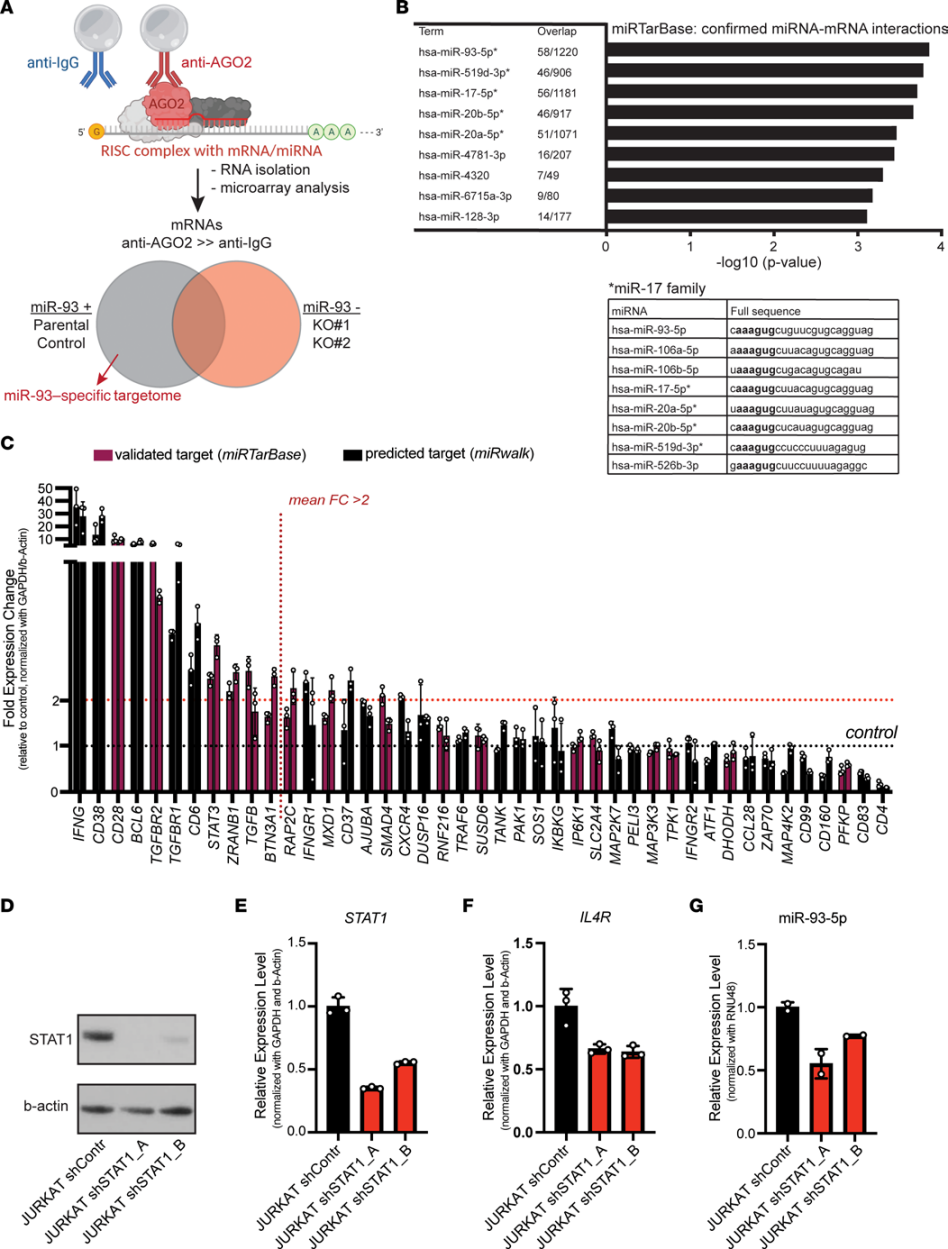
Identification of miR-93-5p target genes in sepsis and miR-93-5p upstream regulation. (**A**) Schematic of the AGO2 IP method. Beads coupled to anti-AGO2 antibody or control (IgG) antibody are used to pull down the RISC complex including bound miRNA/mRNA interactors in the presence or absence of miR-93 expression. Putative miR-93 targets (*n* = 579) were defined as being enriched in the AGO2 IP of parental or control cells but not miR-93–KO cells. (**B**) Pathway analysis on 570 putative miR-93 target genes showed strong enrichment for previously known mRNA targets (miRTarBase) of miR-93 or other miR-17 family members that share the same seed sequence. The top 9 most enriched are shown. (**C**) Forty-three genes with immune functions that were identified in the AGO2 IP and validated (purple) or predicted (black) targets of miR-93-5p were assessed in JURKAT miR-93–KO cells compared with control by RT-qPCR. Bars indicate the mean fold change (FC) in gene expression in the KO 1 and KO 2 samples relative to control (control = 1). (**D**) STAT1 protein expression measured by Western blotting in cell lysates of JURKAT shControl, JURKAT shSTAT1_A, and shSTAT1_B cells. (**E**) *STAT1* mRNA expression, (**F**) *IL4* mRNA expression, and (**G**) miR-93-5p measured by RT-qPCR in JURKAT shControl, and JURKAT shSTAT1_A, and shSTAT1_B cells.

Using this JURKAT miR-93–KO model, we applied the AGO2 ribonucleoprotein IP followed by microarray analysis (AGO2 RIP ChIP) (40) to identify miR-93-5p target mRNAs. This methodology uses beads coupled to either an anti-AGO2 antibody or an IgG control to pull down the RNA-induced silencing complex (RISC) together with any miRNA-interacting mRNAs. To define target genes of miR-93-5p, we assessed mRNA transcripts enriched in the AGO2 IP fraction of JURKAT parental or control cells (where miR-93-5p is present) but not in the 2 KO clones (where miR-93-5p is absent) by microarray (Figure 5A). The specific enrichment of AGO2 protein in the IP fractions of anti–AGO2 antibody–mediated, but not control anti–IgG antibody–mediated, RIP was validated by Western blotting (Supplemental Figure 14A). PCA showed separate clustering of input and IP samples in the first dimension, separation of anti-AGO2 and control IgG–mediated IP in the second dimension, and separation of KO 1/KO 2 clones from controls and parental cells in the third dimension (KO clones clustered very closely together; control vs. parental cells showed some distance from each other) (Supplemental Figure 14B). An average of 3,360 (range: 2,834–3,993) probes were found to be significantly enriched in the AGO2 IP compared with the IgG IP fraction per clone, with a mean fold change of approximately 7 (range: 1.5–497); these genes represent the entire miRNA targetome in JURKAT cells. Fewer genes (~1,730 on average) showed enrichment in the IgG IP over the AGO2 IP samples at a much lower fold change (mean: 2.5; range: 1.5–10.4), further confirming the specific pulldown of AGO2.

To select putative miR-93-5p target genes, we made 3 comparisons: parental versus KO (i.e., KO 1 and KO 2; 404 probes), control versus KO (236 probes), and parental/control versus KO (61 probes), together resulting in a total of 583 putative miR-93-5p target genes (Supplemental Table 3). In order to have a broad understanding of the regulatory potential of miR-93-5p, we performed pathway analysis using these 583 putative miR-93-5p target genes. We observed that the top regulated pathways played important roles in immune modulation and included important regulators of the immune response (Supplemental Figure 14C).

Cross-referencing of these 583 genes with confirmed miRNA-mRNA interactions (miRTarBase [ref. 41] via Enrichr [ref. 42]) showed a strong overrepresentation of confirmed interactors of miR-93-5p (58 genes, 10%) and miRNAs sharing the same seed sequence (miR-519d-3p, miR-17-5p, miR-20b-5p, miR-20a-5p) (Figure 5B). To further validate the identified miR-93-5p target genes, we selected 43 immune system–related genes that were IP enriched and either predicted or validated targets of miR-93-5p (41, 43) for validation by RT-qPCR. At the RNA level, 11 genes showed a greater than 2-fold upregulation in the KO 1 and KO 2 clones compared with control (Figure 5C), including 6 previously validated targets of miR-93-5p: *CD28* (44), *TGFBR2* (45–48), *STAT3* (49), *ZRANB1* (47), *TGFB* (50), and *BTN3A1* (47). In summary, we identified the full targetome of miR-93 in T cells that contains immune genes that could be involved in the therapeutic effect of anti–miR-93 therapy.

Next, we explored the upstream mechanism that controls the dysregulated expression of miR-93-5p in sepsis. We used the TransmiR, version 2.0, database and retrieved only the potential transcription factors (TFs) with the highest level of evidence (level 2) for which the ChIP-Seq data were derived from human blood. This double inquiry led to the discovery of 36 potential TFs (Supplemental Table 4). Among these was also STAT1, a TF well known to play a regulatory role in sepsis (51). Hence, we hypothesized that STAT1 could be a regulator of miR-93-5p in sepsis. We stably knocked down (KD) STAT1 in WT JURKAT cells using an shRNA. We confirmed the KD at protein (Figure 5D) and mRNA levels (Figure 5E) and checked the mRNA levels of a known downstream molecule of STAT1, *IL4R* (https://www.genecards.org/cgi-bin/carddisp.pl?gene=IL4R). As expected, the mRNA levels of *IL4R* dropped after STAT1 inhibition (Figure 5F). For *MCM7*, the host gene of miR-93-5p, we did not see any difference between shControl and STAT1-KD clones (Supplemental Figure 15A). With regard to miR-93-5p and the other 2 miRNAs in the cluster, we observed a significant drop in both shSTAT1 clones compared with the shControl (Figure 5G and Supplemental Figure 15, B and C). Finally, we analyzed the RNA-Seq data from WBCs of the 2 baboon models. In both the *S*. *aureus* and *E*. *coli* baboon models after the induction of sepsis, we observed an upregulation of *STAT1*, similar to the upregulation observed for miR-93-5p (Supplemental Figure 15, D and E).

Next, we checked if the miR-93-5p targetome detected using JURKAT cells was specific for T cells or could also explain the phenotypical changes in monocytes and macrophages that we observed in vivo. For this purpose, we screened 2 cell lines of the myeloid lineage, NB4 and THP-1, and observed that miR-93-5p expression was slightly higher in NB4 (acute promyelocytic leukemia, APL, or AML FAB M3) cells (Supplemental Figure 16A). Thus, we used this cell line to create a second miR-93–KO model using the CRISPR/Cas9 method. Out of the multiple clones with homozygous deletions of 24 bp size (Supplemental Figure 16B), we further analyzed 2 homozygous miR-93–KO clones (KO 1 and KO 2) as well as a CRISPR-treated, but unaffected, miR-93^+/+^ control clone (control) (Supplemental Figure 16C). As expected, the expression level of miR-93-5p in the KO clones was below the detection threshold, while miR-25-3p and miR-106b-5p were slightly overexpressed compared with expression levels in parental and control cells (Supplemental Figure 16D). Additionally, we did not observe any changes in MCM7 protein expression between the different clones (Supplemental Figure 16E). Next, we performed RT-qPCR for immune system–related genes that were IP enriched and analyzed in JURKAT cells. Several of these genes were not expressed in NB4, being highly specific for lymphocytes, including the top upregulated ones: *CD28* and *IFNG*. Using the same thresholds, we detected 2 other genes, *CD83* and *TRAF6*, which were not upregulated in JURKAT cells (Supplemental Figure 16F).

Hence, miR-93-5p had different targets in cells derived from the lymphoid and myeloid cell lineages, and the upregulation of these targetomes seemed to play important roles in immune modulation.

### miR-93-5p target genes respond to sepsis in baboons.

**Figure 6:**
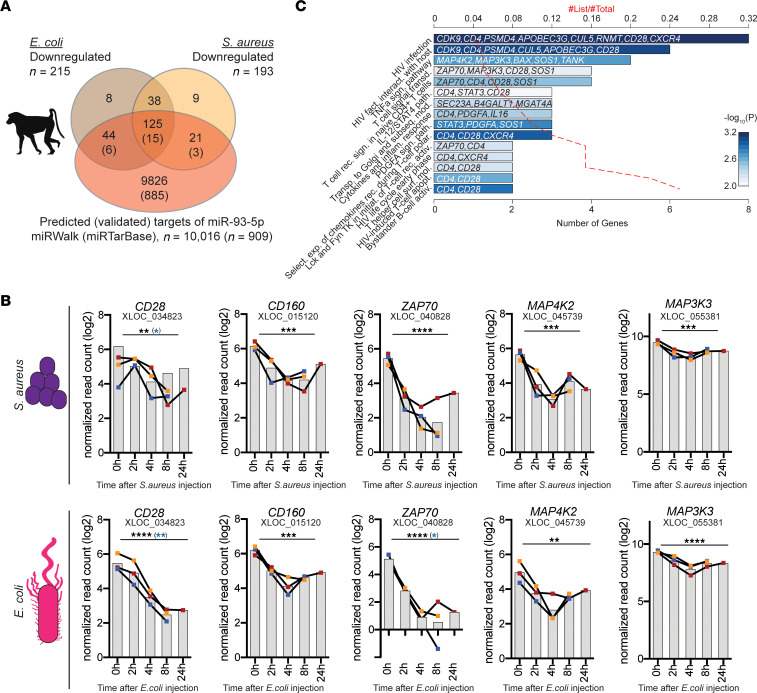
Confirmation of dysregulated miR-93-5p target genes in 2 baboon sepsis models. (**A**) The 579 putative miR-93 target genes were overlapped with genes that showed an opposite expression (i.e., downregulation) with miR-93 levels in the 2 baboon models (gray and yellow circles). Of the 465 genes that could be assigned to a baboon ortholog (of 579 IP enriched genes; 80%), 215 and 193 were downregulated upon injection with *E*. *coli* and *S*. *aureus*, respectively. In addition, this set of genes was overlapped with predicted and validated targets of mir-93-5p as extracted from miRWalk and miRTarBase (red circle). (**B**) Downregulation of *CD28*, *CD160*, *ZAP70*, *MAP3K3*, and *MAP4K2* upon sepsis induction in the *S*. *aureus*– and *E*. *coli*–mediated baboon models. **Q < 0.01, ***Q < 0.001, and ****Q < 0.0001, by moderate *t* test. Results were also adjusted to the subject effects using the LMM; significant *P* values after adjustment are highlighted in blue. (**C**) Pathway analysis of the 190 genes that were IP enriched, downregulated in either *E*. *coli*– or *S*. *aureus*–induced sepsis, or both in baboons and predicted targets of miR-93-5p. BioPlanet 2019 pathways with a *P* value of less than 0.01 are shown. *CD28* and *STAT3* are validated targets of miR-93-5p. fact., factor; interact., interaction; sign., signaling; transd., transduction; rec., receptor; path., pathway; Transp., transport; subseq., subsequent; mod., modification; inflam., inflammatory; Select., selective; exp., expression; rec., receptors; polar., polarization; initiat., initiation; activ., activation; surf., surface; mol., molecular; apopt., apoptosis.

To further solidify our approach to identifying sepsis-relevant targets of miR-93-5p, we used the phylogenetic conservation of miRNA targeting (52). As miR-93-5p expression was upregulated in baboons upon sepsis induction (Supplemental Figure 3, A and B), we next examined how many of the AGO2 IP–identified 583 putative miR-93-5p targets also showed lowered expression levels in the sepsis baboon models by genome-wide gene expression. Of note, because the RNA was extracted from WBCs, these data mainly reflect the changes of the lymphoid lineage. Of the 465 (80%) genes that could be assigned to an orthologous gene in baboons, 245 were downregulated (215 in *E*. *coli*, 193 in *S*. *aureus*, and 163 in both models) (Figure 6A). Cross-referencing with predicted (*n* = 10,016) and validated (*n* = 909) targets of miR-93-5p (43) identified 190 (77.5%) and 24 (9.8%) genes, respectively. This again included the major T cell signaling molecule *CD28*, which showed a highly significant reduction in both the *S*. *aureus* (*Q* = 0.0032) and *E*. *coli* (*Q* = 0.000042) models (Figure 6B). In addition, *CD160* a predicted target of miR-93-5p, also showed significant downregulation in both models (*S*. *aureus*, *Q* = 0.00057; *E*. *coli*, *Q* = 0.00013) (Figure 6B). Pathway analysis of the 190 predicted miR-93-5p targets revealed enrichment of key T cell signaling and polarization pathways (Figure 6C).

Analysis of the mRNA sequences using miRmap (53) revealed 7 possible miR-93-5p interaction sites within the 3′-UTR of *CD28*, one of which was previously validated by crosslinking immunoprecipitation (CLIP), coupled with high throughput sequencing (HITS-CLIP) in HeLa cells (MIRT684931) (44), while the 3′-UTR of *CD160* has 3 predicted binding sites. Furthermore, we observed that in both baboon sepsis models, 3 other miR-93-5p targets, *ZAP70*, *MAP3K3*, and *MAP4K2*, were downregulated after induction of sepsis (Figure 6B). We also detected 6 miR-93-5p targets that were downregulated only in the *E*. *coli* sepsis model: *STAT3*, *CDK9*, *TANK*, *BAX*, *CD4*, and *CXCR4* (Supplemental Figure 17A), and 3 others in the S. *aureus* sepsis model: *PDGFA*, *SOS1*, and *IL16* (Supplemental Figure 17B). We performed an additional statistical analysis, adjusting for the subject effects using the linear mixed-effects model (LMM). This analysis pointed out the central role played by *CD28*, which was the only miR-93-5p target downregulated after induction of sepsis in both models in this analysis: *S*. *aureus* (*P* < 0.05) and *E*. *coli* (*P* < 0.01) (Figure 6B). Furthermore, we observed downregulation of *ZAP70* and *SOS1* in the *E*. *coli* model (*P* < 0.05) and of *PDGFA* in the *S*. *aureus* model (*P* < 0.0001) (Supplemental Figure 17, A and B).

These data together show the widespread effect of miR-93-5p upregulation in sepsis and that the identified target genes are conserved targets of miR-93-5p between humans and baboons that contribute to the therapeutic effects of anti–miR-93 inhibition.

## Discussion

Sepsis is a deadly disease with a dismal outcome (13), and there is currently no FDA-approved therapy based on the molecular mechanisms (11). Therefore, we hypothesized over a decade ago that a new type of transcripts, the short noncoding RNAs named miRNAs, are essential for sepsis pathogenesis and discovered the first cellular miRNA (20), as well as the first viral miRNAs (22) involved in sepsis in humans. From the present stage of knowledge, we took on the task of starting the development of miRNA-based sepsis therapeutics. Sepsis is characterized by a complex host immune response consisting of both a disproportionate proinflammatory response and immune suppression. Despite this discovery, no therapy aimed at modifying the dysregulated host immune response is currently approved (54). The dysregulated immune response is variable between individuals and is one of the reasons why multiple clinical trials have failed for this disease (10). Sepsis is far more common in older patients with comorbidities, and the disease in this group of patients is characterized by immunosuppression (11). We present compelling data supporting the concept that miR-93-5p is involved in the molecular pathophysiology of sepsis and could be targeted to improve survival, potentially more so for elderly patients with sepsis than for younger patients.

First, by using multiple mouse models and human samples, we selected miR-93-5p as a potential target for sepsis therapy. We noticed that miR-93-5p was upregulated in PBMCs of patients with sepsis and in plasma from a CLP-induced sepsis mouse model. We observed that injecting miR-K12-12*, a viral miRNA that we showed to be involved in the pathophysiology of sepsis by binding TLR8 (22), induced the upregulation of miR-93-5p. We also checked the expression of miR-93 in different immune cells and observed that miR-93 was expressed across all different cell types, supporting the idea that it may play a role in immune cell regulation.

Second, miR-93-5p expression was upregulated in both Gram^+^ and Gram^–^ baboon sepsis models, supporting the idea that its dysregulation in sepsis is not dependent on the type of causal bacteria.

Third, we noticed that sepsis survival for patients with cancer was associated with a drop in miR-93-5p expression in whole blood at day 7 after the diagnosis of sepsis. Additionally, we showed that plasma levels of miR-93-5p correlated with important clinical sepsis parameters like the SOPED score and the ALC. Further studies will be necessary to examine whether this phenomenon is specific for cancer patients with sepsis or for a broader population of patients with comorbidities. The literature regarding miR-93-5p expression in patients with sepsis is scarce. Recently, Möhnle et al. reported that miR-93-5p was upregulated in T cells of patients with sepsis but did not correlate with T cell immunoparalysis (55). Yang et al. showed that miR-93-5p levels were downregulated in the serum of sepsis patients and played a role in sepsis-induced acute kidney injury (56). Moreover, miR-93-5p is an oncogenic miRNA that is upregulated in the serum of patients with cancer (57). Similar to other sepsis biomarker studies (58), the different results among these reports can be explained by the different comorbidities of the patients with sepsis, the origin of samples, and the time points of sampling.

Fourth, prophylactic and therapeutic treatment of older mice with anti–miR-93-5p led to a significant improvement in overall survival compared with scrambled miRNA–treated control mice. The effects are still significant but at lesser values in the younger treated mice compared with the scrambled miRNA–treated controls. Thus, the older the mice, the larger the difference in median survival induced by anti–miR-93-5p therapy. Because it is known that the response of immune cells is age dependent (59), we hypothesized that anti–miR-93-5p could affect the cellular immune response.

Fifth, we report for the first time to our knowledge the differences in the peripheral immune response of mice treated with anti–miR-93-5p compared with scrambled control miRNA–treated mice. The peripheral immune response of anti–miR-93-5p–treated mice compared with scrambled control–treated mice was characterized by: (a) increased frequencies of CD4^+^ and CD8^+^ Tem cells, (b) decreased percentages of Ly6C^hi^ monocytes and F4/80^+^ MRC1^+^ macrophages, and (c) downregulation of PD-L1 on CSF1R^+^ monocytes, Ly6C^hi^ inflammatory monocytes, and F4/80^+^ macrophages. Sepsis is associated with apoptosis of Tem cells in both the periphery and lymphoid organs, inducing immunoparalysis (60), and anti–miR-93-5p therapy appeared to block this phenomenon in the periphery and especially for the CD4^+^ Tem subtype of lymphocytes. Ly6C^hi^ monocyte levels are usually lower in sepsis compared with sham and play a protective role against renal tissue damage during sepsis (61). The percentage of circulating Ly6C^hi^ monocytes was higher in the scrambled miRNA–treated group compared with the sham-operated and anti–miR-93-5p–treated groups, suggesting that anti–miR-93-5p could improve monocyte recruitment to peripheral tissues. Macrophages in sepsis are often excessively produced and activated and are an important source of cytokines, which are associated with high mortality in sepsis (62). Anti–miR-93-5p decreased the frequency of F4/80^+^ MRC1^+^ macrophages, which could be beneficial. Upregulation of PD-L1 on immune cells is a known feature of sepsis and is one of the mechanisms that induce immunosuppression. Even after using additional statistical analysis, we were able to observe a decrease in PD-L1 expression on CSF1R^+^ monocytes in the treated sepsis group. PD-L1 has been proposed as a potential innovative therapeutic target in sepsis (63); hence, our results reveal a potentially new miRNA-based strategy to target it.

Finally, we analyzed the mechanism through which downregulation of miR-93-5p prolongs survival in sepsis. By using a human CRISPR-engineered T cell model, we observed that high levels of miR-93-5p downregulated multiple important T cell signaling molecules. The baboon sepsis models pointed out the membrane molecule *CD28* in particular. *CD28* was previously reported as a target of miR-93-5p in fibroblasts (44). Recent studies have shown that CD28 agonism can prolong the survival of CLP-induced immunologically experienced sepsis mice via IL-10 released by T cells (64). Our results are in line with this observation, and we may speculate that the older mice might have been exposed to prior infections similar to those in immunologically experienced mice, but this hypothesis needs future confirmation. Our analysis, even after adjustment for the subject effects in the baboon sepsis models, revealed as well that an intracellular signaling molecule, *ZAP70*, was downregulated after induction of sepsis, especially in the *E*. *coli* model. ZAP70 is a tyrosine kinase relaying the initial step in signal transduction upon T cell receptor (TCR) stimulation and is thus central to T cell activation. Accordingly, high levels of *ZAP70* were inversely correlated with organ failure (SOFA) scores and mortality in patients with sepsis (65). Furthermore, we showed that STAT1 is probably an upstream inducer of miR-93-5p. *STAT1*-deficient mice are resistant to CLP-induced septic shock (51), and it is tempting to speculate that this effect is partially mediated by the lack of miR-93-5p induction in the immune cells of these mice. These data, taken together, revealed a direct negative regulation of major T cell signaling molecules by miR-93-5p that is in line with the expansion of effector T cells observed in mice upon anti–miR-93-5p treatment. Hence, high miR-93-5p levels in sepsis simultaneously suppress multiple avenues of T cell activation, mainly that of CD4^+^ T cells, inducing a state of anergy that is reversible by miR-93-5p inhibition. We also performed CRISPR KO in cells of the myeloid lineage. We observed that the targetome of miR-93-5p differed between the myeloid and lymphoid cell lineages, revealing new targets in the NB4 cell line: *CD83* and *TRAF6*. CD83 is preformed in macrophages and monocytes and is presented on the cell surface upon stimulation with LPS (66), playing important roles in maintaining the balance between inflammation and immune tolerance (67). TRAF6 is a downstream molecule of TLR4 signaling and a signal transducer of the NF-κB pathway. In sepsis models, it was shown that impaired autoubiquitination of TRAF6 in macrophages leads to an immune-deficient phenotype (68).

Furthermore, we acknowledge several limitations of this study. First, we did not perform any long-term toxicity studies regarding side effects that could be induced by anti–miR-93-5p therapy. Second, some of the screening data and in vivo data come from small patient cohorts and a small number of animals. We tried to overcome this limitation by confirming the initial results in multiple animal models and patient cohorts. Third, the exact pharmacological proprieties of anti–miR-93 therapy need to be further elucidated. Fourth, the sepsis therapy discussed here may have greater efficiency in older patients with preexisting comorbidities (cancer), but future sepsis research needs to be directed toward specific subgroups of patients. Finally, we did not perform any studies regarding the diagnostic value of miR-93-5p, which is also known to be dysregulated in several cancer types (48, 57). Plasma miR-93-5p may be responsible for part of the multiple pathogenic mechanisms that generate different clinical phenotypes of sepsis. Nevertheless, measurement of plasma miR-93-5p can be a companion diagnostic for selecting sepsis patients who would be likely to benefit from anti–miR-93-5p therapy.

In conclusion, this study presents miR-93-5p as a potential new therapeutic target for sepsis. By blocking miR-93-5p in sepsis, the peripheral immune response is partially restored, and overall survival is prolonged, especially in elderly organisms. Our data reveal that PD-L1, another therapeutic target being investigated in sepsis, was overexpressed on macrophages and monocytes. Hence, combining anti–miR-93-5p and anti–PD-L1 therapies could represent another future direction to explore, aiming to maximize the survival rate in sepsis.

## Methods

A detailed description of the materials and methods used in this study is provided in Supplemental Methods (see the full uncut gels used to generate the results at the end of Supplemental Methods).

### Study approval.

All mouse experiments were approved and supervised by the IACUC of MDACC under the protocol “Role of ncRNAs in Sepsis” (00001483-RN02). The animals were cared for according to the guidelines of the American Association for Accreditation of Laboratory Animal Care (AAALAC) and the US Public Health Service Policy on Humane Care and Use of Laboratory Animals.

Baboon (*Papio ursinus*) samples were collected from historical sepsis experiments of previous studies performed in 2016 at the Animal Research Center, Faculty of Health Sciences, University of the Free State, Bloemfontein, South Africa. The experiments were performed in compliance with the NIH Office of Laboratory Animal Welfare (OLAW), the Animal Welfare Act, and the *Guide for the Care and Use of Laboratory Animals, 8th edition* (National Academies Press, 2011) and were approved by the Interfaculty Animal Ethics Committee of the University of the Free State, Bloemfontein, South Africa (UFS-AED 2016/0007). Reuse of samples from previous experiments is consistent with the principles of the 3R framework for performing humane animal research that is key to nonhuman primate experimentation.

The observational studies of cancer patients with sepsis (cohort 1 and cohort 2) were conducted at the Emergency Department of MDACC under approved clinical research protocols (PA13-0666 for cohort 1 and 2018-0757 for cohort 2). The protocols were approved by the IRB of MDACC, and the studies were conducted in compliance with US federal regulations, the Health Insurance Portability and Accountability Act of 1996 (HIPAA), and the Declaration of Helsinki. A signed informed consent was obtained from each participant.

### Data availability.

The data that support the findings of this study are available within the article and its Supplemental Tables and Figures. Baboon RNA-Seq data can be found in the NCBI’s Gene Expression Omnibus (GEO) database (GEO GSE221652); mRNA microarray analysis data for JURKAT parental, control, and miR-93 KO 1 and KO 2 cells can be found in the NCBI’s GEO database (GEO GSE223458); and miRNA microarray data for PBMCs from patients with sepsis and control individuals were previously deposited in the public repository ArrayExpress (accession E-TABM-713).

## Author contributions

MPD, EFM, MW, and GAC designed research. MPD, EFM, MW, KO, R Bayraktar, YL, SA, HZ, AQ, IK, MS, LP, AMT, SM, AT, MK, MWA, SJR, RS, RSK, CRA, PPB, R Basar, and MTSB performed research. MPD, EFM, MW, KO, EK, MC, KL, XL, MF, CI, CG, ZL, and SCY analyzed data. DH, CV, MTSB, KR, FL, SCY, and GAC contributed to discussions, data analysis, editing of the text, and financial support of the study, and MPD, EFM, MW, and GAC wrote the manuscript. All authors edited the manuscript. MPD is the first author in the list of shared first authors, as he initiated the project, wrote the first draft of the manuscript, performed animal experiments, and completed key experiments. EFM is the second author in the list of shared first authors, as he initiated the project, developed the mouse animal models of sepsis, and completed key experiments. MW is the third author in the list of shared first authors, as she performed most of the mechanistic cell-based studies and completed the remaining experiments to bring this manuscript to completion.

## Supplementary Material

Supplemental data

## Figures and Tables

**Table 1:**
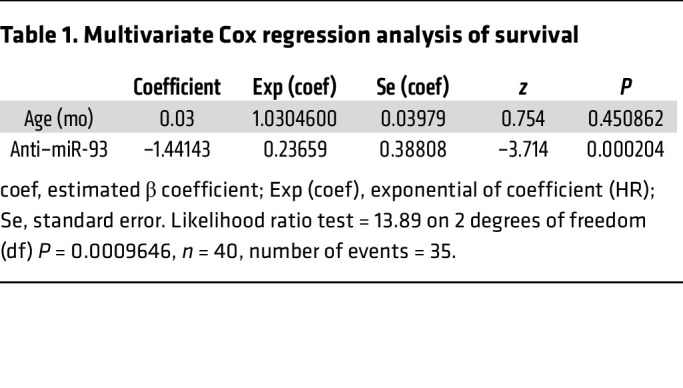
Multivariate Cox regression analysis of survival

## Version 1 06/01/2023

In-Press Preview

## Version 2 07/17/2023

Electronic publication

## Version 3 10/17/2023

Addition of CPRIT Acknowledgement
