# Supplementary material for: Anti–miR-93-5p therapy prolongs sepsis survival by restoring the peripheral immune response
Source: J Clin Invest. 2023 Jul 17;133(14):e158348. doi: 10.1172/JCI158348 (PMC10348769; doi:10.1172/JCI158348)
Supplement: Supplemental data [file jci-133-158348-s008.pdf]

Supplemental Material

Anti-miR-93-5p therapy prolongs sepsis survival by restoring the peripheral immune response

Supplemental Methods *Page 2*

Supplemental Figures *Page 16*

Supplementary Figure 1 *Page 16*

Supplementary Figure 2 *Page 17*

Supplementary Figure 3 *Page 19*

Supplementary Figure 4 *Page 21*

Supplementary Figure 5 *Page 23*

Supplementary Figure 6 *Page 24*

Supplementary Figure 7 *Page 26*

Supplementary Figure 8 *Page 27*

Supplementary Figure 9 *Page 28*

Supplementary Figure 10 *Page 30*

Supplementary Figure 11 *Page 32*

Supplementary Figure 12 *Page 34*

Supplementary Figure 13 *Page 35*

Supplementary Figure 14 *Page 38*

Supplementary Figure 15 *Page 39*

Supplementary Figure 16 *Page 40*

Supplementary Figure 17 *Page 42*

Supplemental Tables

Supplementary Table 1 *Page 44*

Supplementary Table 2 *Page 45*

Supplementary Table 3 *Page 47*

Supplementary Table 4 *Page 67*

Supplementary Table 5 *Page 69*

Supplementary Table 6 *Page 70*

References *Page 72*

Supplemental Methods

Cecal ligation and puncture-induced murine sepsis model

Male and female C57BL/6 mice of different ages were purchased from The Jackson Laboratory. One day before surgery, the lower abdomen of all mice was shaved with an electric trimmer. Before surgery, mice were anesthetized using an inhalational anesthetic (isoflurane). After anesthesia, the abdomen of the mice was disinfected with alcohol prep pads.

Next, an approximately 1.5 cm-long skin midline incision was made with a surgical scissor without entering the peritoneal cavity. After this initial incision, a small scissor was used to extend the incision and enter the peritoneal cavity. Afterward, by using anatomical forceps, the intestines were mobilized until the cecum was localized and exteriorized. We performed a large ligation of the cecum (high-grade sepsis), close to the ileocecal valve (75% of the cecum were under the ligation), therefore we were expecting a 100% lethality of untreated mice within maximum 4 days after CLP (1). The cecum was perforated by a single, through-and-through puncture between the tip of the cecum and the ligation. After removing the needle, feces were extruded and the cecum was placed back in the peritoneal cavity. Two to three sutures were applied in order to close the peritoneum, fasciae, and abdominal muscles, and the skin was closed using metallic clips. In order to avoid wound infection, antibiotic ointment was applied over the closed incision. Additionally, after the procedure, mice received 100 µl of sterile PBS via intraperitoneal injection for hydration. Postoperative analgesia was given to all mice. All mice with sepsis that survived were euthanized 72 hours after the induction of sepsis. As controls, we used sham-operated mice, who underwent the same procedure only without ligation and puncturing of the cecum.

KSHV-miR-K12-12*-induced inflammation model

Male and female C57BL/6 mice were purchased from The Jackson Laboratory. Eight-week-old mice were injected intraperitoneally with 200 µg/kg of miR-K12-12* mimic or the same dose of scramble miRNA. The oligonucleotides were incorporated into neutral 1,2-dioleoyl-sn-glycero-3-phosphatidylcholine (DOPC) nanoliposomes. Briefly, DOPC and miRNA (miR-K12-12* mimic or scramble miRNA) were mixed in the presence of excess tertiary butanol at a ratio of 1:10 (w/w) miRNA/DOPC. Tween-20 was added to the mixture in a ratio of 1:19 Tween-20:miRNA/DOPC. The

mixture was vortexed, frozen in an acetone/dry ice bath, and lyophilized. Before *in vivo* administration, this preparation was hydrated with PBS at a concentration of 200 µg/kg to achieve the desired dose in 100 µL per injection. Twenty-four hours after intraperitoneal miRNA injection, the mice were euthanized by terminal intracardiac blood draw under anesthesia.

***In vivo* treatment with anti-miR-93-5p**

Male and female C57BL/6 mice were purchased from The Jackson Laboratory. We used mice of three different age groups for the survival experiments: 4.5-month-old (adult mice), 8-month-old (middle age/mature mice), and 16-month-old (old mice).

For all therapeutic experiments, we used anti-miR-93-5p and scramble anti-miRNAs. The dose of miR-93-5p and scramble anti-miRNA was 200 µg/kg, as described previously (2). The oligonucleotides were incorporated into neutral DOPC nanoliposomes. These were administered via intraperitoneal injection 24 hours before and 2 hours after the induction of sepsis using the CLP method. Mice in any group were monitored continuously after CLP until their deaths.

When death was confirmed (no heartbeat), intracardiac blood was drawn, and the mice were subjected to necropsy. Only mice that showed signs of peritonitis were included in the survival analysis. Liver, lung, heart, spleen, and kidney samples were formalin-fixed, frozen in optimal cutting temperature (OCT) medium, to prepare slides for microscopy, and frozen at -80 ^{o}C for later RNA extraction.

The animals were cared for according to the guidelines of the American Association for Accreditation of Laboratory Animal Care and the US Public Health Service Policy on Humane Care and Use of Laboratory Animals.

Murine cell preparations and flow cytometry

Mature, gender-matched, 8-month-old control mice (no procedure was performed), sham-operated mice, and CLP mice treated with anti-miR-93-5p or scramble RNA were sacrificed 24 hours after surgery. Intracardiac blood was collected from mice, and cells were isolated. Erythrocytes from blood samples were lysed by incubation in ammonium chloride solution (NH_4Cl) lysis buffer (NH_4Cl 0.15 M, KHCO3 10 mM, Na2EDTA 0.1 mM, pH 7.2-7.4) for 5 min at room temperature. After blocking fragment crystallizable (Fc) receptors with Fc block (BD Biosciences) for 10 minutes at room temperature, cells

from intracardiac blood were stained with the antibodies (15 min. at 4°C) listed in **Supplementary Tables S5**. Cells were analyzed with a BD LSRFortessa X-20 flow cytometer. Ten-color flow cytometry phenotype analysis of live myeloid cell singlets and 13-color flow cytometry phenotype analysis of live lymphoid cell singlets were performed using an LSRFortessa X-20. To develop the multi-color flow cytometry panels, we used antibody-capture beads (UltraComp eBeads, Invitrogen, Waltham, MA, USA) for single-color compensation controls. A Live/Dead Fixable Aqua Dead Cell Stain Kit (Thermo Fisher Scientific) was used first to gate out dead cells. Further gating adjustments were made based on fluorescence-minus-one (FMO) controls.

Baboon model of *Staphylococcus (S.) aureus* and *Escherichia (E.) coli* sepsis

Healthy baboons (*Papio*), 8-20.2 kg body weight with a leukocyte count less than 13,000/µL and hemoglobin >10 g/dL, were randomly distributed between experimental groups. Gram positive *S. aureus* subspecies, aureus Rosenbach (ATCC 12598) was purchased from the American Type Culture Collection (Manassas, VA), and Gram negative *E. coli*, serotype B7-086a:K61, was *in-house* prepared the day before the challenge. Animals were challenged with 3×10^{10} heat-inactivated *S. aureus* (a lethal dose) (3) or $1\text{-}2 \times 10^{10}$ CFU/kg *E. coli* (4) given by IV infusion over 2 hours. The heat-inactivated *S. aureus* model is a sepsis model (3, 5), where the host response is induced by pathogen associated molecular patterns (PAMPs) released from the bacterial wall, such as the peptidoglycan (6-8) and lipoteichoic acid (9) that are major contributors to sepsis and coagulopathy in Gram positive bacterial infections. Heat treatment inactivates thermo-labile exotoxins and prevents bacterial proliferation, allowing for standardization of the bacterial dose. Previous work from our Oklahoma group has shown that the baboon challenge with comparable amounts of either live or heat-inactivated *S. aureus* can induce sepsis that can be equally lethal (10). Hence, the heat-inactivated *S. aureus* model is a true sepsis model comparable with live *S. aureus* models.

The time point at which the bacterial infusion began was designated as T0. Eight hours after the start of the bacterial infusion (T+8), the animals were returned to the recovery cage and observed until they exhibited signs of unrecoverable organ failure and septic shock, at which time they were humanely euthanized. Surviving animals were euthanized on day 7. Blood was drawn from all animals before the

bacterial infusion (T0), two hours later (T+2), four hours later (T+4), six hours later (T+6), eight hours later (T+8), 24 hours later (T+24), and at the time of euthanasia, when tissue samples were collected from select organs and processed for microscopy.

Patients

The patients from cohort #1 were sampled as part of a prospective observational study of cancer patients with sepsis that was conducted at the Emergency Department of MD Anderson Cancer Center. Inclusion Criteria for Septic Cancer Patients: (1) High suspicion of infection by the emergency physician or clinical/diagnostic evidence of infection; (2) Two or more of the following SIRS criteria (a. leukocytes > 12,000/mm^3 or < 4,000/mm^3 or > 10% immature (band) forms, provided that no filgrastim or pegfilgrastim was administered within 30 days; b. heart rate > 90 beats/min; c. respiratory rate > 20 breaths/min or partial pressure of CO2 < 32 mmHg; d. oral temperature > 38°C or < 36°C or axillary temperature > 37°C or < 35°C); and (3) age of 18 years and older.

Exclusion Criteria for Septic Cancer Patients: (1) inability to give informed consent or a person who has power of attorney for medical decision is not available; (2) moribundity; (3) active hospice care; and (4) active "Do Not Resuscitate" or "Do Not Intubate" orders. Blood samples (5 ml in EDTA) was drawn for analysis at the time of enrollment. Additional samples were drawn 1 day and 7 days later for comparison with the first blood sample if the patient was still hospitalized.

The patients from cohort #2 were from a different and independent prospective observational study of cancer patients with sepsis that was conducted at the Emergency Department of MD Anderson Cancer Center. Briefly, all cancer patients who presented to the Emergency Department between 9/12/2019 and 2/10/2022 with high clinical suspicion of sepsis by the emergency physician and two or more of the SIRS criteria were included in this study. Inclusion and exclusion criteria were similar to the first study (cohort #1). For this study, and after obtaining informed consent, blood samples (in EDTA tubes) were collected for analysis at the time of the patient's emergency department presentation. Demographic, emergency department presentation (including vital signs), clinical, cancer-related, and laboratory variables related to the visit were collected from the electronic health records. The Acute Physiology and Chronic Health Evaluation (APACHE II) (11), the Sequential Organ Failure Assessment

(SOFA) (12), Mortality in Emergency Department Sepsis (MEDS) (13), and Septic Oncologic Patients in the Emergency Department (SOPED) (14) were also calculated and analyzed for each patient.

For T cell isolation experiments, buffy coats from three healthy donors were provided by the Gulf Coast Regional Blood Center (barcodes: W0446 21 368914, W0446 21 351244, W0446 21 351246).

Isolation and activation of primary human T cells

Standard Ficoll-mediated isolation of PBMCs was followed by PAN-T cell isolation using the human Pan T cell Isolation Kit (Miltenyi Biotec, Cat# 130-096-535) according to the manufacturer's instructions. T cells were cultured and activated as described previously (15) in RPMI 1640 plus 10% human AB serum at 37°C with 5% CO2, together with 6.25μL/1 million cells Dynabeads™ Human T-Activator CD3/CD28 for T Cell Expansion and Activation (Thermo Fisher, Cat# 11161D) and 50IU/mL human IL2 (R&D, Cat# 202-IL). IL2 supplemented RPMI 1640 plus 10% human AB serum medium was refreshed every 48h. Medium was refreshed, and cell density was assessed using hemacytometer-mediated counting every 48h.

RNA extraction from plasma, peripheral blood mononuclear cells, and whole blood

Total plasma, peripheral blood mononuclear cells (PBMC), white blood cells (WBCs), and whole blood RNA were extracted and reverse transcribed as previously described (16). RNA was obtained from 100 µL of plasma, PBMCs, or whole blood using the total RNA purification kit (NorgenBiotek, Cat. #37500) according to the manufacturer's instructions. At the end of the extraction process, RNA was eluted in 50 µL elution solution, and RNA concentrations and quality were determined using NanoDrop-1100. For the normalization of plasma sample-to-sample variation in the RNA isolation step, the *C. elegans*, cel-miR-39-3p and cel-miR-54-3p, (ThermoFisher SCIENTIFIC, Cat # A25576, and Cat #A25576), 25 fmol of each in a total volume of 1 µL, were used. The geometric means (17) of the Ct values of cel-miR-39-3p and cel-miR-54-3p were used for normalization. For the normalization of sample-to-sample variation of RNA extracted from PBMCs and whole blood, U6 was used as an endogenous normalizer.

RNA extraction from solid tissues (liver, kidney, heart)

Solid organs were homogenized in liquid nitrogen before RNA isolation in Trizol (Life Technologies Corporation, Carlsbad, CA, USA). Next, the RNA isolation included prolonged precipitation and centrifugation steps to preserve the small RNA fractions. Phase separation was made by incubation on ice for 30 min, followed by centrifugation at 12,000 g for 20 min at 4 °C. Total RNA samples were precipitated overnight on ice and centrifuged at full speed (21,000 g) for 30 min at 4 °C. For the normalization of sample-to-sample variation of RNA extracted from organs, U6 was used as an endogenous normalizer.

cDNA synthesis

miRNAs: RNA was reverse transcribed using the TaqMan® miRNA Reverse Kit (Applied Biosystems, Cat. #4366596) in a 10-μL reverse transcription (RT) reaction containing 10 ng of RNA, 0.1 μL of 100 mM dNTPs, 0.67 μL of Multiscribe reverse transcriptase, 1 μL of 10× RT buffer, 0.13 μL of RNase inhibitor, and 1 μL of 5 × miRNA-specific stem loop RT primer (Applied Biosystems). Reverse transcription was performed in a Bio-Rad DNA engine with the following program: 16°C for 30 minutes, 42°C for 30 minutes, and 85°C for 5 minutes. The cDNA was diluted 1:3 and stored at −20°C until analysis.

Genes: The High-Capacity cDNA Reverse Transcription Kit (Applied Biosystems, Cat# 4368814) was used according to the manufacturer's instructions using 1 μg RNA input in a 20μL reaction. Reverse transcription was performed using the following program: 25°C for 10min, 37°C for 2h, 85°C for 5min, and then 4°C on hold. cDNA was stored at −20°C until analysis.

RT-qPCR profiling

miRNAs: The diluted cDNA (3 μL) was used as a template in a quantitative PCR (qPCR) reaction with a total final volume of 5 μL. DNA amplification was performed using TaqMan primers specific for each analyzed miRNA (together with SsoFastTM Probes Supermix (Bio-Rad Laboratories, Cat. #172-5231). The reaction started with an incubation period of 3 minutes at 95°C, followed by 40 cycles of 5 seconds at 95°C, and 30 seconds at 60°C. All experiments were performed in triplicate. Ct values beyond the upper limit of the measuring system are imputed as 40. The raw Ct values, for the plasma

samples, were normalized by the Ct values of cel-miR-54-3p and cel-miR-39-3p. For RNA extracted from PBMCs and solid tissues (liver, kidney, and heart), U6 was used as an endogenous control.

Genes: qPCR was performed using SsoAdvanced Universal SYBR Green Supermix according to the manufacturer's instructions with a cDNA input of 2ng and forward/reverse primer concentrations of 250nM in a 5-uL reaction. The reaction started with an incubation period of 3 minutes at 95°C, followed by 40 cycles of 15 seconds at 95°C and 30 seconds at 60°C. For both miRNAs and genes, the relative expression level of each miRNA was calculated using the equation $2^{-\Delta CT}$. Primer sequences are provided in **Supplementary Table S6.**

Multiplex cytokine assay

Blood samples were processed for plasma cytokine level analyses. Plasma cytokine levels were measured using the MILLIPLEX MAP Mouse Cytokine Magnetic Bead Panel (MCYTOMAG-70K) according to the manufacturer's protocol. The analysis includes 16 pro-inflammatory [granulocyte-colony stimulating factor (GCSF), eotaxin, granulocyte-macrophage colony-stimulating factor (GM-CSF), interferon-gamma (IFNG), interleukin 1 alpha (IL1A), IL1B, IL2, IL5, IL6, IL9, IL12 (p40), IL12 (p70), IL17, macrophage inflammatory protein 1alpha (MIP1A), MIP1B and tumor necrosis factor-alpha (TNFA)] and three anti-inflammatory cytokines (IL4, IL10, and IL13).

Histological assessment of tissue injury

For quantitative analysis of tissue damage, we analyzed the lung, heart, kidney, liver, and spleen of 16-month-old CLP-mice treated with anti-miR-93-5p or scramble miRNA form the survival experiment. For each mouse, all organs were formalin-fixed and paraffin-embedded in a single block. The embedded tissue blocks were cut into 4-μm-thick sections, and stained with hematoxylin and eosin (H&E), Periodic acid–Schiff (PAS), Masson's trichrome, or Jones' stain.

The heart tissue damage was scored using a modified method from the one described by Kishimoto et al. (18). Briefly, three different parameters were analyzed: myocardial necrosis, fibrosis, and immune infiltrates. Each of the three parameters was scored from 0 to 4 (0 = absent, 1 < 25% of the tissue, 2 = 25-50%, 3 = 50-75%, and 4 > 75%) on an ordinal scale with a step size of 0.5. The tissue damage score represents the sum of the three parameters.

The lung tissue pathological assessment was performed as previously described (19). Briefly, we scored from 0 to 3 (0 = absent, 1 = mild, 2 = moderate, 3 = severe) using an ordinal scale with a step size of 0.5 for the presence of exudates, hyperemia/congestion, immune infiltrates, intraalveolar hemorrhage/debris, and cellular hyperplasia. The tissue damage score represents the sum of all five parameters.

The kidney damage pathological scoring was performed similarly to the one used by Yu et. al. (20). Using a 0 to 4 ordinal scale with a step size of 1 scoring system (0 = none, 1 = 0–20%, 2 = 20%–50%, 3 = 50%–70%, 4 > 70%), we looked for tubules that displayed necrosis, loss of brush border, interstitial edema, vacuolization, tubule dilatation, and immune infiltrates. For the analysis of the tubules and glomeruli, the PAS and Jones' silver stain were essential. The tissue damage score represents the sum of the six parameters.

The liver damage was analyzed using a modified method from the one described previously by Martin et al. (21). Four parameters - immune infiltration, hypoperfiusion to necrosis, central vein congestion, and sinusoidal congestion were scored from 0 to 3 (0 = none, 1 = mild, 2 = moderate, 3 = severe) on an ordinal scale with a step size of 0.5 scale. The tissue damage score represents the sum of the four parameters.

For spleen tissue damage analysis, we used a modified version of the protocol reported by Karamese et al. (22). We analyzed five different parameters: increased immune infiltrate, apoptotic cells, hemosierin deposits, hemorrhage, and thrombi, which were scored from 0 to 4 (none - 0, mild - 1, moderate - 2, severe - 3, and more severe – 4) on an ordinal scale with a step size of 0.5. The tissue damage score represents the sum of the four parameters.

Additionally, we noted an increasing number of PAS-positive foamy immune cells in the lungs and spleens of mice, and these were scored separately. We counted PAS-positive immune cells from five high-power fields with the most abundant PAS-positive cell infiltrate (40x magnification), and averaged the five data points, and reported as the number of PAS-positive macrophages/HPF.

The slides were evaluated using an Olympus BX46 (Olympus Europe). Histological images were acquired with the PANNORAMIC 1000 digital slide scanner (3DHISTECH).

TUNEL assay

Because we morphologically observed apoptotic cells (shrinking and fragmentation of cells) in the spleens and lungs of mice, we used the TUNAL assay to further analyze apoptosis. For this purpose, we built two tissue microarrays (TMA), one including the spleens and a second one including the lungs of 8-month-old mice from the survival study. Representative areas with immune cells showing morphological features of apoptosis were identified on H&E slides and transferred to tissue microarrays (TMAs) with one core per sample. Each core measured 1 mm in diameter. Spleen and lung TMA sections of 4µm were used for the TUNEL assay by means of the HRP-DAB TUNEL staining kit (ab206386), and the slides were counterstained by methyl blue following the protocol provided by the TUNEL staining kit. For the quantification TUNEL assay, tissues were analyzed by quantifying at least 3-5 images at 40x magnification (HPF) per core. The data were reported as number of positive cells per HPF. The slides were evaluated using an Olympus BX46 (Olympus Europe). Histological images were acquired with the PANNORAMIC 1000 digital slide scanner (3DHISTECH).

Cell culture

MEC-1 and MEC-2 serial sister cell lines are $CD5^{low/-}$ CLL cell lines established from a CLL patient in prolymphocytoid transformation to B-cell prolymphocytic leukemia obtained from DMSZ. The CLL HG3 cell line was kindly provided by Dr. Anders Rosen (Department of Clinical and Experimental Medicine; Division of Cell Biology, Linköping University, Linköping, Sweden). THP-1 (AML) and JURKAT (T-ALL) cells were purchased from ATCC. NB4 cells (acute promyelocytic leukemia) were obtained from DMSZ. These cells were cultured in RPMI-1640 medium (Sigma, St. Louis, MO) with 10% fetal bovine serum and penicillin-streptomycin (15 µg/ml; Sigma-Aldrich, St. Louis, MO) at 37°C in a 5% CO2 incubator. OCI-LY10 cells were obtained from DMSZ and were cultured in 80% Iscove's MDM (Sigma, St. Louis, MO) + 20% fetal bovine serum and penicillin-streptomycin (15 µg/ml; Sigma-Aldrich, St. Louis, MO) at 37°C in a 5% CO2 incubator. All cell lines were validated by the Cytogenetics and Cell Authentication Core at MD Anderson using short tandem repeat DNA fingerprinting and were

regularly negative for *Mycoplasma* contamination (MycoAlert Mycoplasma Detection Kit; Lonza, Morristown, NJ).

CRISPR/Cas9-mediated miR-93 knockout

The method described by Rosenlund *et al.* (23) was used for generating miR-93 knockout clones from JURKAT cells and NB4 using two sgRNAs. Electroporation was performed using the Amaxa Cell Line Nucleofector Kit V (Lonza, Cat# VCA-1003) according to the manufacturer's instructions using program X-001. For knockout validation, DNA from single cell-derived clones was isolated using the Genomic DNA Mini Kit (IBI Scientific, Cat# IB47202). PCR was performed using GoTaq Green Master Mix (Promega, Cat# M712) using the following program: 95°C for 2min, 40 cycles of (95°C for 30sec, 60°C for 30sec, 72°C for 2min), 72°C for 5min, and then 4°C on hold, and PCR products were assessed on 3% agarose gel. Sanger sequencing was performed by the MD Anderson DNA sequencing core facility using both forward and reverse primers. Sequences were analyzed using ChromasPro V2.6.6.

Sequences of sgRNAs and PCR primer sequences for validation by PCR and sanger sequencing are provided in **Supplementary Table S6**.

AGO2-RIP-Chip

AGO2-RIP was performed as described previously (24). In brief, EZview™ Red Protein G Affinity Gel Beads (Sigma, Cat# E3403) were blocked with 5% BSA and 500 μg/mL salmon sperm DNA (Sigma, D7656) in NT2 buffer (50mM Tris, pH7.4; 150mM NaCl; 1mM $MgCl_2$, 0.05% Nonidet P-40) for 1h at 4°C. Then, 10 μg of anti-AGO2 antibody or normal mouse IgG (**Supplementary Table S6**) was coupled to the affinity gel beads rotating overnight at 4°C. Beads were washed in NT2 buffer and resuspended in IP buffer (1xNT2 buffer, 40 U/μL RNAseOUT, 5 μL vanadyl ribonucleoside complex, 0.1 M DTT, 0.5 M EDTA). Per JURKAT clone and replicate, 30 million cells were lysed on ice in polysome lysis buffer (5 mM $MgCl_2$, 100 mM KCl, 10 mM Hepes, pH7, 0.5% Nonidet P-40, 1mM DTT, 40 U/μL RNAseOUT, and 1x Protease Inhibitor cocktail); the lysate was added to the beads in IP buffer. After an input sample was taken, the mix rotated overnight at 4°C before flow through and IP fractions were collected for RNA and protein extraction. RNA fractions were dissolved in Qiazol (Qiagen) and stored at -80°C until

isolation; protein fractions were dissolved in Laemmli buffer (BioRad) substituted with 2-mercaptoethanol, boiled at 100 °C for 10 min and stored at -20 °C until analysis. RNA was extracted using the miRNeasy mini (input fraction) or micro (IP fraction) kit (Qiagen, Cat# 217004 and 217084, respectively) according to the manufacturer's instructions. RNA quality was assessed using the Agilent Bioanalyzer 2100 using the Eukaryote Total RNA Nano Assay version 2.6.

Genome wide gene expression data analysis from *S. aureus* and *E. coli* baboon models

Bulk RNA data from white blood cells (WBCs) was collected on baboons under two different sepsis models (live *E. coli* and heat-inactivated *S. aureus*) and 4-5 time points (2, 4, 6, 8, and 24 hours after bacteria infusion). Three baboons were observed under each intervention model, and total RNA was extracted from their WBCs at each time point. RNA-sequencing was performed on a NextSeq 500 Flowcell, High SR75 (Illumina, San Diego, CA, USA) following library preparation using the QuantSeq 3' mRNA-Seq Library Prep Kit FWD for Illumina (Lexogen, Vienna, Austria). The library preparation and sequencing were performed by the Clinical Genomics Core at the OMRF. RNA-seq data processing followed the guidelines and standard practices of the ENCODE and modENCODE consortia. 75 bp single end raw sequencing reads, in a FASTQ format, were trimmed of residual adaptor sequences using Scythe software. Low-quality bases at the beginning or the end of sequencing reads were removed using sickle software, and then the quality of the remaining reads was confirmed with FastQC. Trimmed quality reads were aligned to the *Papio anubis* (olive baboon) genome reference (PapAnu2.0) using STAR v2.4.0h, and transcript-level read counting was performed with HTSeq v0.5.3p9. Read-count normalization and differentially expressed analyses were performed using the edgeR package from Bioconductor. Expression values normalized with the variance modeling at the observational level (voom) function were analyzed for differential expression using the standard functions of the limma package. The group option of the voom function was used to accommodate the serial data collection design, and time series analysis methods, implemented in R package MasigPro, were used to characterize the deregulation of genes related to sepsis over time. Moderate t test p-values were adjusted for multiple testing using the false discovery rate (FDR) method, and FDR < 0.05 was used to filter significant differentially expressed transcripts between conditions and across time. Annotations for

baboon transcripts and their human homologs were retrieved from the ensemble database using Biomart.

miRNA expression profiles from public resources

Global miRNA expression profiles (CCLE_miRNA_20180525.gct) were downloaded from the Cancer Cell Line Encyclopedia (CCLE; https://depmap.org/portal/download/). miR-93 expression was pooled by cancer subtype and displayed as normalized read counts.

Normalized miRNA expression data from $CD3^+$, $CD14^+$, $CD15^+$, $CD19^+$, and $CD56^+$ immune cell subsets were extracted from series GSE56590 using the following patient subsets with different time point measurements (n = 10): H1 (timepoints t0, t1, t2), H2 (t0), H3 (t0, t1, t2), H4 (t0), H5 (t0), and H6 (t0).

Microarray analysis

A microarray analysis was performed for each clone (i.e., JURKAT parental, Control, miR-93 KO#1 and KO#2) in duplicate for both input and IP fractions using the Applied Biosystems human Clariom™ S assay (Cat# 902927) according to the manufacturer's instructions. Relative signal intensities were extracted using Affymetrix feature extraction software version 1.1.0.1567. The CEL files, generated from Affymetrix RNA microarray image analysis software, were processed through Transcriptome Analysis Console 4.0 which normalizes (and applies the $\log_2$ function to) array signals using a robust multiarray averaging algorithm.

Approximately 16,000 microarray probes that were expressed above background in both duplicates of either the parental, control, or both miR-93 KO samples were selected for further analysis. Target genes of miR-93 were defined as being enriched in the AGO2-IP vs. the control IgG-IP fraction of the parental or control samples (i.e., miR-93-5p expressed) but not in either of the two generated knockout clones (i.e., miR-93-5p expression absent). Differentially expressed mRNAs in a comparative analysis were further identified by analysis of variance (eBay) with a P-value cutoff of 0.05 and a fold change more than 1.5 or less than -1.5.

MiRNA target prediction and pathway analysis

We obtained experimentally confirmed miRNA-mRNA interactions from miRTarBase (http://mirtarbase.mbc.nctu.edu.tw) and miRWalk 3.0 (http://zmf.umm.uni-heidelberg.de/apps/zmf/mirwalk2/). Integrated pathway analyses were performed with BioPlanet 2019 using Enrichr bioinformatics resources (http://amp.pharm.mssm.edu/Enrichr/). A P-value < 0.01 was considered significant. For visualizing and clustering the pathways from the AGO2-immunoprecipitation experiment, the Appyters scatter plot tool was used (25). The clusters were built using the Leiden algorithm. Points were plotted on the first two Uniform Manifold Approximation and Projection dimensions.

STAT1 knockdown

STAT1 shRNAs were purchased as ready-to-use pGFP-C-shLenti containing lentiviral particles from Origene (Herford, Germany; Cat# TL301349V). JURKAT cells were transduced with 4 anti-STAT1 shRNAs and 1 non-targeting control shRNA at a MOI of 10. GFP-positive cells were sorted at day 5 post-transduction using a BD FACS Aria III cell sorter. STAT1 knockdown was confirmed at the mRNA (RT-qPCR) and protein (Western blotting) levels. Results stemming from the two most efficient anti-STAT1 shRNAs and the non-targeting control shRNA were presented in the manuscript.

Western blotting

Protein concentration was quantified by the Bradford assay (Bio-Rad). In total, 20 ug of proteins were loaded on 4%–20% acrylamide Criterion TGXTM precast gels (Bio-Rad) and transferred to nitrocellulose membranes by the semidry method. The membranes were incubated overnight with the corresponding primary antibodies for MCM7 and GAPDH (normalizer) (**Supplementary Table S6**) and then incubated with the appropriate horseradish peroxidase–conjugated secondary antibody. Immunoreactivity was detected by incubation with ECL SuperSignal West Femto Substrate (ThermoFisher Scientific), and detected by the autoradiographic film.

Statistical analysis

Statistical analysis and graphical representation were performed with GraphPad Prism (version 8.1.2). P values < 0.05 were considered statistically significant. The Shapiro-Wilk normality test was performed for each group to assess whether the data were normally distributed. For normally distributed

data, a 2-tailed t test was used to compare mean values between different groups. When one of the groups did not pass the normality test, the Mann-Whitney-Wilcoxon nonparametric test was used to assess statistical differences between the different groups. For samples with matched repeated measures, we performed the ANOVA test with Geisser-Greenhouse correction for normally distributed data and the Friedman test if the data did not pass the normality test. Correlation analyses between expression data and clinical parameters were performed using the Pearson correlation test. We adjusted for multiple testing using the false discovery rate (FDR) (26). The statistical P values from all the cell lineages (lymphoid or myeloid) were obtained and adjusted by the p.adjust function in R. To adjust for the correlations of the data obtained from the same subject (baboon gene expression data), we applied a linear mixed effects model (LMM) (27) by specifying time as the covariate and baboon ID as the random effect. We used the most common correlation structure, exchangeable, to capture the correlation between the repeated measurements. The method was implemented using the lme4 package in R.

Survival curves were generated by the Kaplan-Meier method. For survival studies, a log-rank test was used. All values are expressed as mean and standard deviations (s.d.).

Supplemental Figures and Tables

Supplementary Figure 1

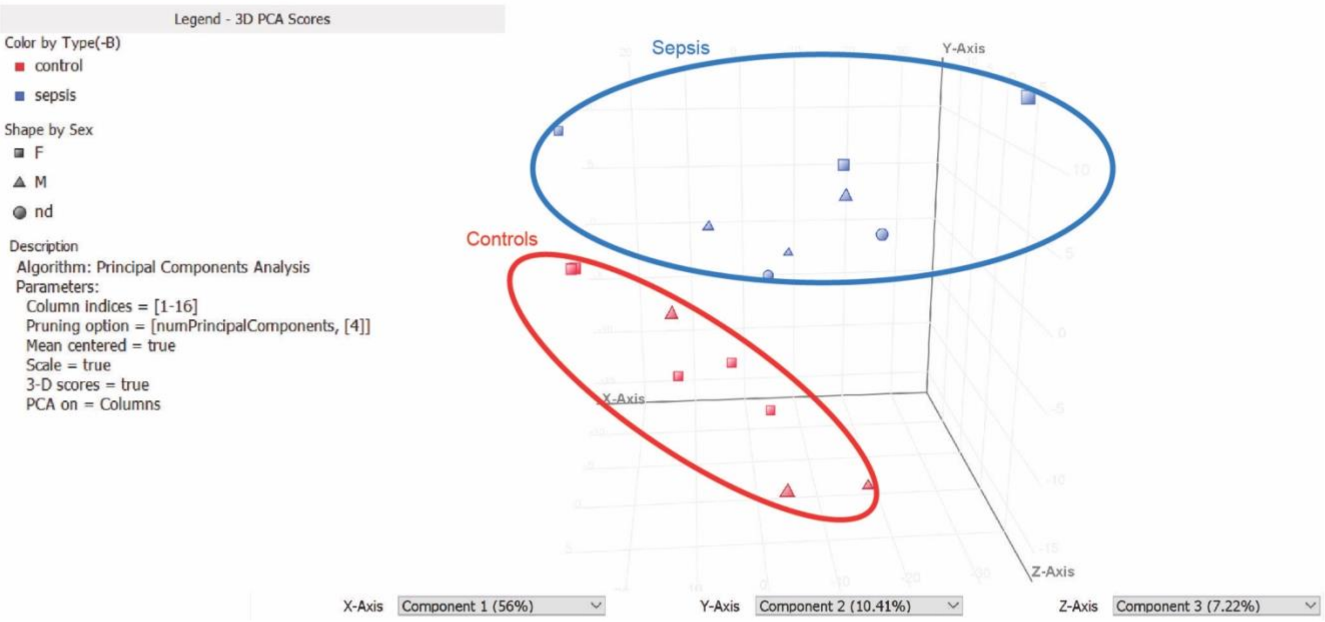

Supplementary Figure 1. Principal component analysis (PCA) plot. A scatter plot showing the coordinates of the samples on the three main principal components. Controls are represented as red dots; sepsis as blue dots.

Supplementary Figure 2

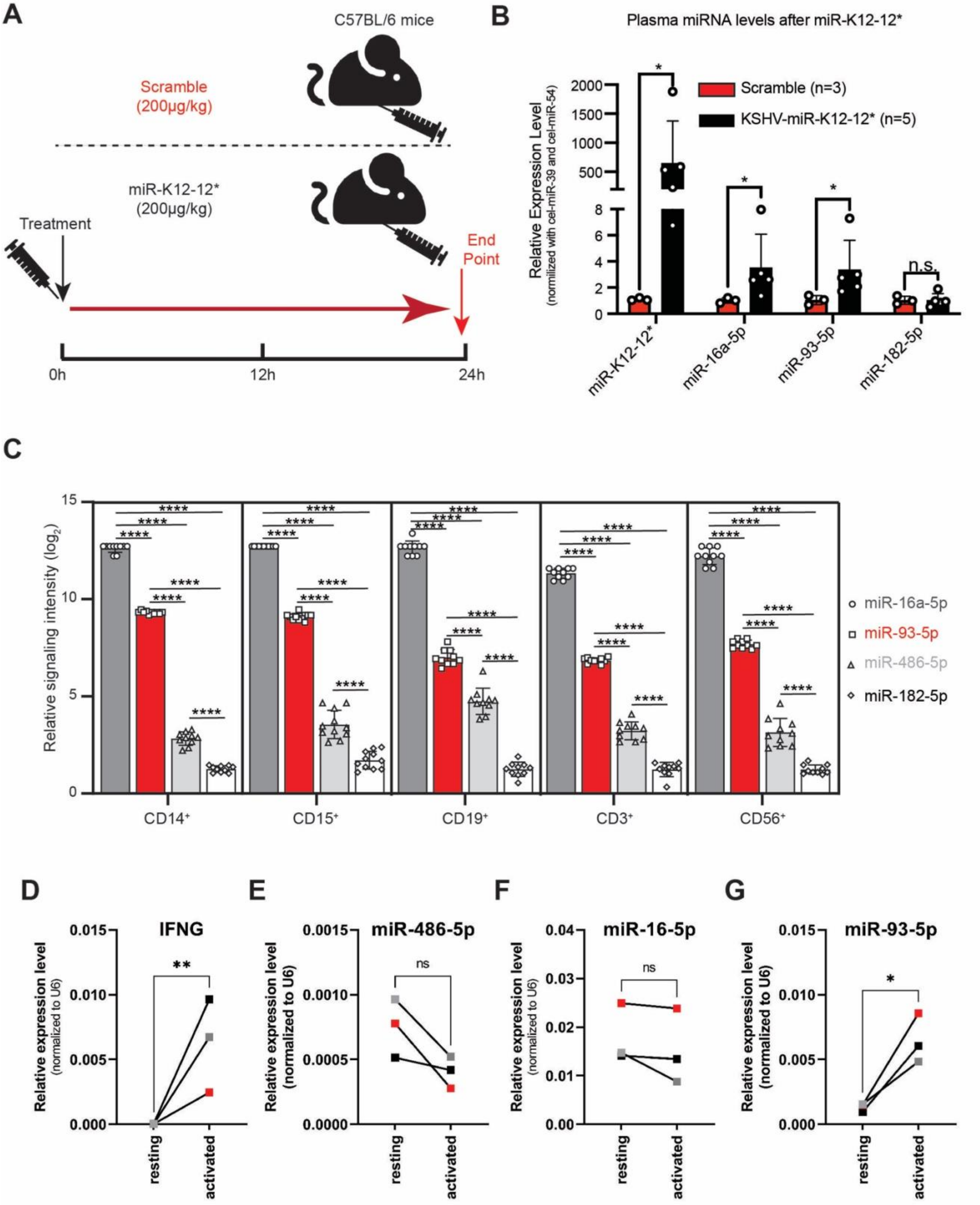

Supplementary Figure 2. MiR-93-5p is a potential therapeutic target in sepsis. (A) Eight-week-old male and female C57BL/6 mice were injected intraperitoneally with scramble miRNA (n = 3) or miR-K12-12* (n = 5); 24 hours after treatment, all mice were sacrificed. **(B)** Plasma levels of miR-K12-12*, miR-16a-5p, miR-93-5p, and miR-182-5p in mice injected with scramble miRNA (n = 3) compared to mice injected with anti-miR-93-5p (n = 5). The relative expression level was normalized to cel-miR-39-3p and cel-miR-54-3p. **(C)** Levels of the four sepsis-induced miRNAs across 5 different immune cell types isolated from 10 healthy donors. Data are presented as mean ± sd; paired t test, **** $P < 0.0001$. (**D – G**) Expression of *Interferon gamma (IFNG*) and sepsis miRNAs miR-486-5p, miR-16-5p, and miR-93-5p in resting and activated primary human T cells isolated from three healthy donors. RT-qPCR, mean ± error; paired t test, ns – non-significant, * $P < 0.05$, ** $P < 0.01$.

Supplementary Figure 3

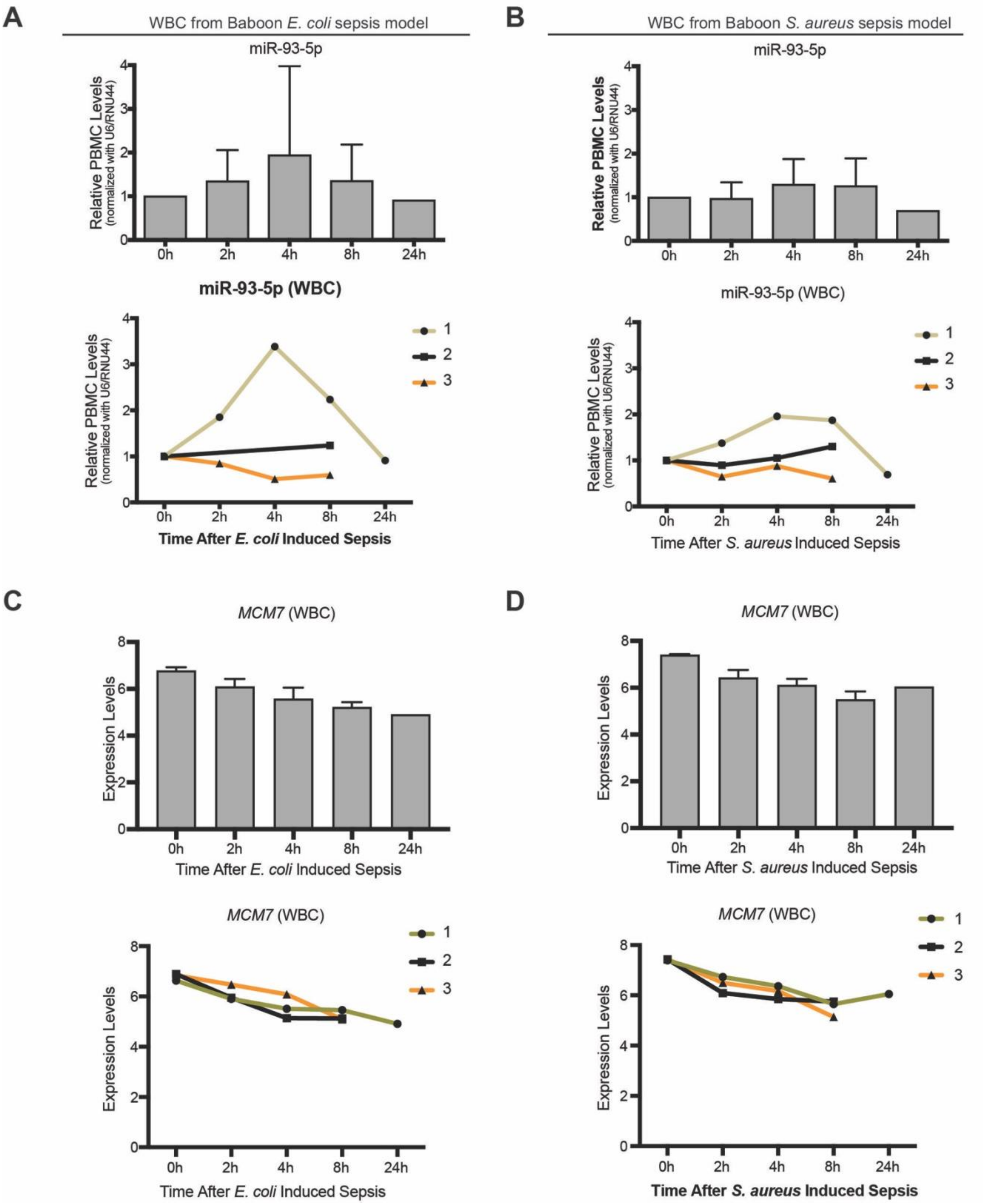

Supplementary Figure 3. MiR-93-5p and *MCM7* expression in WBCs from two baboon models of sepsis. (A) The levels of miR-93-5p in WBCs at different time points (before – 0h and 2h, 4h, 8h, and 24h after induction of sepsis) in an *E. coli* (Gram-negative) baboon sepsis model (n = 3) (upper panel). MiR-93-5p dynamics in WBCs from baboons inoculated with *E. coli* (n = 3) (lower panel). **(B)** The levels of miR-93-5p in WBCs at different time points (before – 0h and 2h, 4h, 8h, and 24h after induction of sepsis) in a *S. aureus* (Gram-positive) baboon sepsis model (n = 3) (upper panel). MiR-93-5p dynamics in WBCs from baboons inoculated with *S. aureus* (n = 3) (lower panel). **(C)** The levels of *MCM7* in WBCs at different time points (before – 0h and 2h, 4h, 8h, and 24h after induction of sepsis) in an *E. coli* (Gram-negative) baboon sepsis model (n = 3) (upper panel). *MCM7* dynamics in WBCs from baboons inoculated with *E. coli* (n = 3) (lower panel). **(D)** The levels of *MCM7* in WBCs at different time points (before – 0h and 2h, 4h, 8h, and 24h after induction of sepsis) in an *S. aureus* (Gram-positive) baboon sepsis model (n = 3) (upper panel). *MCM7* dynamics in WBCs from baboons inoculated with S. aureus (n = 3) (lower panel). Data are presented as means ± s.d.

Supplementary Figure 4

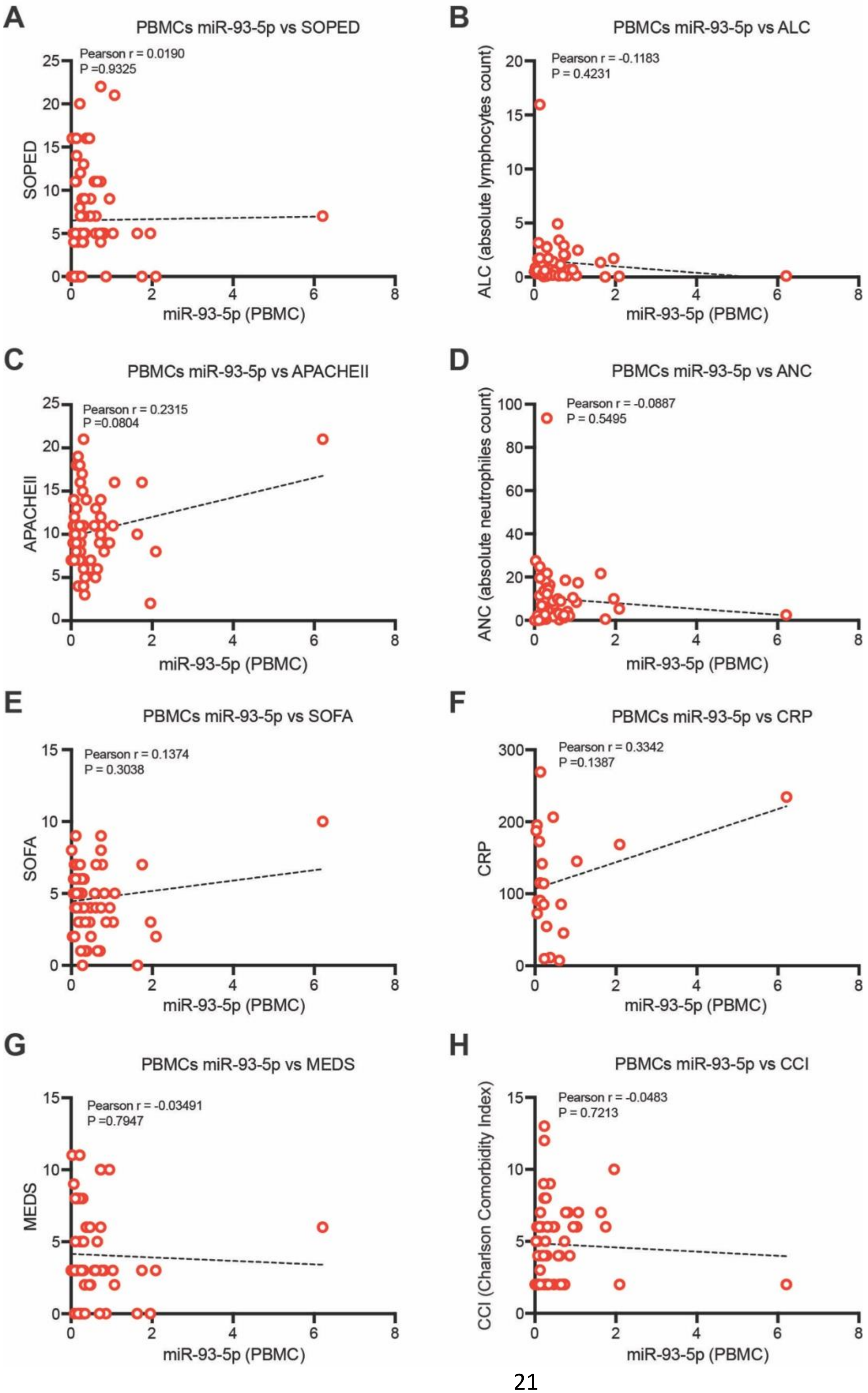

Supplementary Figure 4. Correlation between miR-93-5p levels in PBMCs and clinical variables in cohort #2 of sepsis patients with cancer. (A) Correlation between the level of miR-93-5p and the Septic Oncologic Patients in the Emergency Department (SOPED) score, (**B**) absolute lymphocytes count (ALC), (**C**) APACH II score, (**D**) absolute neutrophils count (ANC), (**E**) Sepsis-related Organ Failure Assessment (SOFA) score, (**F**) C reactive protein (CRP) level, (**G**) Mortality in Emergency Department Sepsis (MEDS) score, and (**H**) Charlson Comorbidity Index (CCI). Between the miR-93-5p expression level and each clinical parameter, a Pearson correlation was computed.

Supplementary Figure 5

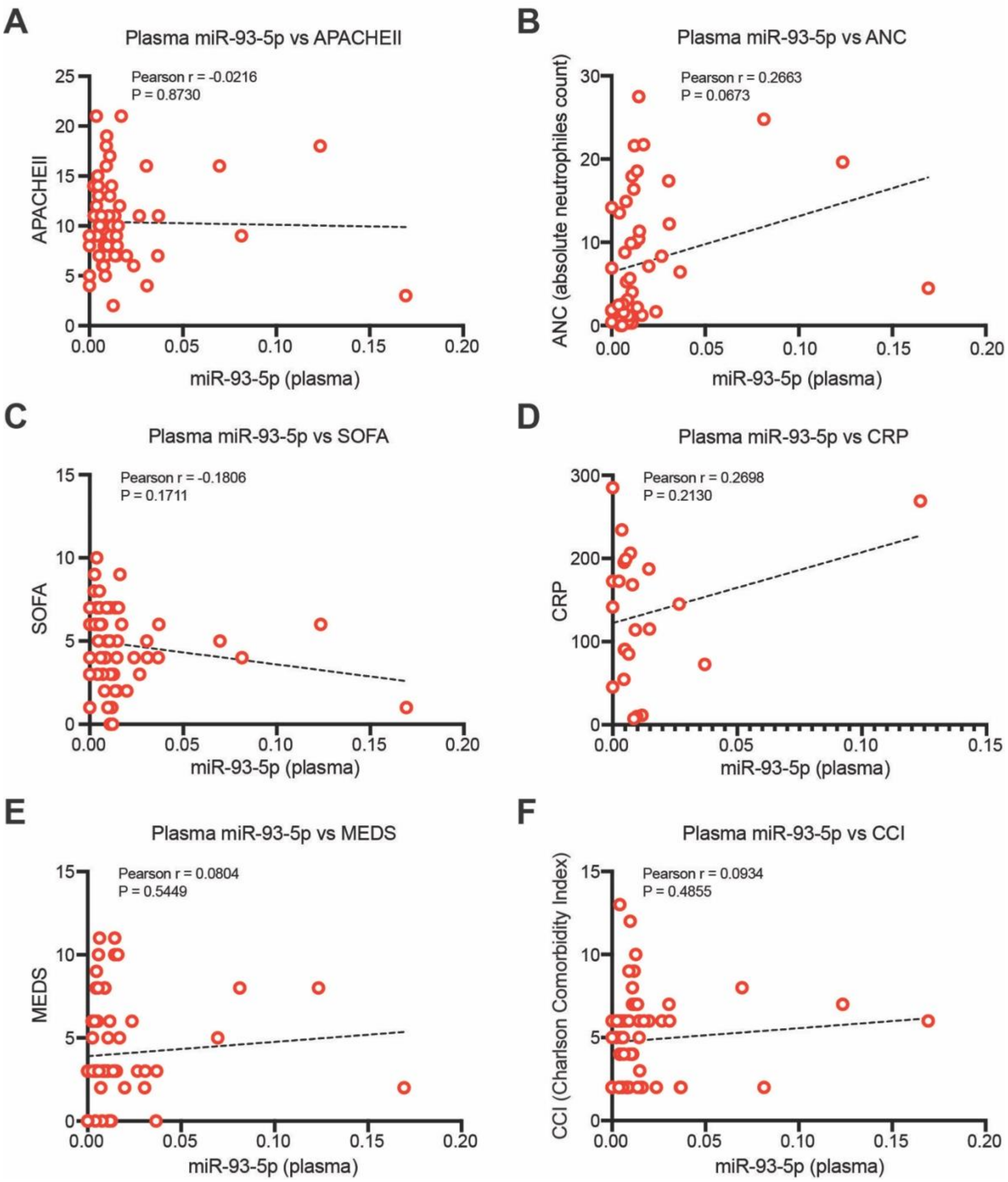

Supplementary Figure 5. Correlation between miR-93-5p levels in plasma and clinical variables in cohort #2 of sepsis patients with cancer. (A) Correlation between the level of miR-93-5p and APACH II score, (**B**) absolute neutrophils count (ANC), (**C**) Sepsis-related Organ Failure Assessment (SOFA) score, (**D**) C reactive protein (CRP) level, (**E**) Mortality in Emergency Department Sepsis (MEDS) score, and (**F**) Charlson Comorbidity Index (CCI). Between the miR-93-5p expression level and each clinical parameter, a Pearson correlation was computed.

Supplementary Figure 6

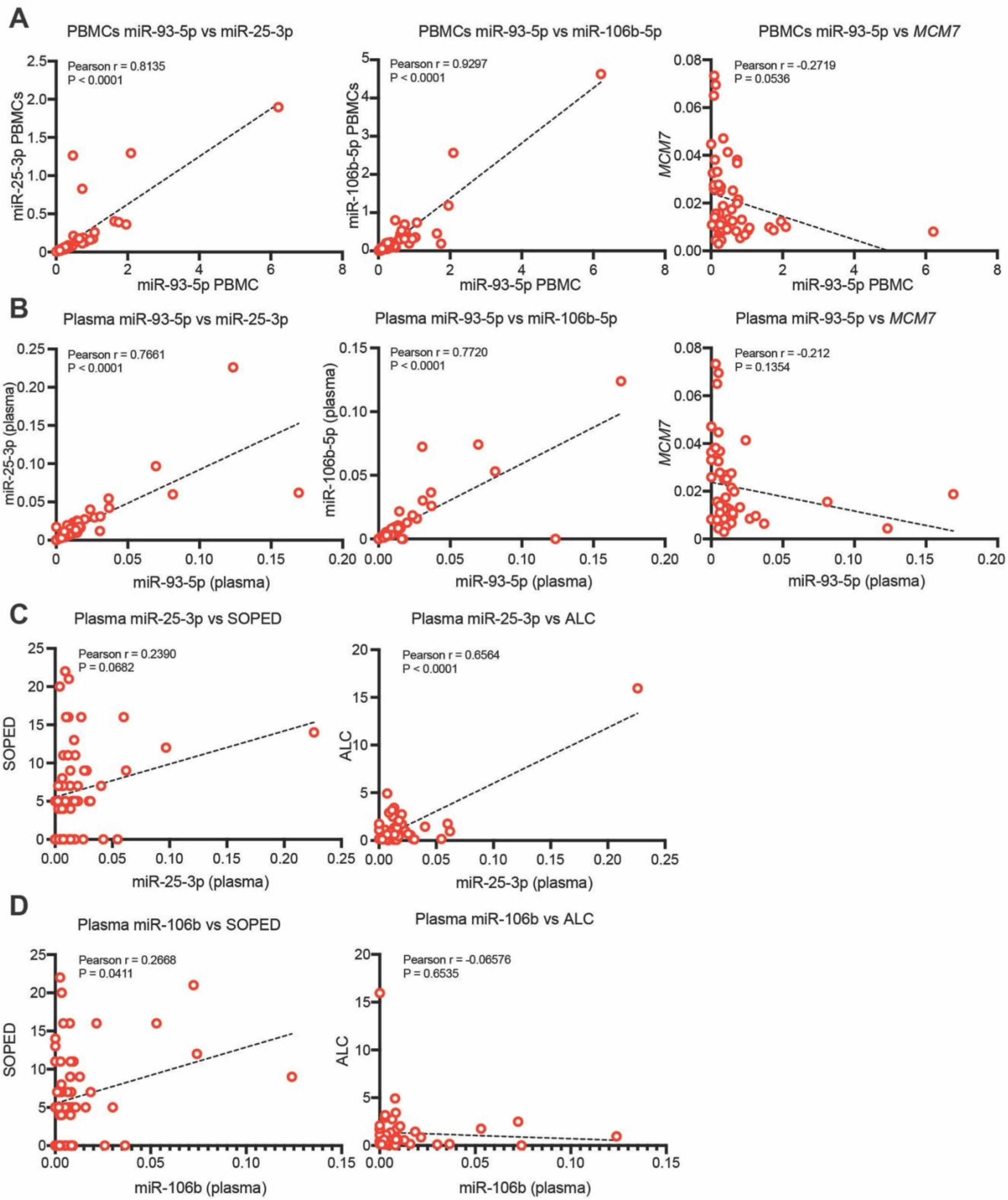

Supplementary Figure 6. Correlation between miR-93-5p levels in PBMCs and plasma and miR-25-3p, miR-106b-5p, and *MCM7* from the same biofluids in sepsis cohort #2. Correlation between miR-25-3p and miR-106b-5p levels in plasma and clinical variables in sepsis cohort #2. (A) Correlation between the levels of miR-93-5p and miR-25-3p, miR-106b-5p, and *MCM7* in PBMCs. (**B**) Correlation between the levels of miR-93-5p and miR-25-3p, miR-106b-5p, and *MCM7* in plasma. (**C**) Correlation between the level of miR-25-3p levels in plasma and Septic Oncologic Patients in the Emergency Department (SOPED) score and absolute lymphocytes count (ALC). (**D**) Correlation between the level of miR-106b-5p levels in plasma and SOPED score and absolute lymphocytes count (ALC). Between the miRNA expression level and MCM7/clinical parameters a Pearson correlation was computed.

Supplementary Figure 7

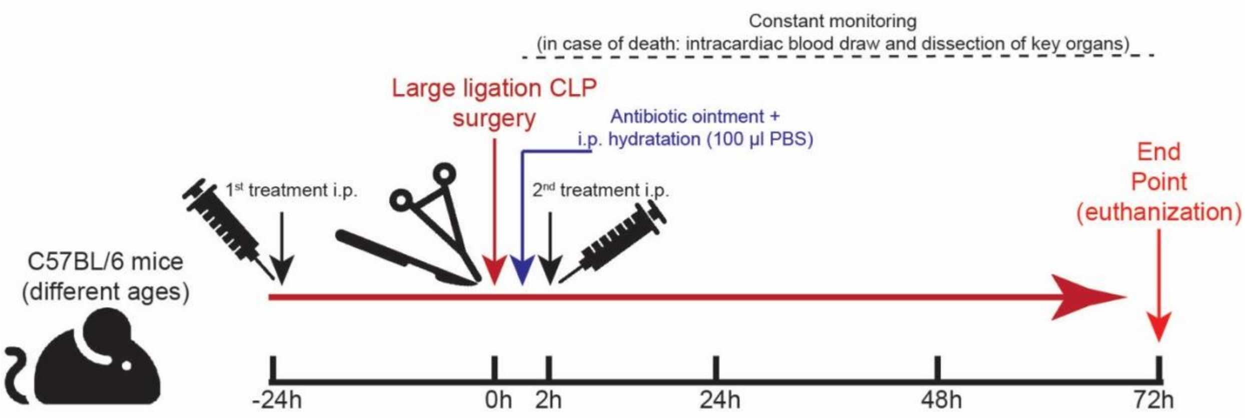

Supplementary Figure 7. Detailed overview of the sepsis CLP anti-miR-93-5p experiment. Male and female C57BL/6 mice of different ages were treated intraperitoneally, 24-hours before CLP induced-sepsis, with scramble miRNA or anti-miR-93-5p. Two hours after the induction of sepsis the treatment was repeated. After surgery, antibiotic ointment was applied at the incision site, and mice were hydrated with 100 µl PBS i.p. Mice were monitored for 72 hours after CLP, at the end of the 72h surviving mice were euthanized. At the time of death, blood was drawn, and key organs were harvested during the postmortem dissection.

Supplementary Figure 8

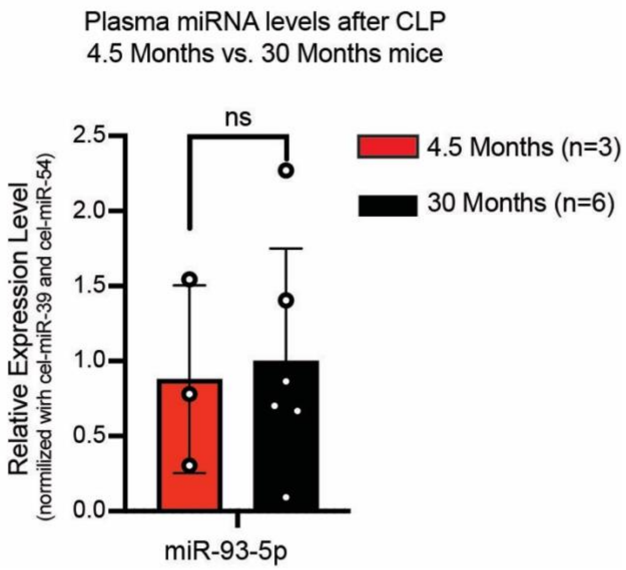

Supplementary Figure 8. Plasma levels in mice of different ages. Plasma levels of miR-93-5p were measured in 4.5-month-old (n = 3) and 30-month-old (n = 6) sepsis mice (CLP). Data are presented as means ± s.d. (Student's t test; n.s., not significant).

Supplementary Figure 9

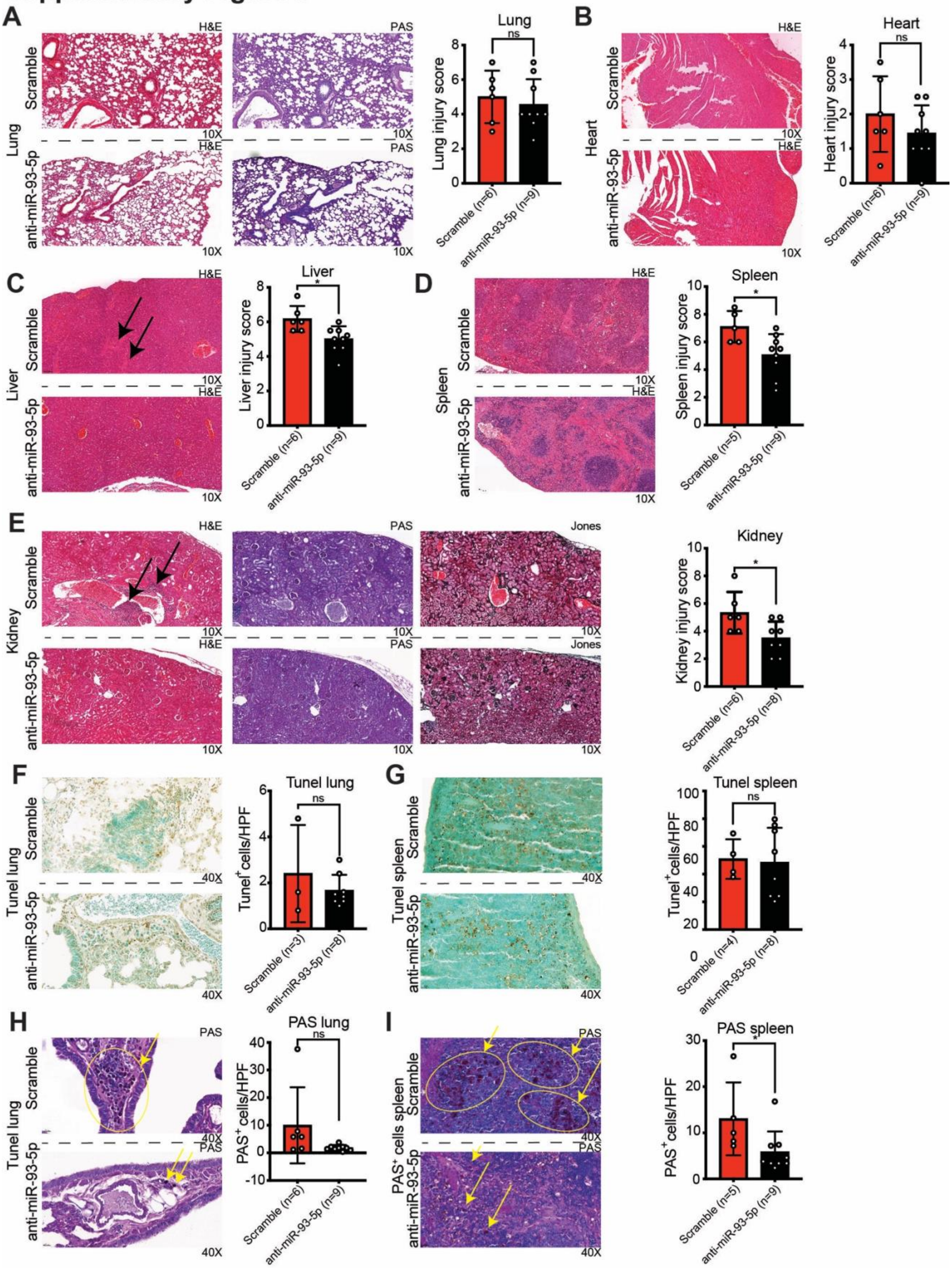

Supplementary Figure 9. Tissue damage score in 16-month-old sepsis mice treated with scramble miRNA compared to sepsis mice treated with anti-miR-93-5p. (**A**) Lung injury score in CLP sepsis mice treated with scramble miRNA (n = 6) or with anti-miR-93-5p (n = 9). (**B**) Heart injury score in CLP sepsis mice treated with scramble miRNA (n = 6) or with anti-miR-93-5p (n = 9). (**C**) Liver injury score in CLP sepsis mice treated with scramble miRNA (n = 6) or with anti-miR-93-5p (n = 9). Black arrows represent necrotic hepatic parenchyma. (**D**) Spleen injury score in CLP sepsis mice treated with scramble miRNA (n = 6) or with anti-miR-93-5p (n = 9). (**E**) Kidney injury score in CLP sepsis mice treated with scramble miRNA (n = 6) or with anti-miR-93-5p (n = 8). Black arrows represent acute inflammatory infiltrates. (**F**) TUNEL-positive cells/high-power field in the lungs of CLP sepsis mice treated with scramble miRNA (n = 3) or with anti-miR-93-5p (n = 8). (**G**) TUNEL-positive cells/high power field in the spleens of CLP sepsis mice treated with scramble miRNA (n = 4) or with anti-miR-93-5p (n = 8). (**H**) PAS-positive cells/high power field in the lungs of CLP sepsis mice treated with scramble miRNA (n = 6) or with anti-miR-93-5p (n = 9). Yellow arrows represent PAS-positive immune cells, and yellow circles represent accumulations of PAS-positive immune cells. (**I**) PAS-positive cells/high-power field in the spleens of CLP sepsis mice treated with scramble miRNA (n = 6) or with anti-miR-93-5p (n = 8). Yellow arrows represent PAS-positive immune cells, and yellow circles represent accumulations of PAS-positive immune cells. Data are presented as means ± s.d. (Student's t test; n.s., not significant; $^*P < 0.05$).

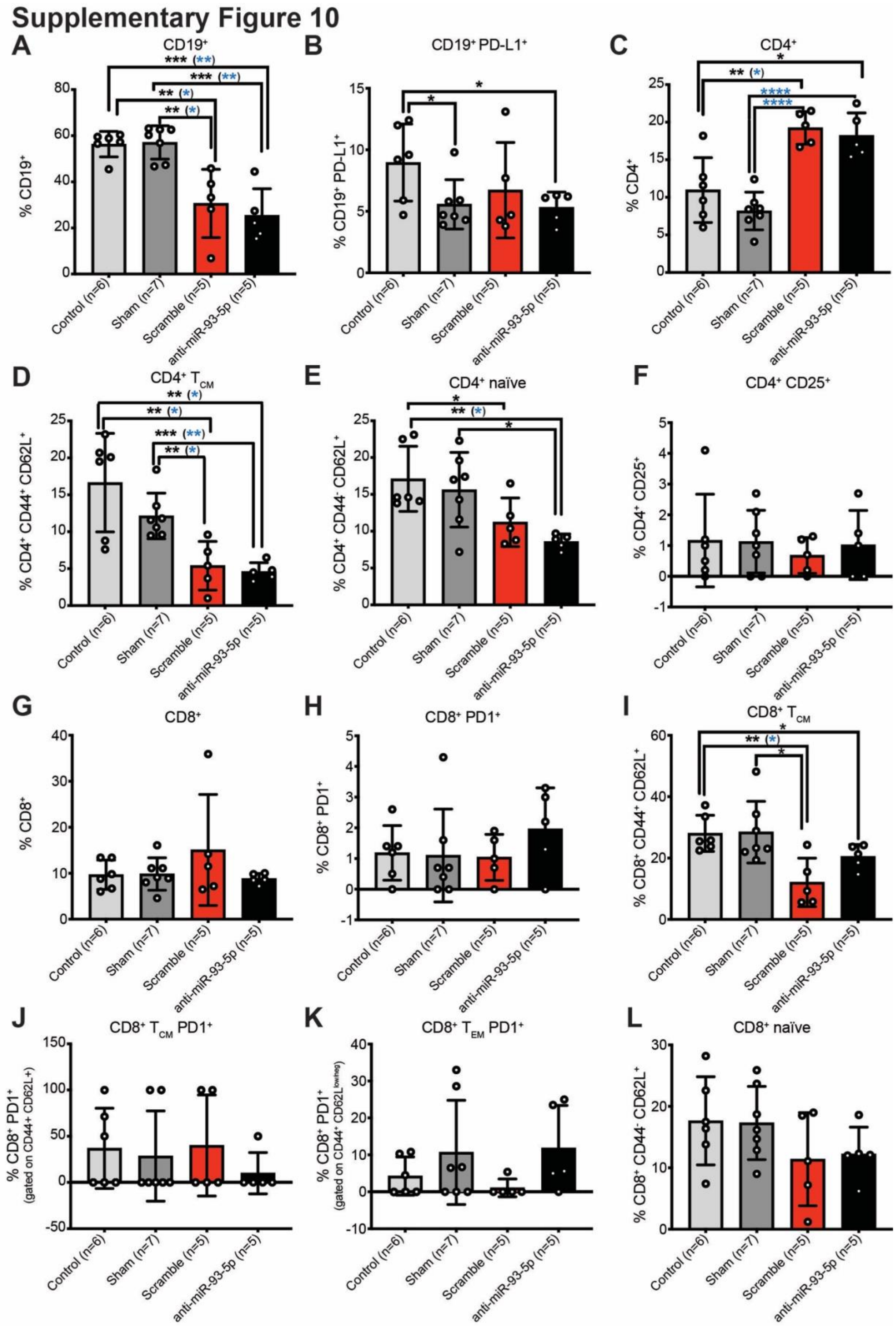
Supplementary Figure 10
A
CD19+
% CD19+
*** (**)
*** (**)
** (*)
** (*)
B
CD19+ PD-L1+
% CD19+ PD-L1+
*
*
C
CD4+
% CD4+
*
** (*)
****
****
D
CD4+ TCM
% CD4+ CD44+ CD62L+
** (*)
** (*)
*** (**)
** (*)
E
CD4+ naïve
% CD4+ CD44- CD62L+
*
** (*)
*
F
CD4+ CD25+
% CD4+ CD25+
G
CD8+
% CD8+
H
CD8+ PD1+
% CD8+ PD1+
I
CD8+ TCM
% CD8+ CD44+ CD62L+
*
** (*)
*
J
CD8+ TCM PD1+
% CD8+ PD1+ (gated on CD44+ CD62L+)
K
CD8+ TEM PD1+
% CD8+ PD1+ (gated on CD44+ CD62L low/neg)
L
CD8+ naïve
% CD8+ CD44- CD62L+
Control (n=6)
Sham (n=7)
Scramble (n=5)
anti-miR-93-5p (n=5)

Supplementary Figure 10. Effect of anti-miR-93-5p therapy on the lymphoid lineage. (A) The percentage of $CD19^+$ and **(B)** $CD19^+$ PD-L1 B cells in control, sham-operated, CLP-induced sepsis treated with scramble miRNA, and CLP-induced sepsis treated with anti-miR-93-5p mice. **(C)** The percentage of T-helper cells ($CD4^+$) and of different subtypes of T-helper: **(D)** $CD4^+$ central memory cells ($CD4^+$ T_{CM}), and **(E)** $CD4^+$ naïve cells in the four experimental groups. **(F)** The percentage of T_{reg} cells ($CD4^+$ $CD25^+$) in control, sham-operated, CLP-induced sepsis treated with scramble miRNA, and CLP-induced sepsis treated with anti-miR-93-5p mice. **(G)** The percentage of T-cytotoxic cells ($CD8^+$), and of **(H)** $CD8^+$ $PD1^+$ cells. **(I)** The percentage of $CD8^+$ central memory ($CD8^+$ T_{CM}) cells. **(J)** The percentage of $CD8^+$ T_{CM} expressing PD1. **(K)** The percentage of $CD8^+$ effector memory cells ($CD8^+$ T_{EM}) expressing $PD1^+$, and **(L)** $CD8^+$ naïve cells in the four experimental groups. Data are presented as means ± s.d. (Student's t test; *P < 0.05; **P < 0.01; ***P < 0.001; ****P < 0.0001; P values that are significant after adjustment for multiple testing using the false discovery rate (FDR) are marked blue).

Supplementary Figure 11

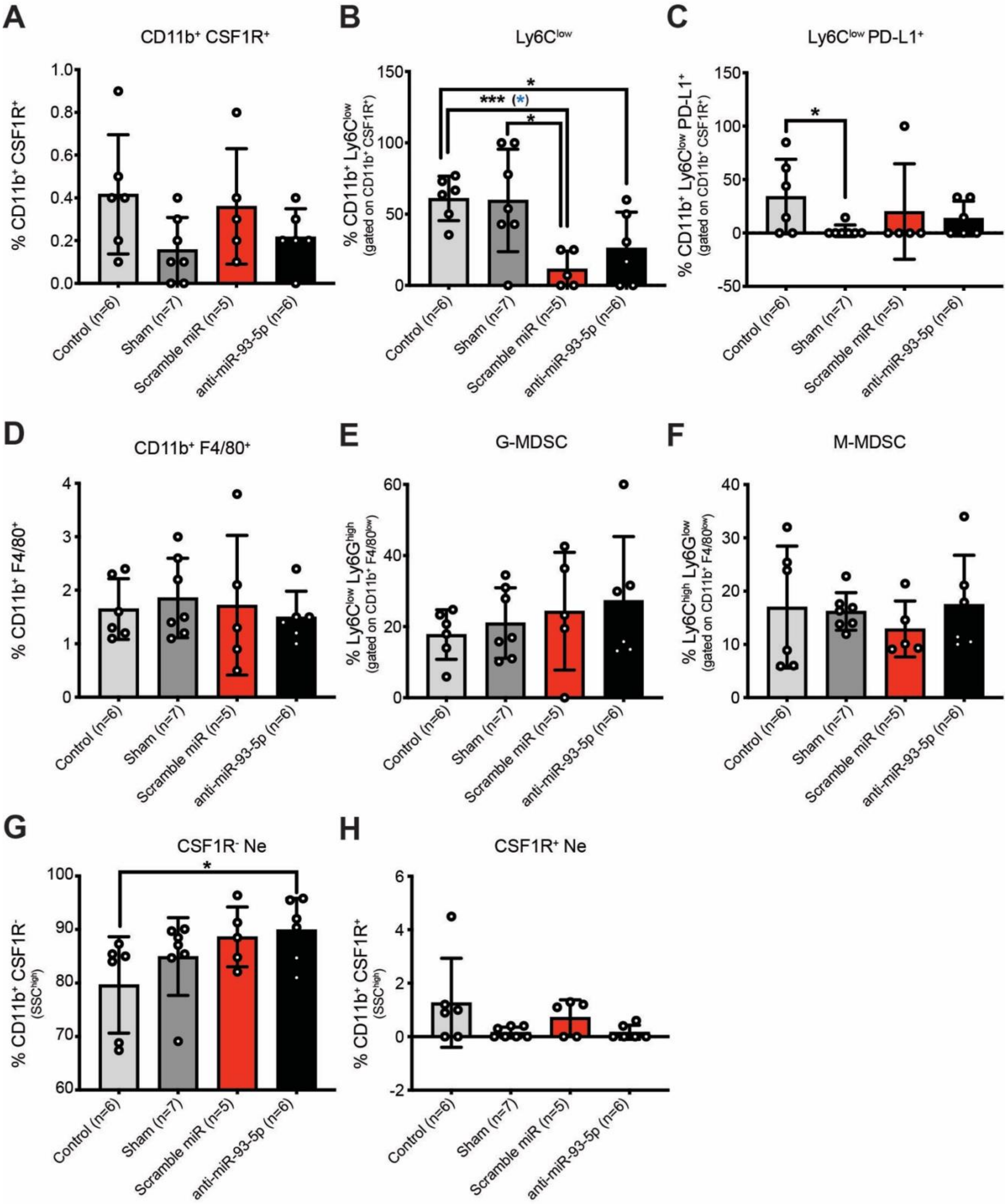

Supplementary Figure 11. Effect of anti-miR-93-5p therapy on the myeloid lineage. (A) The percentage of the whole pool of monocytes ($CD11b^+$ $CSF1R^+$) in control, sham-operated, CLP-induced sepsis treated with scramble miRNA and CLP-induced sepsis treated with anti-miR-93-5p mice. **(B)** The percentage of $CD11b^+$ LyC^{low} cells to the whole pool of $CD11b^+$ $CSF1R^+$ monocytes and of **(C)** LyC^{low} $PD\text{-}L1^+$ monocytes in the four experimental arms. **(D)** The percent of macrophages ($CD11b^+$ $F4/80^+$) in control, sham-operated, CLP-induced sepsis treated with scramble miRNA, and CLP-induced sepsis treated with anti-miR-93-5p mice. **(E)** The percent of granulocytic myeloid-derived suppressor cells (G-MDSC) and **(F)** monocytic myeloid-derived suppressor cells (M-MDSC) in control, sham-operated, CLP-induced sepsis treated with scramble miRNA, and CLP-induced sepsis treated with anti-miR-93-5p mice. The percentage of **(G)** $CSF1R^-$ neutrophils ($CSF1R^-$ Ne) and **(H)** $CSF1R^+$ neutrophils ($CSF1R^+$ Ne) gated to SSC^{high} $CD45^+$ in the four experimental groups. Data are presented as means ± s.d. (Student's t test; *$P < 0.05$; ***$P < 0.001$; P values that are significant after adjustment for multiple testing using the false discovery rate (FDR) are marked blue).

Supplementary Figure 12

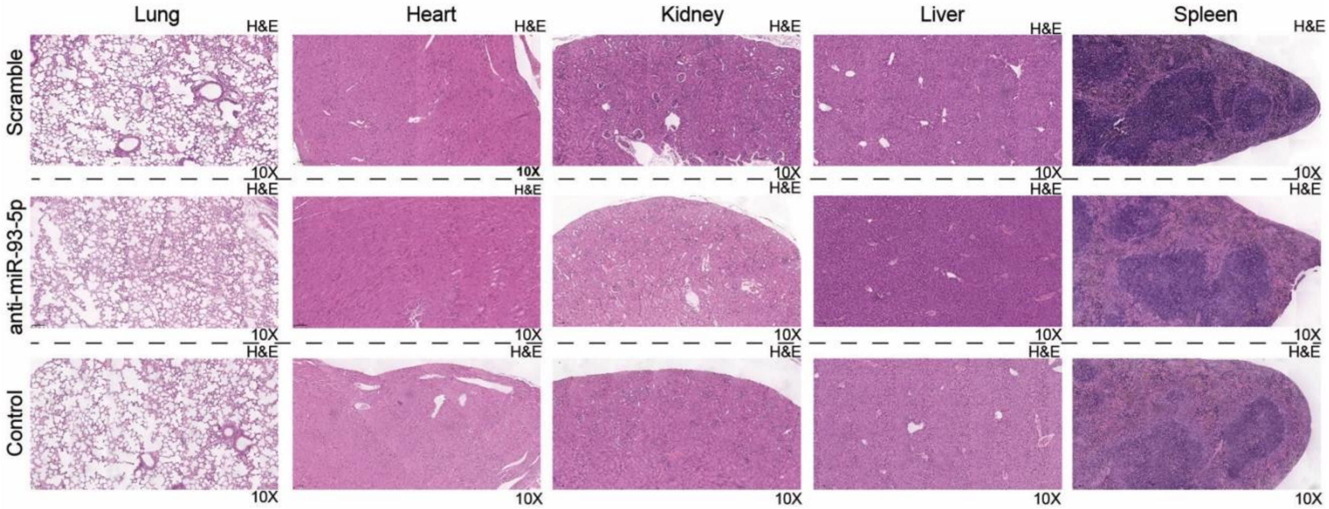

Supplementary Figure 12. Effect of *in vivo* miRNA treatments on organ histopathology in CLP sepsis mice treated with scramble miRNA, CLP sepsis mice treated with anti-miR-93-5p compared to control mice. Representative organ (lung, heart, kidney, liver, and spleen) histology section stained with hematoxylin and eosin (H&E) from mice untreated (Control) and treated with scramble miRNA and anti-miR-93-5p at a concentration of 200µg/kg of body weight. The treatment groups received two therapeutic doses, the first one 24 hours before the induction of sepsis and a second one two hours after the induction of sepsis. All mice were sacrificed 24 hours after sepsis induction. Images were taken at 100X magnification (10X objective lens).

Supplementary Figure 13

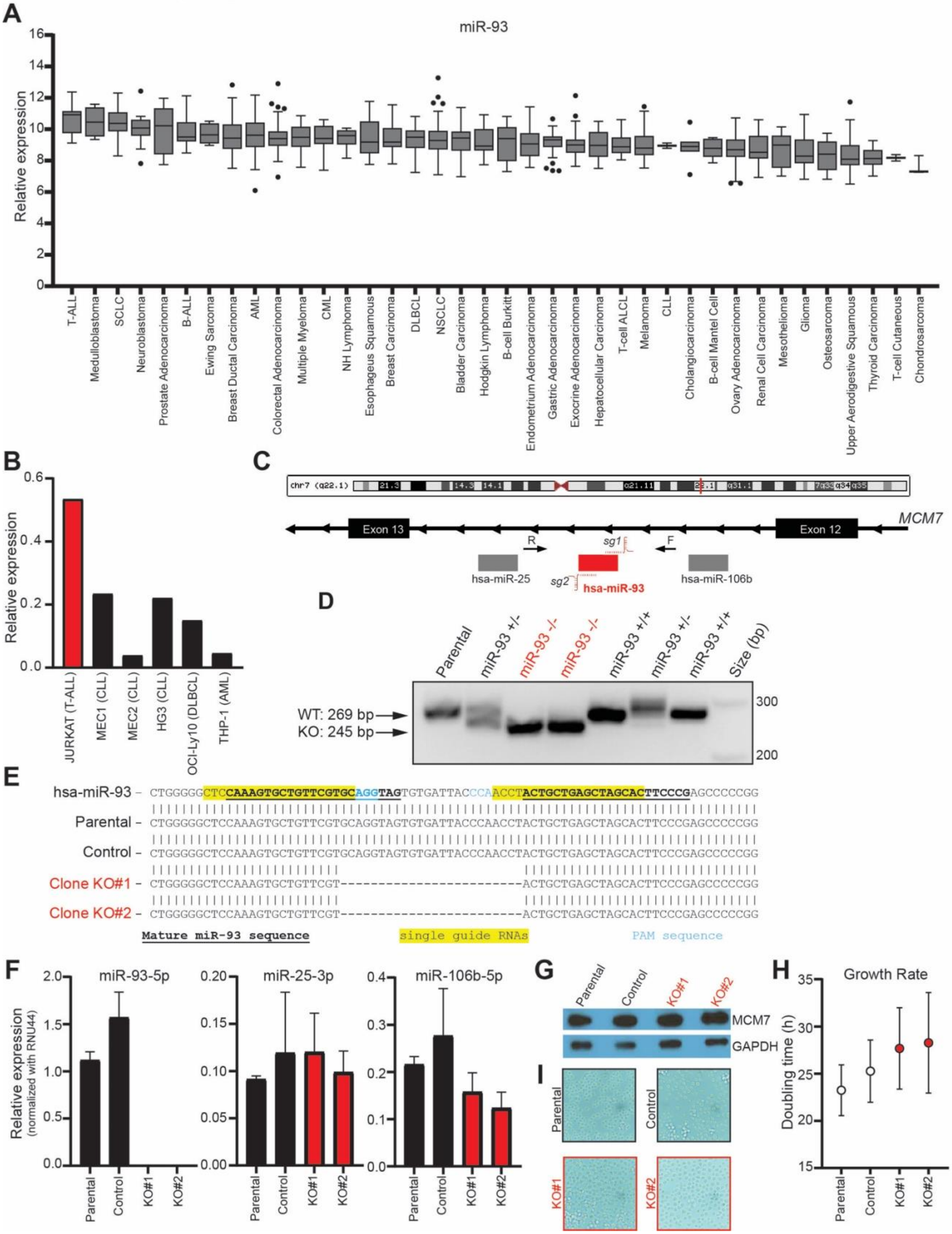

Supplementary Figure 13. Validation of miR-93 knockout in JURKAT cells. **(A)** Expression levels of miR-93 as reported by the Cancer Cell Line Encyclopedia (CCLE). Among blood cancers, acute lymphoblastic leukemia (T/B-ALLs), acute myeloid leukemia (AML), chronic myeloid leukemia (CML), and non-Hodgkin lymphoma show the highest level of miR-93. **(B)** RT-qPCR measurement of miR-93-5p levels in 6 different cell lines, including T-ALL (JURKAT), CLL (Mec1, MEC2, HG3), DLBCL (OciLy10), and AML (THP-1). Data are presented as means. **(C)** Schematic of the genomic location of hsa-miR-93. MiR-93 is located intronic of *minichromosome maintenance complex component 7* (MCM7) as a miRNA cluster, flanked by miR-25 and miR-106b. Two single guide RNAs (sg1/2) were designed to flank the loop region of miR-93. Forward (F) and reverse (R) primers used for validation by PCR and sequencing are indicated. (**D**) PCR analysis of multiple CRISPR/Cas9-created clones showing unaffected, heterozygous, and homozygous knockouts. Clones shown in red were used for further analysis. (**E**) The sequence of hsa-miR-93 is indicated with mature -3p and -5p sequences (bold, underscored), the location of sgRNAs (yellow), and PAM sequences (blue). Sequencing results of JURKAT parental cells and three clones, one unaffected control and two homozygous knockouts are shown. A 24-bp deletion encompassing the miRNA loop region is observed in both KO clones. (**F**) The more prevalently expressed arm of each miRNA was assessed, showing absent expression of miR-93-5p in both KO clones while neighboring miR-25-3p and miR-106b-5p remain unaffected. (**G**) The knockout does not affect the levels of the miRNA host gene MCM7 at the protein level, as shown by Western blot. **(H)** Growth rate in parental, control, and KO#1 and KO#2 clones. Shown is the mean ± s.d. of the doubling time calculated across 12 continuous culture days. (**I**) Morphology of parental, control, and KO#1 and KO#2 after miR-93 knockout.

Supplementary Figure 14

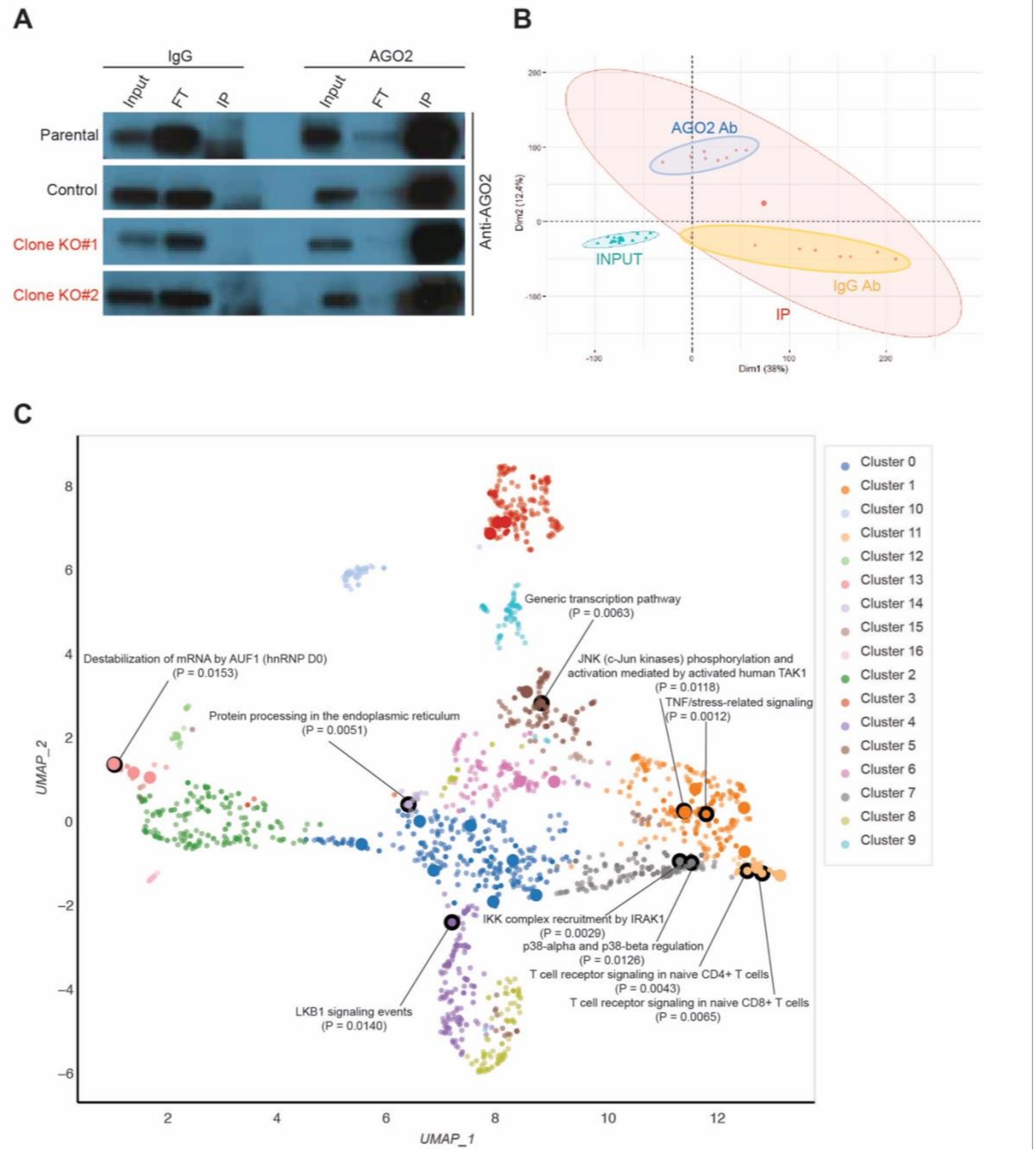

Supplementary Figure 14. Technical validation of AGO2-immunoprecipitation. (**A**) Western blot analysis of all isolated fractions during the IP procedure, including input, flow-through (FT), and immunoprecipitated (IP). IP was performed on four JURKAT clones (i.e., parental, CRISPR/Cas Control, miR-93 KO#1, and KO#2) in duplicate using either anti-AGO2 or nonspecific IgG antibody control. A representative blot is shown. (**B**) Principal component analysis shows the separation of input (turquois) and IP (red) fractions in the first dimension and the separation of anti-AGO2 (blue) and anti-IgG (yellow) mediated pulldown in the second dimension. Dimension three separates the two miR-93 knockouts from CRISPR control and parental samples (data not shown). (**C**) Uniform Manifold Approximation and Projection (UMAP) dimensionality reduction (two dimensions) of the miR-93-5p AGO2-Immunoprecipitation Targetome Pathways. The top 10 pathways are outlined in bold circles.

Supplementary Figure 15

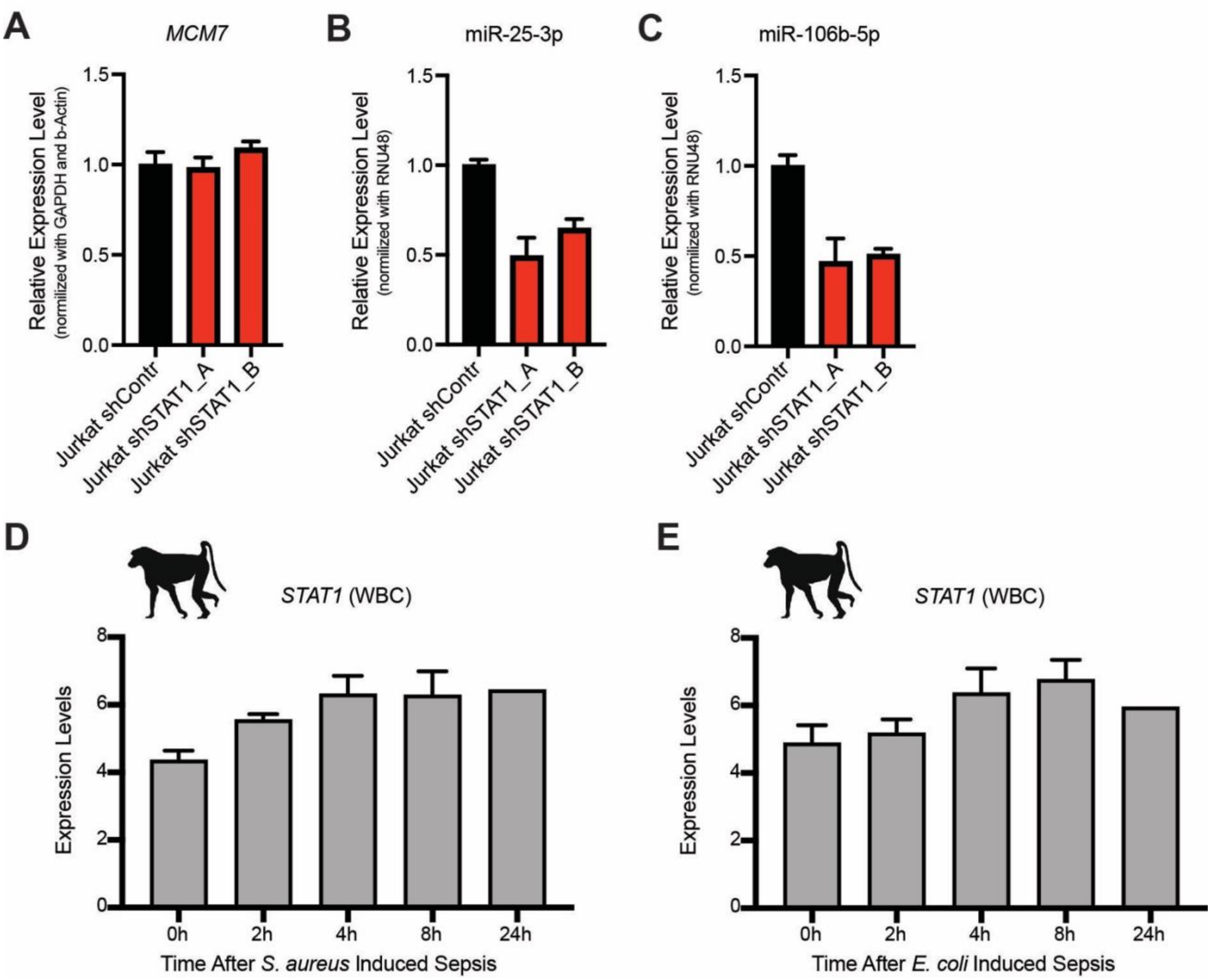

Supplementary Figure 15. Upstream regulation of miR-93-5p. (**A**) *MCM7* mRNA expression levels in JURKAT shControl and in shSTAT1_A and shSTAT1_B. (**B**) miR-25-3p and (**C**) miR-106b-5p expression level in JURKAT shControl and in shSTAT1_A and shSTAT1_B. (**D**) *STAT1* levels in WBC upon sepsis induction in the *S. aureus* baboon model. (**E**) *STAT1* levels in WBC upon sepsis induction in the *E. coli* baboon model.

Supplementary Figure 16

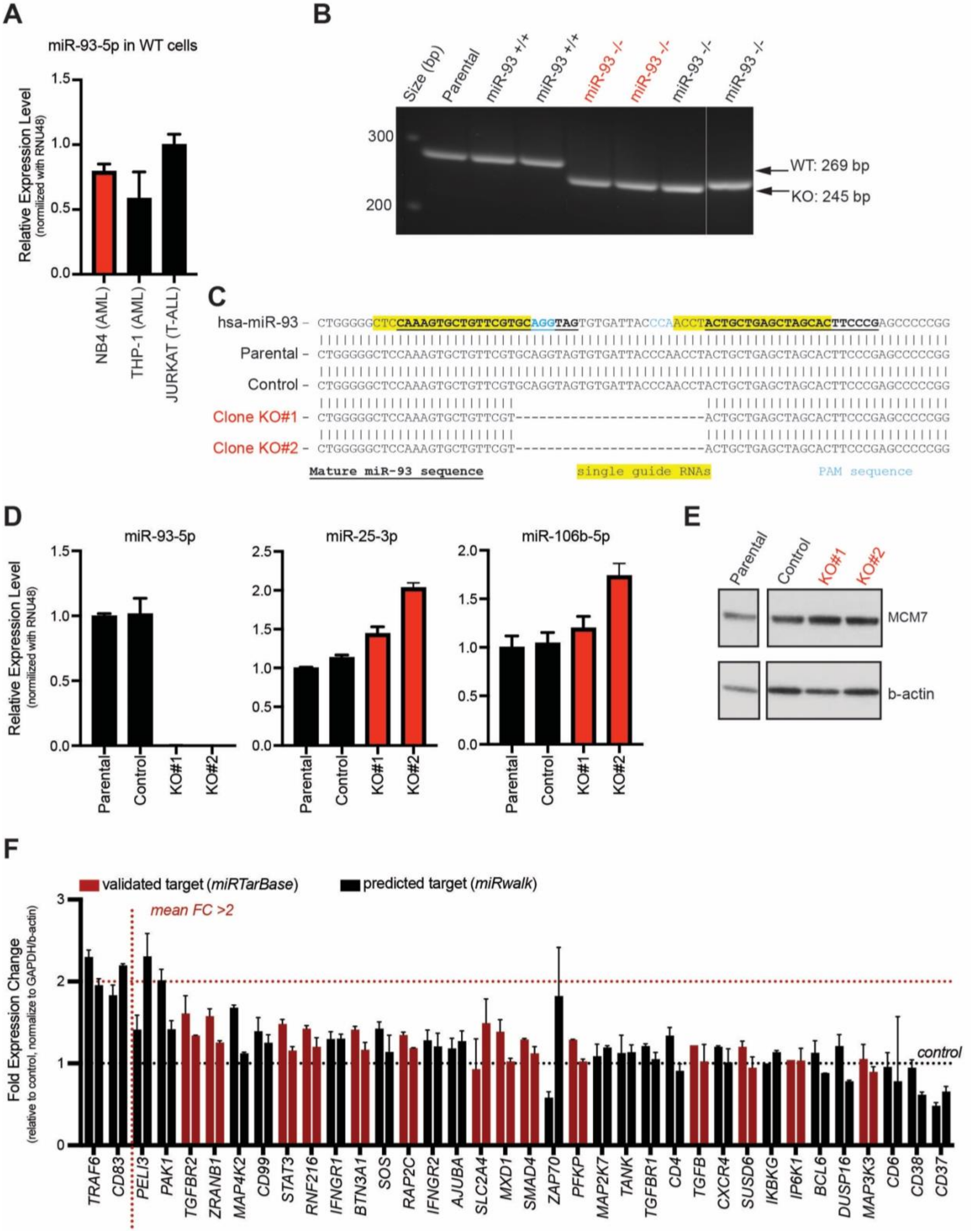

Supplementary Figure 16. miR-93-5p target genes in the myeloid lineage (NB4). (**A**) RT-qPCR measurement of miR-93-5p levels in JURKAT and two cell lines of the myeloid lineage, NB4 and THP-1. (**B**) PCR analysis of multiple CRISPR/Cas9-created clones showing unaffected and homozygous knockouts. Clones shown in red were used for further analysis. (**C**) The sequence of hsa-miR-93 indicated with mature -3p and -5p sequences (bold, underscored), the location of sgRNAs (yellow), and PAM sequences (blue). Sequencing results of NB4 parental cells and three clones – one unaffected control and two homozygous knockouts are shown. A 24-bp deletion encompassing the miRNA loop region is observed in both KO clones. (**D**) The more prevalently expressed arm of each miRNA was assessed, showing almost absent expression of miR-93-5p in both KO clones while neighboring miR-25-3p and miR-106b-5p show a slight up-regulation. (**E**) The knockout does not affect the levels of the miRNA host gene MCM7 at the protein level, as shown by Western blot. **(F**) 36 expressed genes with immune functions that were identified in the JURKAT AGO2-IP and are validated (red) or predicted (black) targets of miR-93-5p were assessed in NB4 miR-93 knockouts compared to controls by RT-qPCR. Bars indicate fold changes in gene expression in the KO#1 and KO#2 samples relative to the control (control = 1).

Supplementary Figure 17

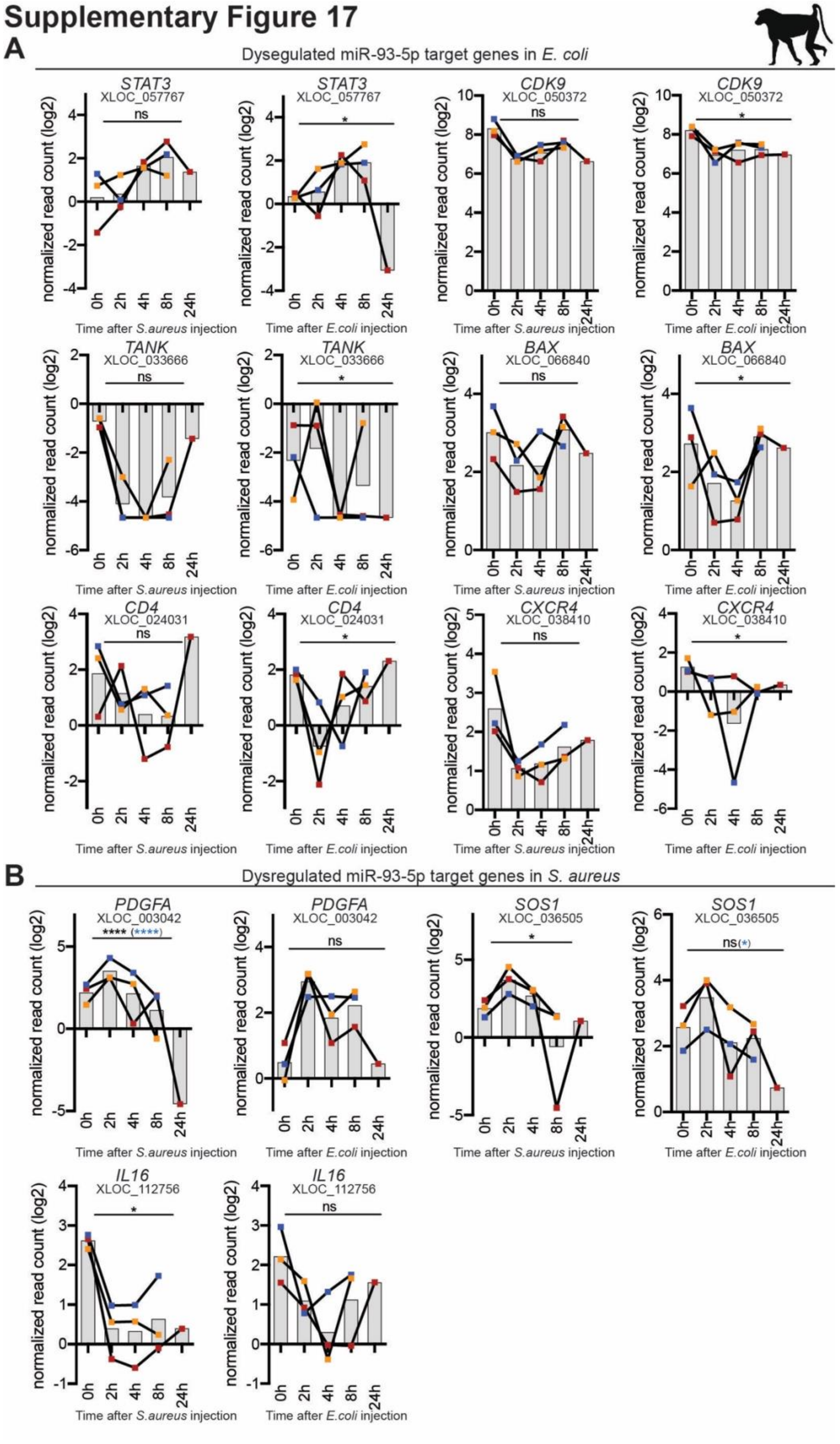

Supplementary Figure 17. Downregulated genes upon sepsis induction in either the *E. coli*-mediated baboon model or the *S. aureus*-mediated baboon model. (**A**) Downregulation of *STAT3*, *CDK9*, *TANK, BAX*, *CD4*, and *CXCR4* upon sepsis induction in the *E. coli*-mediated baboon model. * Q-value < 0.05. (**B**) Downregulation of *PDGFA, SOS1,* and *IL16* upon sepsis induction in the *S. aureus* baboon model. Moderate t test; * Q-value < 0.05, **** Q < 0.0001. Results were also adjusted to the subject effects using the linear mixed effects model (LMM), and significant P values after adjustment for multiple testing are marked in blue color.

Supplementary Table S1. Characteristics of cohort #1 sepsis patients (n = 23) included in the study.

ID	Tissue	Age	Cancer type	Treatment
**13**	whole blood	65	Acute Myeloid Leukemia	Targeted
**20**	whole blood	72	Acute Myeloid Leukemia	Cytotoxic
**21**	whole blood	46	Chronic Myeloid Leukemia	Targeted; Cytotoxic
**23**	whole blood	62	Multiple Myeloma	Cytotoxic
**26**	whole blood	32	Leukemia	Targeted; Cytotoxic
**42**	whole blood	57	Non-Small Cell Lung Cancer	Radiotherapy; Immunotherapy
**43**	whole blood	62	Appendix Adenocarcinoma	Surgery; HIPEC
**44**	whole blood	74	Multiple Myeloma	Targeted
**47**	whole blood	51	Acute Myeloid Leukemia	Targeted; Cytotoxic
**48**	whole blood	75	Melanoma	Radiotherapy
**50**	whole blood	26	B Cell Acute Lymphoblastic Leukemia	Targeted; Cytotoxic
**52**	whole blood	67	Multiple Myeloma	Cytotoxic
**58**	whole blood	63	Acute Myeloid Leukemia	Cytotoxic
**59**	whole blood	60	Prostate Cancer	None
**61**	whole blood	42	Chronic myeloid Leukemia	None
**65**	whole blood	51	Squamous cell carcinoma of base of tongue	None
**71**	whole blood	88	Diffuse Large B Cell Lymphoma	None
**80**	whole blood	52	Acute Myeloid Leukemia	None
**98**	whole blood	51	Non-small Cell Lung Carcinoma	None
**120**	whole blood	82	Lymphoma	None
**168**	whole blood	59	Acute Myeloid Leukemia	None
**173**	whole blood	47	Acute Myeloid Leukemia	Targeted
**175**	whole blood	69	Myelodysplastic syndrome	None

Supplementary Table S2. Characteristics of cohort #2 sepsis patients (n = 63) included in the study.

Patient ID	PBMC	Plasma	Age	APACHE II Final	SOFA	MEDS	SOPED	CRP	ANC	ALC	CCI
**SC3**	yes	yes	45	16	5	5	12	N/A	N/A	N/A	8
**SC4**	yes	yes	68	14	1	6	16	11.28	16.38	1.01	9
**SC5**	yes	yes	30	7	5	3	0	N/A	1.25	0.29	2
**SC6**	yes	yes	55	11	3	3	5	145.12	8.35	0.16	6
**SC7**	yes	yes	72	19	5	8	4	N/A	0.32	0.44	4
**SC8**	yes	yes	50	9	4	0	0	N/A	1.76	1.68	2
**SC9**	yes	yes	67	17	0	5	9	N/A	17.93	1.33	8
**SC10**	yes	yes	47	2	3	0	5	N/A	9.96	1.73	10
**SC11**	yes	yes	64	16	5	2	21	N/A	17.38	2.49	7
**SC12**	yes	N/A	71	11	6	5	4	N/A	93.5	2.8	2
**SC13**	yes	yes	55	8	2	3	0	168.41	5.25	0.07	2
**SC14**	yes	yes	67	14	8	6	4	N/A	N/A	N/A	5
SC15	N/A	N/A	57	12	8	9	0	192.53	N/A	N/A	4
**SC16**	yes	yes	58	8	5	3	5	N/A	4	0.12	7
**SC17**	yes	yes	59	9	3	0	0	N/A	2.06	0.68	4
**SC18**	yes	yes	40	7	4	0	0	N/A	6.43	0.15	2
**SC19**	yes	yes	45	7	2	2	9	N/A	7.13	0.55	6
**SC20**	yes	yes	20	3	1	2	9	N/A	4.48	0.96	6
**SC21**	yes	yes	35	6	3	2	16	206.41	8.79	0.19	6
**SC22**	yes	yes	71	14	6	9	4	90.34	N/A	N/A	5
**SC23**	yes	yes	63	10	0	0	5	N/A	21.6	1.35	7
**SC24**	yes	yes	50	11	7	3	0	N/A	N/A	N/A	4
**SC25**	yes	yes	64	11	7	3	5	N/A	18.57	2.01	7
**SC26**	yes	yes	37	13	7	3	7	N/A	0.31	3.43	4
**SC27**	yes	yes	29	6	4	6	7	N/A	1.67	1.44	2
**SC28**	yes	yes	66	9	4	10	9	N/A	10.42	0.69	6
**SC29**	yes	yes	56	9	5	3	5	195.34	0.01	0.33	6
**SC30**	yes	yes	28	11	6	3	0	72.62	N/A	N/A	2
**SC31**	yes	yes	56	6	4	0	7	N/A	14.92	2.74	2
**SC32**	yes	yes	57	8	1	3	5	9.76	5.65	0.06	12
**SC33**	yes	yes	81	8	5	3	11	115.11	11.34	0.53	3
**SC34**	yes	yes	70	11	5	3	11	N/A	9.88	4.92	4
**SC35**	yes	yes	27	7	8	3	0	N/A	0	0.5	2
**SC36**	yes	yes	62	10	7	3	0	N/A	N/A	N/A	2

**SC37**	yes	yes	44	9	3	0	7	N/A	13.54	1.12	13
**SC38**	yes	yes	70	15	6	8	4	54.62	0.5	0.1	4
**SC39**	yes	yes	22	12	7	3	0	N/A	N/A	N/A	2
**SC40**	N/A	yes	29	9	7	3	0	285	N/A	N/A	2
**SC41**	yes	yes	74	21	6	5	13	N/A	21.74	0.46	6
**SC43**	yes	yes	48	4	3	0	0	141.51	6.9	1.09	6
**SC44**	N/A	yes	44	8	6	3	5	172.51	0.4	0.42	5
SC45	N/A	N/A	57	13	9	6	0	N/A	0.01	0.91	2
**SC46**	yes	yes	28	13	7	3	0	90.55	N/A	N/A	6
**SC47**	yes	yes	64	7	2	11	16	187.31	27.5	0.86	5
**SC48**	N/A	yes	63	10	7	6	7	199.11	0	0.3	6
**SC49**	yes	yes	50	5	4	3	5	7.39	3.1	0.12	2
**SC50**	yes	yes	64	16	7	3	0	N/A	0.55	0.02	6
**SC51**	yes	yes	60	9	1	0	5	45.27	1.85	0.16	2
**SC52**	yes	yes	41	5	3	0	5	N/A	14.19	1.74	2
**SC53**	yes	yes	62	12	9	10	11	N/A	1.23	2.09	2
**SC54**	yes	yes	22	18	6	8	14	269.23	19.63	15.95	7
**SC55**	yes	yes	75	10	4	10	22	N/A	2.44	2.91	2
**SC56**	yes	yes	29	7	2	3	0	N/A	2.19	0.63	2
**SC57**	yes	N/A	24	6	1	5	11	85.34	8.85	1.16	2
**SC58**	yes	yes	46	11	6	3	0	N/A	N/A	N/A	6
**SC59**	yes	yes	49	11	9	5	5	172.59	N/A	N/A	6
**SC60**	yes	yes	53	4	4	3	5	N/A	12.21	0.1	6
**SC61**	yes	yes	43	9	4	8	16	N/A	24.78	1.75	2
**SC62**	yes	yes	74	18	7	8	8	114.08	1.2	0.11	9
**SC63**	yes	yes	71	11	6	11	20	85.16	1.56	0.36	4
**SC64**	yes	yes	61	10	6	3	0	N/A	2.59	0.62	5
**SC65**	yes	yes	51	21	10	6	7	234.26	2.45	0.11	2
**SC66**	yes	yes	33	10	6	8	11	N/A	0	3.18	N/A

Supplementary Table S3. 583 putative miR-93 target genes

Probe	Gene Symbol	Primary identifyer	*FC IP WT vs IP miR-93 KO*	P-val_IP	FDR P-val_IP	*FC IP Control vs IP miR-93 KO*	P-val_IP	FDR P-val_IP	miR-93-5p target (predicted, miRwalk)	miR-93-5p target (confirmed, miRTarBase)
**TC1400007033**	**FAM179B**	NM_001308120	*13.85*	<0.0001	<0.0001	*3.2*	0.0079	0.0592	0	
**TC1400010195**	**WARS**	NM_004184	*7.82*	0.0022	0.0179				1	
**TC2200008409**	**ZMAT5**	NM_001003692	*7.76*	0.0007	0.0075				1	
**TC0300012170**	**GOLGB1**	NM_001256486	*7.68*	0.0022	0.0178				1	
**TC0X00010982**	**CDR1**	NM_004065	*5.76*	<0.0001	0.001				0	
**TC0X00010465**	**NUP62CL**	NM_017681	*5.72*	<0.0001	0.0001	*7.65*	<0.0001	<0.0001	0	
**TC0800010285**	**SLC20A2**	NM_001257180	*5.64*	<0.0001	0.0001				1	
**TC1400006529**	**ANG; RNASE4**	NM_001097577	*5.04*	0.0009	0.0089	*3.86*	0.0042	0.0378	0	
**TC0200015002**	**CHN1**	NM_001025201	*5.01*	0.0003	0.0041	*2.55*	0.0235	0.1256	1	
**TC0800012396**	**FBXO16**	NM_001258211	*4.85*	0.0018	0.0152				0	
**TC0100011873**	**RNF187**	NM_001010858	*4.73*	0.0047	0.0318				0	
**TC0200015976**	**SP110**	NM_001185015	*4.68*	0.0015	0.0131				1	
**TC0800008145**	**WWP1**	NM_007013	*4.62*	0.0031	0.0234				1	
**TC1600010593**	**CMTM4**	NM_178818	*4.56*	0.0002	0.0031				1	
**TC0700013022**	**ACTR3C**	NM_001164458	*4.54*	0.0044	0.0302				0	
**TC1800008734**	**STARD6**	NM_139171	*4.49*	0.0017	0.0147				0	
**TC1500007545**	**SNX1**	NM_001242933	*4.48*	0.0022	0.0182				1	
**TC2100008536**	**PCBP3**	NM_001130141	*4.45*	0.0001	0.0019	*2.58*	0.0081	0.0601	1	
**TC0500012791**	**KCNMB1**	NM_004137	*4.44*	<0.0001	0.0006				1	MIRT518695
**TC0100013123**	**UBR4**	NM_020765	*4.41*	0.0017	0.0147				1	
**TC0700006737**	**ARL4A**	NM_001037164	*4.36*	0.001	0.01				1	
**TC0200010216**	**ITGAV**	NM_001144999	*4.29*	0.0083	0.048				1	
**TC1900011684**	**BABAM1**	NM_001033549	*4.22*	0.006	0.0377				1	
**TC1100011398**	**MRGPRD**	NM_198923	*4.19*	0.0004	0.0047				1	
**TC1100012303**	**PPP2R1B**	NM_001177562	*4.11*	0.0003	0.0042	*2.22*	0.0276	0.1396	1	
**TC0300006471**	**SETMAR**	NM_001243723	*4.11*	0.0022	0.0182				0	
**TC0100009418**	**RAP1A**	NM_001010935	*4.1*	0.0019	0.0161				1	

**TC1200009881**	**CLEC2B**	NM_005127	*4.08*	0.008	0.0469				1	
**TC0900011453**	**ZBTB6**	NM_006626	*4.05*	0.0004	0.0051				1	MIRT72679
**TC0500013186**	**PTCD2**	NM_001284403	*3.97*	0.0141	0.0703				1	
**TC0100010525**	**SFT2D2**	NM_199344	*3.93*	0.0001	0.0019				1	
**TC0100008190**	**CMPK1**	NM_001136140	*3.91*	0.0046	0.0311				0	
**TC1600008156**	**LRRC36**	NM_001161575	*3.88*	0.0008	0.008				1	
**TC1900011873**	**ZNF433**	NM_001080411	*3.87*	0.0154	0.0746	*-3.24*	0.0329	0.1562	0	
**TC0200014550**	**ORC4**	NM_001190879	*3.85*	<0.0001	0.0014				1	
**TC1200009967**	**DUSP16**	NM_030640	*3.82*	0.0006	0.0061				1	
**TC0600012652**	**FAXC**	NM_032511	*3.75*	0.0122	0.063				1	MIRT615447
**TC0300013336**	**LAMP3**	NM_014398	*3.74*	<0.0001	0.0006	*2.41*	0.0023	0.0237	1	
**TC0900009905**	**CNTFR**	NM_001207011	*3.72*	0.0009	0.0088				1	
**TC0800008241**	**TMEM67**	NM_001142301	*3.69*	0.0023	0.0187				1	MIRT726282
**TC0200013610**	**REV1**	NM_001037872	*3.66*	0.0002	0.0031				1	MIRT296869
**TC0200016471**	**MXD1**	NM_001202513	*3.62*	0.0082	0.0477				1	MIRT437749
**TC1700010721**	**STAT3**	NM_003150	*3.61*	0.0129	0.0656				1	MIRT28178
**TC1100007787**	**CD6**	NM_001254750	*3.59*	0.0001	0.0021				1	
**TC1500008983**	**SLC12A6**	NM_001042494	*3.57*	0.0039	0.0275				1	MIRT782765
**TC1100008392**	**PLEKHB1**	NM_001130033	*3.55*	0.0013	0.0122				0	
**TC0300011032**	**NCKIPSD**	NM_016453	*3.53*	0.001	0.0095	*2.21*	0.0267	0.1367	1	
**TC1200011936**	**ATXN2**	NM_001310121	*3.51*	0.0089	0.0508				1	
**TC1400010765**	**RDH11**	NM_001252650	*3.51*	0.0009	0.0088				1	
**TC0600007303**	**BTN3A1**	NM_001145008	*3.49*	0.0002	0.0027				1	MIRT168279
**TC1000009240**	**EDRF1**	NM_001202438	*3.49*	0.0022	0.0179				1	
**TC0100010365**	**FCRLA**	NM_001184866	*3.46*	0.0006	0.0069				1	
**TC0100008621**	**LEPR; LEPROT**	NM_001003679	*3.45*	0.0216	0.0941				0	
**TC0800012454**	**ZHX1**	NM_001017926	*3.44*	0.0007	0.0072				1	
**TC1700012216**	**LGALS9**	NM_002308	*3.43*	0.0054	0.035	*2.83*	0.0164	0.0968	0	
**TC1900010962**	**MYPOP**	NM_001012643	*3.4*	0.0013	0.0123	*2.61*	0.0091	0.065	0	
**TC0400009855**	**TMEM128**	NM_001297551	*3.4*	0.0025	0.0197				1	
**TC1500007975**	**IREB2**	NM_004136	*3.4*	0.0114	0.0605				1	

**TC1900008172**	**ZNF574**	NM_022752	*3.37*	0.0059	0.0372				1	
**TC1000011501**	**TCTN3**	NM_001143973	*3.35*	0.0011	0.0109	*2.55*	0.0087	0.0631	1	
**TC0700010170**	**RNF216**	NM_207111	*3.34*	0.0166	0.0783				1	MIRT726727
**TC0800011249**	**RNF19A**	NM_001280539	*3.32*	0.0275	0.1105				1	
**TC0900010878**	**FANCC**	NM_000136	*3.31*	0.0065	0.0401				1	
**TC1200012083**	**FBXO21**	NM_015002	*3.31*	0.0076	0.0452				1	MIRT727672
**TC1700010802**	**MPP2**	NM_001278370	*3.29*	0.0119	0.0624				1	
**TC1500007365**	**LIPC**	NM_000236	*3.28*	0.0015	0.0132				0	
**TC0700013429**	**PILRA**	NM_013439	*3.26*	0.0003	0.0033				1	
**TC1700006500**	**TUSC5**	NM_172367	*3.25*	0.0059	0.0373				1	
**TC0600013054**	**SERINC1**	NM_020755	*3.22*	0.0045	0.0306	*2.4*	0.0272	0.1383	1	MIRT686992
**TC1800007829**	**HSBP1L1**	NM_001136180	*3.21*	0.0019	0.0158				1	
**TC1900010801**	**CNFN**	NM_032488	*3.2*	0.0012	0.0114				1	
**TC2000010022**	**ZNFX1**	NM_021035	*3.19*	0.0007	0.0072	*2.96*	0.0013	0.0156	1	MIRT48879
**TC0100016260**	**ALDH9A1**	NM_000696	*3.18*	0.0077	0.0455				1	MIRT728362
**TC0700010621**	**FKBP14**	NM_017946	*3.18*	0.0036	0.0257				1	MIRT688229
**TC0X00011406**	**CXorf40B**	NM_001013845	*3.18*	0.0137	0.0687				1	
**TC0600009843**	**PLEKHG1**	NM_001029884	*3.17*	0.0112	0.0597				1	
**TC1100010077**	**SBF2**	NM_030962	*3.17*	0.0002	0.0029				1	
**TC0300014048**	**NPHP3-ACAD11**	NR_037804	*3.16*	0.0007	0.0072				0	
**TC0X00006614**	**MSL3**	NM_001193270	*3.16*	0.0213	0.0931				1	
**TC1900007900**	**LOC101927572; AC002116.7; CLIP3**	NM_001290056	*3.16*	0.003	0.0227				0	
**TC0100017446**	**SUSD4**	NM_001037175	*3.15*	0.0018	0.0157				1	
**TC1000006858**	**HSPA14**	NM_001278205	*3.15*	0.0091	0.0513				1	
**TC1400009351**	**SIX1**	NM_005982	*3.13*	0.002	0.0168				1	
**TC2200009274**	**APOBEC3G**	NM_021822	*3.11*	0.0028	0.0217	*2.12*	0.0384	0.1725	1	
**TC0600009808**	**PCMT1**	NM_001252049	*3.09*	0.0008	0.0086				1	
**TC1200007461**	**PCED1B**	NM_001281429	*3.07*	0.0001	0.002				1	
**TC0300008142**	**TBC1D23**	NM_001199198	*3.06*	0.0113	0.0601				1	
**TC1100007943**	**PLCB3**	NM_000932	*3.06*	0.0014	0.0129				1	

**TC1700010328**	**UTP6**	NM_018428	*3.06*	0.0008	0.0084				0	
**TC0600007092**	**FAM8A1**	NM_016255	*3.05*	0.0001	0.0016				0	
**TC0X00009650**	**WDR45; PRAF2**	NM_001029896	*3.05*	0.0043	0.0295				0	
**TC0300008559**	**PARP15**	NM_001113523	*3.04*	0.0226	0.0968				1	
**TC1300007565**	**SLAIN1**	NM_001040153	*3.04*	0.0092	0.052				1	
**TC1400007691**	**DLST**	NM_001244883	*3.04*	0.0135	0.0677				1	
**TC0200016768**	**PECR**	NM_018441	*3.02*	0.0181	0.0831				1	
**TC1700008820**	**C17orf80**	NM_001100621	*3.02*	0.0046	0.0311				1	
**TC0900011021**	**TEX10**	NM_001161584	*3.01*	0.0181	0.0833				1	
**TC1600010347**	**RPGRIP1L**	NM_001127897	*3.01*	0.0037	0.0263				1	
**TC1500009355**	**SHC4**	NM_203349	*3*	0.0121	0.0628				1	
**TC0100009929**	**SETDB1**	NM_001145415	*2.99*	0.0141	0.0702				1	
**TC1600009986**	**LOC730183; RP11-146F11.1; hitema**	NM_001256932	*2.98*	0.0004	0.0046				0	
**TC0900009846**	**B4GALT1**	NM_001497	*2.97*	0.0003	0.0041	*2.97*	0.0003	0.0055	1	
**TC0900007488**	**PIP5K1B**	NM_001278253	*2.97*	0.0015	0.0131				1	
**TC0700009394**	**AGK**	NM_018238	*2.96*	0.0167	0.0785				1	
**TC1100013129**	**CTSD**	NM_001909	*2.95*	0.0044	0.03	*2.07*	0.0459	0.1923	0	
**TC1500007874**	**C15orf39**	NM_015492	*2.93*	0.0017	0.0148	*2.44*	0.0072	0.0558	1	
**TC0900010922**	**ZNF510**	NM_014930	*2.93*	0.0123	0.0633				1	
**TC1400009617**	**DPF3**	NM_001280542	*2.93*	0.0256	0.1056				1	
**TC0300009179**	**MED12L**	NM_053002	*2.92*	0.0149	0.0728	*2.45*	0.0387	0.1731	1	MIRT28147
**TC0100015082**	**DPH5**	NM_001077394	*2.9*	0.0041	0.0287	*3.89*	0.0005	0.0071	1	
**TC0400012967**	**MGARP**	NM_032623	*2.9*	0.0093	0.0521				0	
**TC0300007013**	**ARPP21**	NM_001025068	*2.89*	0.0096	0.0533				1	
**TC0400008213**	**RAP1GDS1**	NM_001100426	*2.87*	0.0131	0.0663				1	
**TC1900007570**	**ZNF492**	NM_020855	*2.84*	0.0012	0.0116	*2.34*	0.0065	0.0519	1	
**TC0700011318**	**ERV3-1; ZNF117**	NM_001007253	*2.84*	0.0125	0.0644				0	
**TC2000009673**	**SYCP2**	NM_014258	*2.83*	0.0167	0.0786	*-2.78*	0.0181	0.1037	1	
**TC1200012730**	**PXMP2**	NM_018663	*2.83*	0.0407	0.1444				0	

**TC0300013751**	**BDH1**	NM_004051	*2.82*	0.0296	0.1162	*2.89*	0.0261	0.1348	1	
**TC1900011724**	**ZNF540; ZNF571-AS1**	NM_001172225	*2.82*	0.026	0.1067				0	
**TC1100009611**	**JAM3**	NM_001205329	*2.81*	0.0294	0.1156				1	
**TC1200007061**	**CMAS**	NM_018686	*2.8*	0.0032	0.0236	*2.39*	0.0106	0.0726	0	
**TC1900008533**	**CD37**	NM_001040031	*2.8*	0.0029	0.0221				1	
**TC1400008764**	**GZMB**	NM_004131	*2.79*	0.0003	0.0035	*1.77*	0.0271	0.1378	1	
**TC0100007481**	**PIGV**	NM_001202554	*2.79*	0.0071	0.043				1	
**TC0200011276**	**FAM132B**	NM_001291832	*2.78*	0.0072	0.0433				0	
**TC0300007387**	**RNF123**	NM_022064	*2.78*	0.0078	0.0458				1	
**TC2100008424**	**SLC19A1**	NM_001205206	*2.77*	0.0194	0.0875				1	MIRT48799
**TC0900011252**	**FKBP15**	NM_015258	*2.76*	0.035	0.1306				1	
**TC1300008926**	**KPNA3**	NM_002267	*2.76*	0.0107	0.0578				1	
**TC1700008844**	**TTYH2**	NM_032646	*2.76*	0.0014	0.0131				1	
**TC1100007400**	**TSPAN18**	NM_130783	*2.75*	0.0047	0.0316				1	
**TC0300011154**	**POC1A**	NM_001161580	*2.74*	0.0338	0.1275	*4.26*	0.0034	0.0329	0	
**TC0300007206**	**TCAIM**	NM_001029839	*2.72*	0.0007	0.0078				1	
**TC1400009002**	**SEC23A**	NM_006364	*2.72*	0.0031	0.0231				0	
**TC2200007894**	**CECR1**	NM_001282225	*2.71*	0.0106	0.0572				0	
**TC1600009412**	**TXNDC11**	NM_001303447	*2.69*	0.0175	0.0814	*2.26*	0.0457	0.192	1	
**TC1200012745**	**C1RL**	NM_001297640	*2.69*	0.0429	0.1493				1	
**TC0600007196**	**MRS2**	NM_001286264	*2.68*	0.0042	0.0293				0	
**TC1000010367**	**ZNF25**	NM_145011	*2.68*	0.0201	0.0898				0	
**TC0100018081**	**ZNF670; ZNF695; ZNF670-ZNF695**	NM_001204220	*2.67*	0.0023	0.0184				0	
**TC1000011679**	**CUEDC2**	NM_024040	*2.66*	0.0442	0.1523				1	
**TC1200012808**	**R3HDM2**	NM_014925	*2.65*	0.0108	0.0584				1	
**TC1600008193**	**PRMT7**	NM_001184824	*2.65*	0.0102	0.056				1	
**TC0100016290**	**TADA1**	NM_053053	*2.64*	0.0171	0.0798	*2.4*	0.0298	0.1472	1	
**TC1800008338**	**KCTD1**	NM_001136205	*2.64*	0.0094	0.0526				0	
**TC1100010551**	**TRAF6**	NM_004620	*2.63*	0.0277	0.1109	*2.94*	0.0149	0.091	1	

**TC0Y00006476**	**CD99**	NM_001122898_2	*2.63*	0.0096	0.0534				1	
**TC1900007945**	**ZNF529-AS1**	NR_110703	*2.63*	0.0064	0.0396				0	
**TC2200009257**	**TCN2**	NM_000355	*2.63*	0.0008	0.0079				1	
**TC1200012657**	**CAND1**	NM_018448	*2.62*	0.0152	0.0739				1	
**TC1700006719**	**RNASEK; C17orf49; RNASEK-C17orf49**	NM_001004333	*2.62*	0.016	0.0763				0	
**TC0800012132**	**ZC3H3**	NM_015117	*2.61*	0.0242	0.1014	*3.17*	0.0079	0.0592	1	
**TC0900011793**	**MED27**	NM_001253881	*2.61*	0.0103	0.0561	*2.25*	0.0272	0.1382	1	
**TC1200007954**	**USP15; MIR6125**	NM_001252078	*2.6*	0.0438	0.1516				0	
**TC2000007945**	**C20orf197**	NM_001302813	*2.6*	0.0016	0.0141				0	
**TC0500006816**	**FAM105A**	NM_019018	*2.59*	0.0101	0.0555				1	
**TC0600010609**	**PXDC1**	NM_183373	*2.59*	0.0222	0.0957				1	
**TC2200007150**	**TIMP3**	NM_000362	*2.55*	0.0016	0.014	*-1.88*	0.0246	0.1297	0	
**TC1900010886**	**ZNF235**	NM_004234	*2.55*	0.0031	0.0232				0	
**TC0500013351**	**FNIP1**	NM_001008738	*2.54*	0.0135	0.068	*2.54*	0.0137	0.086	1	
**TC0300008919**	**SLC35G2**	NM_001097599	*2.54*	0.0121	0.0628	*2.36*	0.0196	0.11	1	
**TC1400008637**	**SALL2**	NM_001291446	*2.54*	0.0052	0.0343				1	
**TC1800008679**	**MBD1**	NM_001204136	*2.54*	0.0028	0.0215				1	
**TC1700009669**	**ALOX12B**	NM_001139	*2.52*	0.0095	0.0527				1	
**TC0600014045**	**PDCD2**	NM_001199461	*2.51*	0.0068	0.0414	*2.36*	0.0107	0.0732	1	
**TC1900011743**	**TMEM91**	NM_001042595	*2.51*	0.0087	0.0495				0	
**TC0800012026**	**SLC45A4**	NM_001080431	*2.5*	0.0154	0.0745	*2.46*	0.0169	0.099	1	
**TC0200013531**	**KANSL3**	NM_001115016	*2.5*	0.0042	0.0293				1	
**TC0700010581**	**HIBADH**	NM_152740	*2.49*	0.0201	0.0898	*2.56*	0.0171	0.0996	1	
**TC0400011815**	**MFSD8**	NM_152778	*2.49*	0.012	0.0624				1	MIRT54637
**TC0700010321**	**ETV1**	NM_001163147	*2.49*	0.0066	0.0404				1	
**TC1900011305**	**ZNF83**	NM_001105549	*2.49*	0.0031	0.0231				0	
**TC0200009700**	**GALNT13**	NM_001301627	*2.48*	0.0293	0.1154				1	
**TC0200012307**	**SOS1**	NM_005633	*2.48*	0.01	0.0549				1	
**TC0200014189**	**POLR2D**	NM_004805	*2.48*	0.0019	0.0159				0	

**TC1100011014**	**SLC15A3**	NM_016582	*2.48*	0.0022	0.018				1	
**TC1700010019**	**MFAP4**	NM_001198695	*2.48*	0.0415	0.1464				0	
**TC1200010562**	**COL2A1**	NM_001844	*2.47*	0.0033	0.024				1	
**TC1700006946**	**HS3ST3B1**	NM_006041	*2.47*	0.009	0.0511				1	
**TC2000010010**	**BLCAP**	NM_001167820	*2.47*	0.0184	0.0845				1	
**TC0600011235**	**HIST1H2AM; HIST1H3J**	NM_003514	*2.46*	0.0052	0.034				0	
**TC0600013458**	**EPM2A**	NM_001018041	*2.45*	0.0267	0.1084	*2.45*	0.0269	0.1373	1	
**TC0X00006477**	**CD99**	NM_001122898	*2.45*	0.0162	0.0771				1	
**TC1100006656**	**OR52K2**	NM_001005172	*2.45*	0.0165	0.0779				1	
**TC1600007723**	**GPT2**	NM_001142466	*2.45*	0.0153	0.0742				1	
**TC0300013471**	**BCL6**	NM_001130845	*2.44*	0.0343	0.129	*2.53*	0.0286	0.1427	1	
**TC0400006433**	**ZNF595**	NM_001286052	*2.44*	0.0052	0.034				0	
**TC1000008385**	**STAMBPL1**	NM_020799	*2.44*	0.0314	0.1212				1	
**TC1500007379**	**FAM63B**	NM_001040450	*2.44*	0.0212	0.0929				0	
**TC0200011362**	**RNPEPL1**	NM_018226	*2.43*	0.0117	0.0614	*3*	0.0025	0.0254	1	
**TC0200007401**	**DYNC2LI1**	NM_001193464	*2.43*	0.0383	0.1389				1	
**TC0500010580**	**PRKAA1**	NM_006251	*2.43*	0.0254	0.1048				1	
**TC0700013594**	**TRIM4**	NM_033017	*2.43*	0.0275	0.1106				1	
**TC0X00007134**	**USP11**	NM_004651	*2.43*	0.0391	0.1409				0	
**TC1700009613**	**ASGR2**	NM_001181	*2.43*	0.0058	0.0369				1	
**TC0400009892**	**CRMP1**	NM_001014809	*2.41*	0.0111	0.0595				1	
**TC0600007006**	**RNF182**	NM_001165032	*2.41*	0.0067	0.041				1	
**TC0700010504**	**OSBPL3**	NM_015550	*2.41*	0.0427	0.1491				1	
**TC1000008668**	**SFXN3**	NM_030971	*2.41*	0.0212	0.0929				1	
**TC1100011192**	**ATG2A**	NM_015104	*2.41*	0.0136	0.0683				1	MIRT728285
**TC1200008942**	**PLBD2**	NM_001159727	*2.41*	0.0246	0.1029				0	
**TC0100012529**	**PEX10**	NM_002617	*2.38*	0.0057	0.0364	*-2.32*	0.0069	0.0542	0	
**TC1900008750**	**ZNF761; TPM3P9**	NM_001008401	*2.38*	0.0351	0.131				0	
**TC0100015786**	**POGZ**	NM_001194937	*2.37*	0.0186	0.0848				1	
**TC1100012165**	**CASP4**	NM_001225	*2.36*	0.023	0.098	*2.76*	0.0083	0.0612	1	

**TC1100012722**	**CDON**	NM_001243597	*2.36*	0.024	0.1007	*2.32*	0.0267	0.1367	1	
**TC0100008139**	**MAST2**	NM_015112	*2.36*	0.0049	0.0328				1	
**TC0300008550**	**CSTA**	NM_005213	*2.36*	0.0318	0.1221				0	
**TC0400008119**	**TIGD2**	NM_145715	*2.36*	0.0175	0.0812				1	
**TC0500010759**	**CCNO**	NM_021147	*2.36*	0.0093	0.0522				0	
**TC1700012100**	**CSNK1D**	NM_001893	*2.36*	0.0086	0.0491				1	
**TC1700006735**	**SLC2A4**	NM_001042	*2.35*	0.0072	0.0432	*1.86*	0.0423	0.1829	1	MIRT5385
**TC0700009606**	**ZNF282**	NM_001303481	*2.35*	0.0382	0.1388				1	
**TC1500010184**	**BCL2A1**	NM_001114735	*2.35*	0.023	0.0981				1	
**TC0400006951**	**CD38**	NM_001775	*2.34*	0.0128	0.0655				1	
**TC1300008511**	**KATNAL1**	NM_001014380	*2.34*	0.0076	0.0451				1	MIRT27967
**TC0200008556**	**ZAP70**	NM_001079	*2.33*	0.0185	0.0845				1	
**TC0700009093**	**SMKR1**	NM_001195243	*2.33*	0.006	0.0378				1	
**TC0X00007744**	**SH3BGRL**	NM_003022	*2.33*	0.0008	0.0079				1	
**TC1000008447**	**TNKS2**	NM_025235	*2.33*	0.0382	0.1388				1	MIRT57385
**TC2100008487**	**TPTE**	NM_001290224	*2.33*	0.0103	0.0561				1	
**TC1100006456**	**PKP3**	NM_001303029	*2.32*	0.0025	0.0195				1	
**TC1700007603**	**RASL10B**	NM_033315	*2.32*	0.0264	0.1078				1	
**TC1700007769**	**LRRC3C**	NM_001195545	*2.32*	0.0073	0.044				0	
**TC0200014509**	**GTDC1**	NM_001006636	*2.31*	0.0268	0.1088				0	
**TC0X00010401**	**NXF3**	NM_022052	*2.31*	0.0209	0.092				0	
**TC1000007061**	**GPR158**	NM_020752	*2.31*	0.0016	0.0143				1	
**TC1100008333**	**LRTOMT**	NM_001145307	*2.29*	0.0125	0.0643				1	
**TC1500010400**	**PEX11A**	NM_001271572	*2.29*	0.0207	0.0915				1	
**TC0900009461**	**PLGRKT**	NM_018465	*2.28*	0.0037	0.0266				0	
**TC0200010523**	**CD28**	NM_001243077	*2.27*	0.0132	0.0668				1	MIRT684931
**TC0600013341**	**GVQW2**	NM_001242740	*2.27*	0.0127	0.065				0	
**TC0700008878**	**CAPZA2**	NM_006136	*2.27*	0.0356	0.1323				0	
**TC1000012391**	**TUBGCP2**	NM_001256617	*2.27*	0.0211	0.0927				1	
**TC0300014086**	**ATP13A4**	NM_032279	*2.26*	0.006	0.0375				1	
**TC0500013296**	**C1QTNF3-AMACR**	NR_037951	*2.26*	0.0165	0.0779				0	

**TC1000010699**	**IPMK**	NM_152230	*2.26*	0.0233	0.099				1	
**TC1500009804**	**DENND4A**	NM_001144823	*2.26*	0.022	0.0953				1	
**TC0500011602**	**GIN1**	NM_017676	*2.24*	0.043	0.1495	*2.38*	0.0307	0.1498	1	
**TC0900012141**	**GCNT1**	NM_001097633	*2.24*	0.0336	0.127				1	
**TC1100007951**	**CCDC88B**	NM_032251	*2.24*	0.0132	0.0666				1	
**TC1600010648**	**ENKD1**	NM_032140	*2.24*	0.0288	0.1139				0	
**TC1800007859**	**TYMSOS**	NM_001012716	*2.24*	0.011	0.0589				0	
**TC0300011077**	**IP6K1**	NM_001006115	*2.23*	0.0029	0.0219	*2.87*	0.0002	0.0037	1	MIRT48829
**TC1100013109**	**GRAMD1B**	NM_001286563	*2.23*	0.0463	0.1576	*2.61*	0.0192	0.1086	1	
**TC0100014459**	**LINC01359**	NR_119383	*2.23*	0.0222	0.0958				0	
**TC0300010933**	**FYCO1**	NM_024513	*2.22*	0.0261	0.1071				1	MIRT2813
**TC0600011197**	**ZNF184**	NM_007149	*2.22*	0.0044	0.03				0	
**TC0200013595**	**MGAT4A**	NM_001160154	*2.21*	0.0401	0.1431				1	
**TC1500010093**	**ETFA**	NM_000126	*2.21*	0.0164	0.0778				0	
**TC1700009681**	**CTC1**	NM_025099	*2.21*	0.0427	0.1491				1	MIRT48774
**TC1900010074**	**GMIP**	NM_001288998	*2.2*	0.0357	0.1324				1	
**TC2200007313**	**TRIOBP; NOL12**	NM_001039141	*2.2*	0.0172	0.0802				0	
**TC0X00008026**	**H2BFM**	NM_001164416	*2.18*	0.0364	0.1342				1	
**TC1100008979**	**CUL5**	NM_003478	*2.18*	0.0059	0.0374				1	
**TC1700007419**	**TAOK1; MIR4523**	NM_020791	*2.18*	0.0221	0.0955				0	
**TC0100006699**	**THAP3**	NM_001195752	*2.17*	0.0272	0.1097	*2.62*	0.0075	0.057	1	
**TC0400012891**	**NELFA; MIR943**	NM_005663	*2.17*	0.0249	0.1036				0	
**TC1900009590**	**ZNF561**	NM_152289	*2.17*	0.0057	0.0363				1	
**TC1900011207**	**JOSD2**	NM_001270639	*2.17*	0.0086	0.0491				1	
**TC2000008123**	**NRSN2-AS1**	NR_109990	*2.17*	0.0173	0.0804				0	
**TC0100009949**	**PSMD4**	NM_002810	*2.16*	0.0327	0.1244	*2.19*	0.03	0.1479	1	MIRT4885
**TC1700007571**	**ZNF830**	NM_052857	*2.16*	0.0206	0.0913				1	
**TC2100007460**	**MCM3AP-AS1**	NR_002776	*2.15*	0.0359	0.133	*2.81*	0.006	0.0493	0	
**TC1700006449**	**C17orf97**	NM_001013672	*2.15*	0.0117	0.0614				0	
**TC2200008495**	**C22orf24**	NM_001302819	*2.15*	0.0391	0.1409				0	

**TC1100006440**	**RIC8A; MIR6743**	NM_001286134	*2.14*	0.0422	0.1479	*3.35*	0.0022	0.0237	0	
**TC0300013896**	**MFSD1**	NM_001167903	*2.14*	0.0169	0.0794	*2.55*	0.0042	0.0379	1	
**TC0200016626**	**MBOAT2**	NM_138799	*2.14*	0.02	0.0896	*2.21*	0.0159	0.095	1	
**TC0100012376**	**ZNF672**	NM_024836	*2.14*	0.0345	0.1295				1	
**TC0600007613**	**HSPA1A; HSPA1B**	NM_005345	*2.14*	0.0143	0.0706				0	
**TC0100015950**	**FAM189B**	NM_001267608	*2.13*	0.0172	0.0803				1	
**TC1100007394**	**CD82**	NM_001024844	*2.13*	0.0115	0.0608				1	
**TC1500009370**	**FAM227B**	NM_152647	*2.13*	0.0276	0.1107				1	
**TC1700012482**	**SIRT7**	NM_016538	*2.13*	0.0355	0.132				1	
**TC2200009164**	**SYCE3**	NM_001123225	*2.13*	0.0376	0.1375				1	
**TC0100009658**	**SRGAP2C**	NM_001271872	*2.12*	0.0193	0.0873				1	
**TC0100009899**	**C1orf54**	NM_001301039	*2.12*	0.0192	0.0869				0	
**TC0900007165**	**TRMT10B**	NM_001286950	*2.12*	0.0186	0.0848				1	
**TC0200016369**	**DTYMK**	NM_001165031	*2.11*	0.0309	0.1199				1	
**TC1100006492**	**PNPLA2**	NM_020376	*2.11*	0.0121	0.0628				0	
**TC0800008279**	**PLEKHF2**	NM_024613	*2.1*	0.0253	0.1047	*2.13*	0.0229	0.1229	1	
**TC0200007297**	**GEMIN6**	NM_024775	*2.1*	0.0323	0.1235				0	
**TC0700012636**	**PODXL**	NM_001018111	*2.1*	0.0401	0.143				1	
**TC1200012655**	**HELB**	NM_033647	*2.1*	0.0309	0.1199				1	
**TC1300008212**	**TUBA3C**	NM_006001	*2.1*	0.027	0.1093				0	
**TC2200007138**	**FBXO7**	NM_001033024	*2.1*	0.0129	0.0658				1	
**TC1100013138**	**RRP8**	NM_015324	*2.09*	0.0275	0.1107	*2.26*	0.0157	0.0941	1	
**TC0300010672**	**UBP1**	NM_001128160	*2.09*	0.0181	0.0831				1	
**TC1600010276**	**SNX20**	NM_001144972	*2.09*	0.0316	0.1215				1	
**TC1700011084**	**PPP1R9B**	NM_032595	*2.09*	0.035	0.1306				0	
**TC1900011282**	**ZNF614**	NM_025040	*2.09*	0.0272	0.1098				0	
**TC1900012023**	**ZNF836**	NM_001102657	*2.09*	0.0425	0.1485				1	
**TC0100009746**	**NUDT17**	NM_001012758	*2.08*	0.0108	0.0582				0	
**TC0200006476**	**SNTG2**	NM_018968	*2.08*	0.0344	0.1291				1	
**TC0X00010836**	**RAP2C**	NM_001271186	*2.08*	0.0425	0.1485				1	MIRT546823

**TC1000008005**	**MCU**	NM_001270679	*2.08*	0.02	0.0896				1	
**TC1000011577**	**PYROXD2; MIR1287**	NM_032709	*2.07*	0.0277	0.1111				0	
**TC1200012737**	**ZNF268**	NM_001165881	*2.07*	0.0332	0.1259				0	
**TC0100012941**	**PRAMEF13**	NM_001291380	*2.06*	0.0364	0.1342				0	
**TC1700011418**	**LIMD2**	NM_030576	*2.06*	0.0259	0.1066				1	
**TC1400009777**	**POMT2**	NM_013382	*2.05*	0.0346	0.1296	*2.52*	0.0081	0.0603	1	
**TC0300011452**	**UBA3**	NM_003968	*2.05*	0.0259	0.1065				1	
**TC0400007542**	**KIT**	NM_000222	*2.05*	0.0413	0.1458				1	
**TC1200011108**	**WIF1**	NM_007191	*2.05*	0.0377	0.1375				1	
**TC1600007952**	**OGFOD1**	NM_018233	*2.05*	0.0272	0.1098				1	
**TC1600008159**	**HSD11B2**	NM_000196	*2.05*	0.0218	0.0946				1	
**TC1700006767**	**WRAP53**	NM_001143990	*2.05*	0.0298	0.1167				1	
**TC0X00009855**	**ZXDA**	NM_007156	*2.04*	0.029	0.1146	*2.25*	0.0142	0.0881	1	
**TC1000011287**	**GRID1**	NM_017551	*2.04*	0.015	0.0731				1	
**TC0100006989**	**DNAJC16**	NM_001287811	*2.03*	0.0085	0.0488				1	
**TC0200007591**	**ERLEC1**	NM_001127397	*2.03*	0.0216	0.0941				0	
**TC0500010639**	**CCL28**	NM_001301873	*2.03*	0.0366	0.1348				1	
**TC0200010796**	**CTDSP1**	NM_001206878	*2.02*	0.0144	0.0711				1	
**TC0300010209**	**TADA3**	NM_001278270	*2.02*	0.0104	0.0567				0	
**TC0600012175**	**KHDRBS2**	NM_152688	*2.02*	0.0349	0.1304				0	
**TC0900011980**	**SEC16A**	NM_001276418	*2.02*	0.026	0.1066				1	MIRT48866
**TC0900012175**	**CDK9**	NM_001261	*2.02*	0.0152	0.0737				1	
**TC1300009919**	**ATP4B**	NM_000705	*2.02*	0.014	0.0696				1	
**TC0300011276**	**DENND6A**	NM_152678	*2.01*	0.0123	0.0635				1	
**TC0500013300**	**C9**	NM_001737	*2.01*	0.0409	0.1447				1	
**TC2200008862**	**CYP2D6**	NM_000106	*2*	0.0073	0.0436	*-1.75*	0.026	0.1347	1	
**TC0100016350**	**CCDC181**	NM_001300968	*2*	0.0418	0.1471				1	
**TC0X00006656**	**RAB9A**	NM_001195328	*2*	0.0387	0.14				1	
**TC1700008331**	**HLF**	NM_002126	*2*	0.0355	0.132				1	
**TC0200009806**	**TANK**	NM_001199135	*1.99*	0.0393	0.1412	*-2.41*	0.0102	0.0704	1	
**TC1300006930**	**COG6**	NM_001145079	*1.99*	0.0338	0.1275				1	

**TC0600008350**	**BAG2**	NM_004282	*1.98*	0.0338	0.1277				0	
**TC1200007622**	**ATF1**	NM_005171	*1.98*	0.0404	0.1437				1	
**TC1900012000**	**TEAD2**	NM_001256658	*1.98*	0.0441	0.152				0	
**TC1700006645**	**PLD2**	NM_001243108	*1.97*	0.044	0.1517	*2.78*	0.0037	0.0346	1	
**TC0700008094**	**ZP3**	NM_001110354	*1.97*	0.0268	0.1088	*2.31*	0.0076	0.0578	0	
**TC1700007913**	**RAMP2**	NM_005854	*1.97*	0.0217	0.0944	*2.04*	0.0161	0.0957	1	
**TC0100008078**	**ARTN**	NM_001136215	*1.97*	0.0217	0.0944				1	
**TC0500009594**	**CDHR2**	NM_001171976	*1.97*	0.0216	0.0941				1	
**TC1900011239**	**CTU1**	NM_145232	*1.97*	0.0181	0.0833				0	
**TC0200013019**	**MCEE**	NM_032601	*1.96*	0.0368	0.1354				0	
**TC1700012370**	**TBC1D28**	NM_001039397	*1.96*	0.0318	0.1221				1	
**TC0100018045**	**SMYD3**	NM_001167740	*1.95*	0.0427	0.1491				1	
**TC0300011378**	**THOC7**	NM_001285387	*1.95*	0.0145	0.0716				0	
**TC1100009827**	**OSBPL5**	NM_001144063	*1.95*	0.0429	0.1494				1	
**TC1400008688**	**C14orf93**	NM_001130706	*1.95*	0.0436	0.151				1	
**TC0100015943**	**DPM3**	NM_018973	*1.94*	0.0133	0.0672				1	
**TC0600011123**	**HIST1H4B**	NM_003544	*1.94*	0.0308	0.1194				1	
**TC0700013508**	**RADIL**	NM_018059	*1.94*	0.0095	0.0528				1	
**TC0400009936**	**GRPEL1**	NM_025196	*1.93*	0.0223	0.096	*2.6*	0.0016	0.0181	1	
**TC0100012950**	**LRRC38**	NM_001010847	*1.93*	0.0293	0.1153				0	
**TC0500009649**	**RMND5B**	NM_001288794	*1.93*	0.0089	0.0508				1	MIRT48892
**TC0900011962**	**NACC2**	NM_144653	*1.93*	0.0394	0.1415				1	MIRT27987
**TC0100014191**	**RAB3B**	NM_002867	*1.91*	0.0176	0.0815				1	
**TC0300007124**	**ZNF621**	NM_001098414	*1.91*	0.0181	0.0831				1	
**TC1900011235**	**KLK12**	NM_019598	*1.91*	0.023	0.0981				0	
**TC0300011182**	**GLT8D1**	NM_001010983	*1.9*	0.0227	0.0971	*1.85*	0.0292	0.1448	1	
**TC0700008095**	**DTX2**	NM_001102595	*1.89*	0.017	0.0797				1	
**TC1000009221**	**ZRANB1**	NM_017580	*1.89*	0.0226	0.0968				1	MIRT126345
**TC1700010191**	**POLDIP2**	NM_001290145	*1.89*	0.0292	0.1152				1	
**TC1900011866**	**RAB3D**	NM_004283	*1.89*	0.0397	0.142				1	
**TC1800008750**	**TCF4**	NM_001083962	*1.88*	0.036	0.1331	*-1.93*	0.03	0.1477	1	MIRT726343

**TC0700009608**	**ZNF212**	NM_012256	*1.88*	0.0161	0.0768	*-1.96*	0.0108	0.0737	1	
**TC1900010528**	**ZNF260**	NM_001012756	*1.88*	0.0232	0.0988				1	
**TC0400011744**	**ANXA5**	NM_001154	*1.87*	0.044	0.1518				0	
**TC0700006931**	**FAM221A**	NM_001127364	*1.87*	0.0264	0.1079				0	
**TC1600011329**	**TBC1D24**	NM_001199107	*1.87*	0.0345	0.1295				1	
**TC0X00008688**	**MAGEA9B; MAGEA9**	NM_001080790	*1.86*	0.022	0.0953				0	
**TC1200012653**	**METTL21B**	NM_015433	*1.86*	0.0335	0.1268				0	
**TC1500010051**	**RPP25**	NM_017793	*1.85*	0.0112	0.0597	*1.68*	0.0302	0.1482	1	
**TC1100013209**	**PAK1**	NM_001128620	*1.85*	0.042	0.1475				1	
**TC1600006448**	**HBQ1**	NM_005331	*1.85*	0.0209	0.092				1	
**TC1700011967**	**EIF4A3**	NM_014740	*1.85*	0.0326	0.1244				1	
**TC0100016968**	**RABIF**	NM_002871	*1.84*	0.0211	0.0926	*1.68*	0.0484	0.1989	1	
**TC1100012906**	**IGSF9B**	NM_001277285	*1.84*	0.017	0.0796				1	
**TC1100009447**	**FOXRED1**	NM_017547	*1.83*	0.0449	0.154				1	
**TC1600010179**	**DNAJA2**	NM_005880	*1.83*	0.0216	0.0941				1	
**TC1700011772**	**UNC13D**	NM_199242	*1.83*	0.042	0.1475				1	
**TC1100009789**	**TH**	NM_000360	*1.82*	0.0284	0.113				1	
**TC1100010089**	**RNF141**	NM_016422	*1.82*	0.0345	0.1295				1	
**TC1200008370**	**C12orf29**	NM_001009894	*1.82*	0.0162	0.077				1	
**TC0900009078**	**CACFD1**	NM_001135775	*1.81*	0.0404	0.1436				1	
**TC1400010595**	**KIAA0391**	NM_001256678	*1.81*	0.0248	0.1033				1	
**TC1600007528**	**ITGAM**	NM_000632	*1.81*	0.0232	0.0986				1	
**TC0400009525**	**ANKRD37**	NM_181726	*1.8*	0.0302	0.1178	*2.48*	0.0016	0.0183	0	
**TC1600010227**	**N4BP1**	NM_153029	*1.8*	0.0288	0.1139				1	MIRT48847
**TC1800006786**	**RNMT**	NM_001308263	*1.8*	0.0279	0.1116				1	
**TC0300014075**	**TRIM59**	NM_173084	*1.79*	0.0374	0.137				1	
**TC0400006812**	**USP17L24; USP17L26; USP17L5; USP17L27; USP17L29; USP17L30**	NM_001242327	*1.79*	0.0439	0.1516				0	
**TC1600007955**	**MT3**	NM_005954	*1.78*	0.0416	0.1467	*-1.96*	0.0193	0.1089	1	

**TC0200014414**	**CXCR4**	NM_001008540	*1.78*	0.0278	0.1113				1	
**TC1700009954**	**TOM1L2**	NM_001033551	*1.77*	0.01	0.0549	*1.57*	0.0378	0.1711	1	
**TC0100018521**	**TSTD1**	NM_001113205	*1.77*	0.0127	0.0652				0	
**TC1700009619**	**DLG4**	NM_001128827	*1.77*	0.0234	0.0993				1	
**TC0100009742**	**CD160**	NM_007053	*1.76*	0.0414	0.1462				1	
**TC1400006711**	**DHRS4L1**	NM_001277864	*1.76*	0.0448	0.1537				0	
**TC1900010854**	**ZNF428**	NM_182498	*1.76*	0.0357	0.1324				0	
**TC1100012424**	**SIK3**	NM_001281748	*1.75*	0.0439	0.1516				1	
**TC1100013036**	**PPP2R5B**	NM_006244	*1.75*	0.0266	0.1082				1	
**TC1000008736**	**SUFU**	NM_001178133	*1.74*	0.0416	0.1467	*2.01*	0.0117	0.0774	1	
**TC0700013544**	**PSMA2**	NM_002787	*1.74*	0.0221	0.0956	*1.82*	0.0144	0.089	1	
**TC1900010067**	**PBX4**	NM_025245	*1.74*	0.0164	0.0778				1	
**TC1900011077**	**LMTK3**	NM_001080434	*1.74*	0.0429	0.1494				0	
**TC1900011822**	**ZNF586**	NM_001077426	*1.73*	0.0113	0.06				1	
**TC0100011812**	**ADCK3**	NM_020247	*1.72*	0.0325	0.1241	*1.88*	0.0141	0.0878	0	
**TC1800006889**	**RIOK3**	NM_003831	*1.72*	0.0404	0.1437				1	
**TC0400011535**	**DKK2**	NM_014421	*1.7*	0.0252	0.1043				0	
**TC0200014978**	**OLA1**	NM_001011708	*1.69*	0.0316	0.1215				0	
**TC2200008864**	**CYP2D6**	NM_001025161	*1.68*	0.0499	0.1654				1	
**TC1800009212**	**PARD6G**	NM_032510	*1.62*	0.0423	0.1482				1	
**TC1300007824**	**UBAC2**	NM_001144072	*1.6*	0.0441	0.152				1	
**TC1400010715**	**AJUBA**	NM_001289097	*-2.95*	0.0034	0.025	*2.1*	0.0358	0.1653	1	
**TC1700012369**	**FLCN**	NM_144606				*4.37*	<0.0001	0.0008	1	
**TC0900009855**	**BAG1**	NM_001172415				*4.11*	0.0018	0.0197	1	
**TC0700011518**	**GTF2IRD2; GTF2IRD2B**	NM_001281447				*3.86*	<0.0001	0.002	0	
**TC1900006528**	**NDUFS7**	NM_024407				*3.86*	0.0054	0.046	0	
**TC1600011421**	**VPS4A**	NM_013245				*3.84*	0.0008	0.0113	0	
**TC0500007605**	**TRAPPC13**	NM_001093755				*3.8*	0.0012	0.0149	1	
**TC2200008719**	**CBX6**	NM_001303494				*3.77*	0.0029	0.0291	1	
**TC0300007259**	**CCRL2**	NM_001130910				*3.72*	0.0106	0.0725	1	
**TC2000008436**	**TASP1**	NM_017714				*3.68*	0.0254	0.1322	0	

**TC0700009977**	**PDGFA**	NM_002607				*3.66*	0.0005	0.0078	1	
**TC2200008885**	**POLDIP3**	NM_001278657				*3.45*	0.0127	0.0819	1	
**TC0200007181**	**YIPF4**	NM_032312				*3.38*	0.003	0.0295	0	
**TC1600006653**	**THOC6**	NM_001142350				*3.35*	0.0097	0.0682	0	
**TC1400007558**	**SUSD6**	NM_014734				*3.33*	0.0089	0.0642	1	MIRT138794
**TC1100008651**	**TMEM126B**	NM_001193537				*3.24*	0.0022	0.0231	0	
**TC0900008028**	**MFSD14B**	NM_032558				*3.1*	0.0138	0.0862	0	
**TC1100011234**	**LTBP3**	NM_001130144				*3.06*	0.0013	0.0161	1	
**TC1900011708**	**PDCD2L**	NM_032346				*3.06*	0.0081	0.0601	1	
**TC0300013968**	**CCDC51**	NM_001256964				*3.04*	0.0063	0.0505	1	
**TC1000009677**	**ASB13**	NM_024701				*3.04*	0.0005	0.0075	1	
**TC0300010975**	**KIF9**	NM_001134878				*3.02*	0.0257	0.1336	1	
**TC0100018234**	**TNNI3K**	NM_015978				*3*	0.0277	0.14	1	
**TC1900008691**	**ZNF766; MIR643**	NM_001010851				*2.96*	0.0195	0.1095	0	
**TC0X00010211**	**CHM**	NM_000390				*2.93*	0.0104	0.0718	1	
**TC1300006676**	**RASL11A**	NM_206827				*2.88*	0.001	0.013	0	
**TC0300013938**	**VGLL4**	NM_001128219				*2.83*	0.0031	0.0303	1	
**TC1500009720**	**HERC1**	NM_003922				*2.83*	0.0284	0.1423	1	
**TC0300011083**	**TRAIP**	NM_005879				*2.81*	0.0419	0.1815	1	
**TC1700008566**	**MAP3K3**	NM_002401				*2.79*	0.0053	0.0449	1	MIRT147119
**TC2100008222**	**RIPK4**	NM_020639				*2.79*	0.0071	0.0552	1	
**TC1900011329**	**ZNF415**	NM_001136038				*2.76*	0.0047	0.0414	0	
**TC1400006965**	**GEMIN2**	NM_001009182				*2.75*	0.0048	0.0417	1	
**TC1400010015**	**ITPK1**	NM_001142593				*2.73*	0.0154	0.0932	1	
**TC0X00011083**	**TMEM185A**	NM_001174092				*2.71*	0.0022	0.0237	1	
**TC1600006641**	**FLYWCH2**	NM_001142499				*2.71*	0.0122	0.0795	1	
**TC0800008769**	**TRMT12**	NM_017956				*2.7*	0.0183	0.1041	0	
**TC1700006773**	**TMEM88**	NM_203411				*2.7*	0.0015	0.0176	0	
**TC2000006993**	**SYNDIG1**	NM_024893				*2.7*	0.0281	0.1415	1	
**TC2200008734**	**CBX7**	NM_175709				*2.68*	0.0082	0.061	0	

**TC1700012338**	**P2RX5-TAX1BP3**	NR_037928				*2.67*	0.0014	0.0168	0	
**TC0700012917**	**TPK1**	NM_001042482				*2.63*	0.0164	0.0968	1	MIRT525484
**TC0400010606**	**COMMD8**	NM_017845				*2.61*	0.0086	0.0628	1	
**TC1200008591**	**ACTR6**	NM_022496				*2.59*	0.0375	0.1703	1	
**TC1200009141**	**ACADS**	NM_000017				*2.56*	0.0049	0.0427	1	
**TC1600008328**	**DHODH**	NM_001361				*2.56*	0.0224	0.1212	1	MIRT675839
**TC1900009333**	**STAP2**	NM_001013841				*2.56*	0.0093	0.0661	0	
**TC1500010901**	**ST20**	NM_001100879				*2.54*	0.0003	0.0048	0	
**TC0500008632**	**SLC22A5**	NM_001308122				*2.53*	0.0073	0.0561	1	
**TC1900009917**	**EPS15L1**	NM_001258374				*2.52*	0.0289	0.1438	1	MIRT51497
**TC1900010360**	**ANKRD27**	NM_032139				*2.52*	0.0181	0.1036	1	
**TC0500009488**	**CREBRF**	NM_001168393				*2.51*	0.0325	0.1551	1	MIRT2882
**TC1700006709**	**XAF1**	NM_017523				*2.51*	0.0087	0.0632	1	
**TC0200008261**	**VAMP5**	NM_006634				*2.5*	0.0024	0.025	0	
**TC1700008011**	**DBF4B**	NM_025104				*2.49*	0.025	0.131	0	
**TC0200010927**	**MRPL44**	NM_022915				*2.46*	0.0288	0.1434	1	
**TC1600008971**	**JMJD8**	NM_001005920				*2.46*	0.0271	0.1379	0	
**TC1200009071**	**PEBP1**	NM_002567				*2.43*	0.0211	0.116	1	
**TC0300011223**	**SELK**	NM_021237				*2.41*	0.0055	0.0461	0	
**TC0400012905**	**QDPR**	NM_000320				*2.41*	0.0159	0.0951	1	
**TC0X00009256**	**KLHL15**	NM_030624				*2.41*	0.0036	0.0343	1	MIRT727335
**TC1500008978**	**EMC7**	NM_020154				*2.41*	0.0115	0.0763	0	
**TC0200011040**	**ITM2C**	NM_001012514				*2.4*	0.0104	0.0719	1	
**TC0600014086**	**ZNF391**	NM_001076781				*2.4*	0.016	0.0953	1	
**TC1600011223**	**SNAI3**	NM_178310				*2.4*	0.0041	0.0374	1	
**TC1400008118**	**AK7**	NM_152327				*2.39*	0.0262	0.135	0	
**TC0100018479**	**NOTCH2NL**	NM_203458				*2.38*	0.0263	0.1354	1	
**TC1900006499**	**ARID3A**	NM_005224				*2.37*	0.005	0.0431	1	
**TC0200015087**	**SESTD1**	NM_178123				*2.36*	0.0075	0.0571	1	
**TC1200009111**	**CCDC64**	NM_207311				*2.36*	0.03	0.1479	0	

**TC1400010714**	**HAUS4; MIR4707**	NM_001166269				*2.36*	0.0325	0.1551	0	
**TC1900007415**	**DDX49**	NM_019070				*2.36*	0.0313	0.1517	1	
**TC1500010658**	**VIMP**	NM_018445				*2.35*	0.0189	0.1069	0	
**TC0700013102**	**KMT2C**	NM_170606				*2.34*	0.006	0.049	1	
**TC1400008462**	**C14orf79**	NM_174891				*2.34*	0.021	0.1157	0	
**TC1700006999**	**ADORA2B**	NM_000676				*2.34*	0.0055	0.0464	1	
**TC1900011683**	**HSH2D**	NM_001291274				*2.34*	0.0054	0.0461	0	
**TC1100013020**	**PPP1R32**	NM_001170753				*2.33*	0.0148	0.0906	0	
**TC0X00008881**	**SPRY3**	NM_001304990				*2.32*	0.0126	0.0815	1	
**TC0Y00006855**	**SPRY3**	NM_005840_2				*2.32*	0.0126	0.0815	1	
**TC1400009104**	**RPL36AL**	NM_001001				*2.31*	0.0223	0.1206	0	
**TC1600008991**	**LMF1**	NM_022773				*2.31*	0.0274	0.139	0	
**TC2000008230**	**C20orf27**	NM_001039140				*2.31*	0.0128	0.0823	1	
**TC0600011381**	**FLOT1**	NM_005803				*2.3*	0.0016	0.0183	1	
**TC0900006758**	**DENND4C**	NM_017925				*2.29*	0.0243	0.1286	1	
**TC0100013368**	**PAQR7**	NM_178422				*2.29*	0.0335	0.158	1	
**TC1300009012**	**THSD1**	NM_018676				*2.24*	0.0329	0.1564	0	
**TC2200007982**	**UFD1L**	NM_001035247				*2.24*	0.0072	0.0557	0	
**TC0200016767**	**MREG**	NM_018000				*2.22*	0.0062	0.0502	1	
**TC2000008095**	**TPD52L2**	NM_001243891				*2.21*	0.018	0.1033	1	
**TC1000012423**	**PFKP**	NM_001242339				*2.2*	0.0268	0.1369	1	MIRT56477
**TC0500010169**	**ANKH**	NM_054027				*2.19*	0.018	0.1032	1	MIRT9572
**TC0900011220**	**SUSD1**	NM_001282640				*2.19*	0.0211	0.116	1	
**TC1200007821**	**SUOX**	NM_000456				*2.19*	0.0197	0.1102	0	
**TC1100010903**	**YPEL4**	NM_145008				*2.18*	0.0096	0.0675	1	
**TC0100009040**	**RPAP2**	NM_024813				*2.17*	0.0308	0.15	1	
**TC0100012674**	**GPR153**	NM_207370				*2.17*	0.0206	0.1139	0	
**TC0200010823**	**CDK5R2**	NM_003936				*2.17*	0.0179	0.1029	0	
**TC1200007895**	**OS9**	NM_001017956				*2.17*	0.0387	0.1731	1	
**TC1500007866**	**PPCDC**	NM_001301101				*2.15*	0.009	0.0648	1	
**TC1400008058**	**PPP4R4**	NM_020958				*2.14*	0.0384	0.1725	1	

**TC1200012575**	**DYRK4**	NM_001282285				*2.13*	0.0181	0.1034	1	
**TC0600007854**	**PI16**	NM_001199159				*2.12*	0.0318	0.1535	1	
**TC0X00007716**	**P2RY10**	NM_014499				*2.12*	0.0417	0.1811	1	
**TC1700007704**	**ARHGAP23**	NM_001199417				*2.12*	0.0409	0.1789	1	
**TC0500008941**	**ARHGAP26**	NM_001135608				*2.11*	0.0255	0.1327	1	
**TC1200012666**	**TMEM19**	NM_018279				*2.11*	0.0274	0.1393	1	
**TC1000008758**	**CNNM2**	NM_017649				*2.1*	0.0213	0.1168	1	
**TC1200010699**	**POU6F1**	NM_002702				*2.1*	0.0372	0.1693	1	
**TC1400008722**	**ZFHX2**	NM_033400				*2.1*	0.0052	0.0448	1	
**TC1500010778**	**IL16**	NM_001172128				*2.09*	0.0421	0.1822	1	
**TC1900006864**	**MAP2K7**	NM_001297555				*2.09*	0.0259	0.134	1	
**TC1900008505**	**BAX**	NM_001291428				*2.09*	0.0076	0.0577	0	
**TC0200013765**	**UXS1**	NM_001253875				*2.08*	0.0151	0.0918	0	
**TC0200012299**	**DHX57**	NM_198963				*2.07*	0.0382	0.1719	0	
**TC1200007654**	**ATG101**	NM_001098673				*2.07*	0.0066	0.0522	0	
**TC1800009229**	**SMAD4**	NM_005359				*2.07*	0.0379	0.1714	1	MIRT284
**TC2000007717**	**MOCS3**	NM_014484				*2.07*	0.033	0.1565	1	
**TC0700009560**	**CNTNAP2**	NM_014141				*2.06*	0.0063	0.0507	1	
**TC0X00008760**	**ZNF185**	NM_001178106				*2.06*	0.017	0.0995	1	
**TC1700011774**	**TRIM47**	NM_033452				*2.06*	0.0338	0.1591	1	
**TC0200014907**	**SLC25A12**	NM_003705				*2.05*	0.0409	0.1789	1	
**TC0100017713**	**TTC13**	NM_001122835				*2.04*	0.0334	0.1576	1	
**TC0300013934**	**EMC3**	NM_018447				*2.04*	0.0349	0.1624	0	
**TC1100013178**	**MAP4K2**	NM_001307990				*2.04*	0.0056	0.0469	1	
**TC1700012472**	**MRPL38**	NM_032478				*2.04*	0.0301	0.1482	0	
**TC1400008455**	**ZBTB42**	NM_001137601				*2.03*	0.0102	0.0707	1	
**TC0400009935**	**CCDC96**	NM_153376				*2.03*	0.035	0.1628	1	
**TC1500009460**	**FAM214A**	NM_001286495				*2.02*	0.0353	0.1637	1	
**TC0100007845**	**MANEAL**	NM_001031740				*2.01*	0.0113	0.0756	1	MIRT68768
**TC0100017241**	**TMEM206**	NM_001198862				*2.01*	0.0291	0.1446	1	
**TC1700009376**	**MYO1C**	NM_001080779				*2*	0.0433	0.1854	1	

**TC0100007552**	**SESN2**	NM_031459				*1.99*	0.0161	0.0955	0	
**TC0100016903**	**TMEM9**	NM_001288564				*1.99*	0.0109	0.074	1	
**TC0800009201**	**GSDMD**	NM_024736				*1.99*	0.032	0.1537	1	
**TC0X00008844**	**IKBKG**	NM_001099856				*1.99*	0.027	0.1378	1	
**TC0X00010916**	**CT45A6; CT45A7**	NM_001017438				*1.98*	0.0385	0.1726	0	
**TC0100017028**	**PPP1R15B**	NM_032833				*1.97*	0.01	0.0696	1	MIRT28
**TC0900011496**	**PSMB7**	NM_002799				*1.97*	0.0372	0.1693	0	
**TC1100008085**	**PELI3**	NM_001098510				*1.97*	0.0364	0.167	1	
**TC0200016420**	**MRPL33**	NM_004891				*1.96*	0.0261	0.1348	0	
**TC0200015876**	**SERPINE2**	NM_001136528				*1.95*	0.0416	0.1807	1	
**TC0600009712**	**UTRN**	NM_007124				*1.95*	0.0109	0.0741	1	
**TC0100017568**	**JMJD4**	NM_001161465				*1.93*	0.0275	0.1393	1	
**TC0X00008259**	**NDUFA1**	NM_004541				*1.93*	0.0193	0.1086	0	
**TC1700007429**	**ANKRD13B**	NM_152345				*1.92*	0.0427	0.1838	1	
**TC0X00008811**	**TMEM187**	NM_003492				*1.9*	0.0164	0.0969	0	
**TC1200008322**	**TMTC2**	NM_152588				*1.89*	0.0341	0.1599	1	
**TC0600014260**	**ATP6V1G2**	NM_001204078				*1.89*	0.0148	0.0907	1	
**TC1900009668**	**SPC24**	NM_182513				*1.89*	0.0362	0.1663	1	
**TC1100009394**	**PANX3**	NM_052959				*1.88*	0.0356	0.1646	1	
**TC1500006967**	**GCHFR**	NM_005258				*1.87*	0.0191	0.1081	1	
**TC0700008928**	**CPED1**	NM_001105533				*1.86*	0.008	0.0597	1	
**TC0X00007574**	**ACRC**	NM_052957				*1.85*	0.0281	0.1412	0	
**TC1700012337**	**P2RX5**	NM_001204519				*1.84*	0.0409	0.1789	1	
**TC1600007307**	**C16orf82**	NM_001145545				*1.81*	0.0301	0.1481	1	
**TC1600008973**	**FBXL16**	NM_153350				*1.8*	0.0411	0.1796	1	
**TC1700012231**	**TMEM98**	NM_001033504				*1.8*	0.0396	0.1754	1	
**TC0700008389**	**ASB4**	NM_016116				*1.79*	0.0383	0.1723	1	
**TC1100012610**	**BSX**	NM_001098169				*1.79*	0.0318	0.1534	0	
**TC1600011472**	**NMRAL1**	NM_001305142				*1.79*	0.0178	0.1025	1	
**TC1900009669**	**KANK2**	NM_001136191				*1.78*	0.0384	0.1725	1	
**TC1700009113**	**GAA**	NM_000152				*1.77*	0.0318	0.1534	0	

**TC1200006645**	**CD4**	NM_000616				*1.75*	0.0317	0.1532	0	
**TC1900009269**	**TLE2**	NM_001144761				*1.75*	0.0333	0.1573	1	
**TC1100012444**	**PCSK7**	NM_004716				*1.73*	0.0353	0.1639	1	
**TC0100011859**	**IBA57**	NM_001010867				*1.73*	0.044	0.1874	1	MIRT48883
**TC1500010049**	**FAM219B**	NM_020447				*1.73*	0.0432	0.1852	1	
**TC0300011065**	**AMT; NICN1**	NM_000481				*1.72*	0.0424	0.183	0	
**TC0900012192**	**PHPT1**	NM_001135861				*1.71*	0.0361	0.1661	1	
**TC1000011716**	**USMG5; MIR1307**	NM_001206426				*1.71*	0.0422	0.1825	0	
**TC1700006679**	**RPAIN**	NM_001033002				*1.7*	0.0269	0.1373	0	
**TC0100007486**	**SFN**	NM_006142				*1.68*	0.0415	0.1803	1	
**TC1900010894**	**ZNF180**	NM_001278508				*1.67*	0.0368	0.1682	1	MIRT2827
**TC1000009327**	**MGMT**	NM_002412				*1.6*	0.032	0.1537	1	

Supplementary Table S4. miR-93 potential transcription factors with highest level of evidence (level 2) and for which the ChIP-seq data was derived from blood tissue (retrieved from TransmiR v2.0 database).

TF name	TSS	Binding site	Action type	SRAID/ PMID	Evidence	Tissue
**ATF3**	chr7:100 101379	Chr7: 100101352-100101481 (score=795)	Regulation	SRX100425	Level 2	Blood
**ATF3**	chr7:100 101379	Chr7: 100101356-100101490 (score=582)	Regulation	SRX100553	Level 2	Blood
**BHLHE40**	chr7:100 101379	Chr7: 100101308-100101550 (score=1000)	Regulation (feedback)	SRX150695	Level 2	Blood
**BRD4**	chr7:100 101379	Chr7: 100101446-100101561 (score=258)	Regulation (feedback)	SRX1460852	Level 2	Blood
**CBFB**	chr7:100 101379	Chr7: 100101386-100101604 (score=506)	Regulation	SRX265219	Level 2	Blood
**CHD2**	chr7:100 101379	Chr7: 100101405-100101529 (score=492)	Regulation (feedback)	SRX150581	Level 2	Blood
**CHD2**	chr7:100 101379	Chr7: 100101413-100101544 (score=283)	Regulation (feedback)	SRX150458	Level 2	Blood
**CREB1**	chr7:100 101379	Chr7: 100101386-100101570 (score=465)	Regulation (feedback)	SRX190216	Level 2	Blood
**E2F4**	chr7:100 101379	Chr7: 100101289-100101670 (score=1000)	Regulation	SRX150679	Level 2	Blood
**E2F4**	chr7:100 101379	Chr7: 100101328-100101642 (score=581)	Regulation	SRX150410	Level 2	Blood
**EGR1**	chr7:100 101379	Chr7: 100101490-100101661 (score=796)	Regulation (feedback)	SRX100459	Level 2	Blood
**ELF1**	chr7:100 101379	Chr7: 100101497-100101646 (score=333)	Regulation	SRX100539	Level 2	Blood
**ERG**	chr7:100 101379	Chr7: 100101351-100101632 (score=1000)	Regulation	SRX265230	Level 2	Blood
**ERG**	chr7:100 101379	Chr7: 100101360-100101629 (score=642)	Regulation	SRX682375	Level 2	Blood
**FOS**	chr7:100 101379	Chr7: 100101374-100101577 (score=1000)	Regulation	SRX015141	Level 2	Blood
**FOS**	chr7:100 101379	Chr7: 100101374-100101577 (score=1000)	Regulation	SRX150435	Level 2	Blood
**FOS**	chr7:100 101379	Chr7: 100101423-100101517 (score=384)	Regulation	SRX150489	Level 2	Blood
**GABPA**	chr7:100 101379	Chr7: 100101408-100101607 (score=725)	Regulation	SRX326887	Level 2	Blood
**GATA1**	chr7:100 101379	Chr7: 100101392-100101457 (score=316)	Regulation	SRX218418	Level 2	Blood
**IRF1**	chr7:100 101379	Chr7: 100101402-100101592 (score=529)	Regulation (feedback)	SRX150628	Level 2	Blood
**KDM5B**	chr7:100 101379	Chr7: 100101281-100101381 (score=258)	Regulation (feedback)	SRX186782	Level 2	Blood
**KLF1**	chr7:100 101379	Chr7: 100101385-100101463 (score=446)	Regulation	SRX218419	Level 2	Blood
**KMT2A**	chr7:100 101379	Chr7: 100101381-100101571 (score=468)	Regulation	SRX1293531	Level 2	Blood
**MAX**	chr7:100 101379	Chr7: 100101309-100101645 (score=1000)	Regulation	SRX150424	Level 2	Blood
**MAX**	chr7:100 101379	Chr7: 100101311-100101658 (score=1000)	Regulation	SRX150723	Level 2	Blood
**MAX**	chr7:100 101379	Chr7: 100101335-100101511 (score=788)	Regulation	SRX150618	Level 2	Blood
**MAX**	chr7:100 101379	Chr7: 100101345-100101623 (score=384)	Regulation	SRX129084	Level 2	Blood
**MAX**	chr7:100 101379	Chr7: 100101357-100101497 (score=485)	Regulation	SRX150597	Level 2	Blood
**MAX**	chr7:100 101379	Chr7: 100101366-100101523 (score=275)	Regulation	SRX204413	Level 2	Blood

**MAZ**	chr7:100 101379	Chr7: 100101484-100101667 (score=655)	Regulation (feedback)	SRX150363	Level 2	Blood
**MED1**	chr7:100 101379	Chr7: 100101433-100101611 (score=294)	Regulation	SRX204906	Level 2	Blood
**MYB**	chr7:100 101379	Chr7: 100101296-100101576 (score=796)	Regulation	SRX658611	Level 2	Blood
**MYB**	chr7:100 101379	Chr7: 100101319-100101546 (score=375)	Regulation	SRX725578	Level 2	Blood
**MYB**	chr7:100 101379	Chr7: 100101333-100101504 (score=341)	Regulation	SRX725586	Level 2	Blood
**MYB**	chr7:100 101379	Chr7: 100101333-100101506 (score=356)	Regulation	SRX658610	Level 2	Blood
**MYB**	chr7:100 101379	Chr7: 100101343-100101524 (score=355)	Regulation	SRX725584	Level 2	Blood
**MYC**	chr7:100 101379	Chr7: 100101295-100101663 (score=761)	Regulation (feedback)	SRX150627	Level 2	Blood
**MYC**	chr7:100 101379	Chr7: 100101301-100101493 (score=595)	Regulation (feedback)	SRX150722	Level 2	Blood
**NFE2**	chr7:100 101379	Chr7: 100101327-100101536 (score=1000)	Regulation	SRX1089830	Level 2	Blood
**NFE2**	chr7:100 101379	Chr7: 100101365-100101480 (score=1000)	Regulation	SRX218420	Level 2	Blood
**NFKB1**	chr7:100 101379	Chr7: 100101481-100101623 (score=630)	Regulation	SRX790970	Level 2	Blood
**NFYA**	chr7:100 101379	Chr7: 100101319-100101629 (score=1000)	Regulation	SRX037418	Level 2	Blood
**NFYA**	chr7:100 101379	Chr7: 100101361-100101579 (score=906)	Regulation	SRX150512	Level 2	Blood
**NFYB**	chr7:100 101379	Chr7: 100101302-100101640 (score=1000)	Regulation	SRX037419	Level 2	Blood
**NFYB**	chr7:100 101379	Chr7: 100101307-100101661 (score=1000)	Regulation	SRX150586	Level 2	Blood
**NFYB**	chr7:100 101379	Chr7: 100101308-100101616 (score=1000)	Regulation	SRX150508	Level 2	Blood
**PBX1**	chr7:100 101379	Chr7: 100101374-100101647 (score=1000)	Regulation (feedback)	SRX656349	Level 2	Blood
**PBX3**	chr7:100 101379	Chr7: 100101414-100101604 (score=1000)	Regulation (feedback)	SRX100577	Level 2	Blood
**RELA**	chr7:100 101379	Chr7: 100101448-100101579 (score=280)	Regulation	SRX1460848	Level 2	Blood
**RUNX1**	chr7:100 101379	Chr7: 100101403-100101661 (score=1000)	Regulation (feedback)	SRX1036365	Level 2	Blood
**RUNX1**	chr7:100 101379	Chr7: 100101403-100101661 (score=1000)	Regulation (feedback)	SRX669872	Level 2	Blood
**RUNX1**	chr7:100 101379	Chr7: 100101418-100101616 (score=519)	Regulation (feedback)	SRX063913	Level 2	Blood
**RUNX3**	chr7:100 101379	Chr7: 100101351-100101640 (score=415)	Regulation (feedback)	SRX190349	Level 2	Blood
**SP1**	chr7:100 101379	Chr7: 100101383-100101597 (score=902)	Regulation (feedback)	SRX100408	Level 2	Blood
**SP2**	chr7:100 101379	Chr7: 100101415-100101535 (score=544)	Regulation	SRX100447	Level 2	Blood
**STAT1**	chr7:100 101379	Chr7: 100101501-100101641 (score=290)	Regulation	SRX212648	Level 2	Blood
**USF1**	chr7:100 101379	Chr7: 100101293-100101564 (score=1000)	Regulation	SRX100392	Level 2	Blood
**USF1**	chr7:100 101379	Chr7: 100101363-100101492 (score=789)	Regulation	SRX1042049	Level 2	Blood
**USF2**	chr7:100 101379	Chr7: 100101316-100101533 (score=1000)	Regulation	SRX150637	Level 2	Blood
**USF2**	chr7:100 101379	Chr7: 100101338-100101490 (score=1000)	Regulation	SRX150436	Level 2	Blood
**USF2**	chr7:100 101379	Chr7: 100101356-100101500 (score=1000)	Regulation	SRX1042050	Level 2	Blood

Supplementary Table S5: panel of myeloid and lymphoid markers

**Myeloid cell staining**			
**mCD45**	BUV 395	30-F11	BD Biosciences
**mCD11b**	Brilliant Violet 750	M1/70	BioLegend
**mCD206**	FITC	MR5D3	BioLegend
**mF4/80**	PE-CY7	BM8	BioLegend
**mLy6C**	APC-CY7	HK1.4	BioLegend
**mLy6G**	PerCP	1A8	BioLegend
**mCSF1R**	PE	604B5 2E11	Bio-Rad
**mIA^b**	eFluor 450	AF6-120.1	eBioscience
**mPD-L1**	AlexaFluor 700	MIH5	Novus
**Lymphoid cell staining**			
**mCD62L**	BUV 395	MEL-14	BD Biosciences
**mCD4**	Brilliant Violet 650	GK1.5	BD Biosciences
**mCD19**	PE-CY7	1D3	BD Biosciences
**mCD3ε**	APC-CY7	145-2C11	BioLegend
**mCD8a**	PerCP	53-6.7	BioLegend
**mPD-1**	eFluor 450	RMP1-30	eBioscience
**mCD5**	APC	53-7.3	BD Biosciences
**mPD-L1**	AlexaFluor 700	MIH5	Novus
**mCD44**	BUV737	IM7	BD Biosciences
**mCD25**	Brilliant Violet 786	PC61	BD Biosciences
**mCD45**	Brilliant Violet 711	30-F11	BioLegend

Supplementary Table S6: Primer and sgRNA sequences and antibodies

qPCR Primer	Sequence Forward (5' to 3')	Sequence Reverse (5' to 3')
GAPDH	AGGTCGGAGTCAACGGATTT	ATGAAGGGGTCATTGATGGCA
BACTIN	GAGCACAGAGCCTCGCCTTT	TCATCATCCATGGTGAGCTGG
CD38	CCTGGGTGATACATGGTGGA	GATCCTGGCATAAGTCTCTGGA
IFNG	CTGTTACTGCCAGGACCCAT	TCCGCTACATCTGAATGACC
CD28	GTGGAGTCCTGGCTTGCTAT	GCTCCTCTTACTCCTCACCC
BCL6	AAGGCCAGTGAAGCAGAGAT	GAACTCTTCACGAGGAGGCT
CD160	TGGACATCCAGTCTGGTGGA	TTAGTCGCGTTCCTTCCTGG
STAT3	CCTTTGGAACGAAGGGTACA	GGCTTAGTGCTCAAGATGGC
ZRANB1	AATGCTTGTGTGGGGGTTG	TGACGTGCAATGTCTCCTCC
CD37	ATCCTCATCTCCACTCAGCG	GGTGCCGTACTTTTGGATGG
BTN3A1	CCTGGAGGAACTCAGATGGA	TGAATGTCTCTCTCCCCGAG
TGFBR2	TCATGTGTTCCTGTAGCTCTGA	CGCGGTAGCAGTAGAAGATG
RAP2C	CCGAGCAGATAAAACTCAGAGG	CCAAGGCTCAGTTCTGCAAC
MXD1	CGACTCCGACAGGGAAGAAA	AGATAGTCCGTGCTCTCCAC
CD6	GCGGTTCAACAACTCCAACC	ATTGTGCAAACTCCGGGAAG
SMAD4	AGAACATTGGATGGGAGGCT	CCAGAGACGGGCATAGATCA
TGFBR1	CCAAACCACAGAGTGGGAAC	CGTCGAGCAATTTCCCAGAA
RNF216	CAAAGAGATGGCAGAGCATGAA	ATAGCAGCAGCGACACTCA
TRAF6	CCCCAATTCCATGCACATTCA	TGTGTGACTGGGTGTTCTCT
SUSD6	AAGAGCACCTCAGTGTTTGC	GGGGCACACGGAAGCA
TANK	ACAGCAAAAGACTGAGAACTATGA	GCTGTTCCTGTTGTTCACGTA
PAK1	TCATGTCGGTTTTGATGCTGT	GCTCTGGCATTCCCGTAAAC
IP6K1	TCCAAGGACCGAAAGCTCTA	CCATCTTCAGGTCCAACACG
AJUBA	ATGCTGTGTCTGTGGTCACT	AGGACTTCCCCATTGCTTGTA
SOS1	CAGTTATCAAAGCCTGGGGC	CCTTCGCCTATTGACTGCAA
SLC2A4	CCCCGCTACCTCTACATCATC	CAGGCGCTTCAGACTCTTTC
IFNGR1	AGCCAGGGTTGGACAAAAAGA	ACTTCCTGCTCGTCTCCATTTA
TGFB	GACATCAACGGGTTCACTACC	AATGTACAGCTGCCGCACG
MAP2K7	GCAAGATGACAGTGGCGATT	CCGTGCTTCTCCTTCAGGTA
CCL28	ACTTGGCTGCTGTCATCCTT	TAACAGTATGGTTGTGCGGG
CXCR4	CTTCCTGCCCACCATCTACTC	ATGACCAGGATGACCAATCCA
IKBKG	GCTGCCTGGAGGAGAATCAA	CAGAATCTGGTTGCTCTGCC
DUSP16	TGCCGTGGTTGGACAAATC	AGATCCCAGCTAAACAGTGC
PELI3	CAAGCTGGTGGAAAACGAGT	CCCACACAGGTCGATGAGAG
MAL3K3	GAAGCTGCCAATCCTTGGAC	GCACGGGACATTCGTGATTT
TPK1	CTACCAGCCATTGTAGGCCA	AATTCCCAGTGGAAAGCAGG
ATF1	ATCAGACTAGCAGCGGACAG	TGCCAACTGTAAGGCTCCAT

ZAP70	GAGTGTCCACCCGAACTGTA	GCGATCCTCCCACTTGTAGA
MAP4K2	CTGAGGACTGTGAGCTGGAG	CCGGGAGTGAATGGTGTCT
PFKP	GAGCACCTGACGGAGAAAAT	TCTCATTTCTGAGCACAAGGC
IFNGR2	CAATGTCACTCTACGCCTTCG	TGATGAGGGAGCCTTCTCCT
CD99	GAAAATGACGACCCACGACC	GGTTTGGATTTGGCATCGGT
CD83	GCCGACAAGAGCAGTTTCAT	AGACATAGGCCCCCATCCTA
STAT1	GCCAAAGGAAGCACCAGAG	GAGCAGGTTGTCTGTGGTCT
IL4R	AGCTCTGGGAACATGAAGGT	CCTCCGTTGTTCTCAGGGATA
**DNA Primer**	**Sequence Forward (5' to 3')**	**Sequence Reverse (5' to 3')**
miR-93 locus	ACCTTCACTGAGAGGGTGGT	AGACCCTTTTGAACGCCACT
**sgRNA**	**Sequence upstream (5’ to 3’)**	**Sequence downstream (5’ to 3’)**
miR-93 KO	CTCCAAAGTGCTGTTCGTGC(AGG)	GTGCTAGCTCAGCAGTAGGT(TGG)
**Antibody**	**Catalog number**	**Company**
MCM7 mAb (D10A11)	3735S	Cell Signaling
GAPDH mAb (0411)	sc-47724	Santa Cruz
AGO2 mAb (2E12-1C9)	H00027161-M01	Abnova
IgGκ mAb (MG1-45)	Ab18447	Abcam
STAT1 mAb (D1K9Y)	14994	Cell Signaling
Anti-F4/80 mAb [CI:A3-1]	ab6640	Abcam
Goat Anti-Rat IgG H&L (Alexa Fluor® 647) pAb	ab150159	Abcam

References

1. Rittirsch D, et al. Immunodesign of experimental sepsis by cecal ligation and puncture. *Nat Protoc.* 2009;4(1):31-6.
2. Pichler M, et al. Therapeutic potential of FLANC, a novel primate-specific long non-coding RNA in colorectal cancer. *Gut.* 2020.
3. Silasi R, et al. Inhibition of contact-mediated activation of factor XI protects baboons against S aureus-induced organ damage and death. *Blood Adv.* 2019;3(4):658-69.
4. Keshari RS, et al. Inhibition of complement C5 protects against organ failure and reduces mortality in a baboon model of Escherichia coli sepsis. *Proc Natl Acad Sci U S A.* 2017;114(31):E6390-E9.
5. Silasi R, et al. Factor XII plays a pathogenic role in organ failure and death in baboons challenged with Staphylococcus aureus. *Blood.* 2021;138(2):178-89.
6. Keshari RS, et al. Complement C5 inhibition protects against hemolytic anemia and acute kidney injury in anthrax peptidoglycan-induced sepsis in baboons. *Proc Natl Acad Sci U S A.* 2021;118(37).
7. Popescu NI, et al. C3 Opsonization of Anthrax Bacterium and Peptidoglycan Supports Recognition and Activation of Neutrophils. *Microorganisms.* 2020;8(7).
8. Popescu NI, et al. Peptidoglycan induces disseminated intravascular coagulation in baboons through activation of both coagulation pathways. *Blood.* 2018;132(8):849-60.
9. Ginsburg I. Role of lipoteichoic acid in infection and inflammation. *Lancet Infect Dis.* 2002;2(3):171-9.
10. Hinshaw LB, et al. Lethal Staphylococcus aureus-induced shock in primates: prevention of death with anti-TNF antibody. *J Trauma.* 1992;33(4):568-73.
11. Knaus WA, et al. APACHE II: a severity of disease classification system. *Crit Care Med.* 1985;13(10):818-29.
12. Vincent JL, et al. The SOFA (Sepsis-related Organ Failure Assessment) score to describe organ dysfunction/failure. On behalf of the Working Group on Sepsis-Related Problems of the European Society of Intensive Care Medicine. *Intensive Care Med.* 1996;22(7):707-10.
13. Shapiro NI, et al. Mortality in Emergency Department Sepsis (MEDS) score: a prospectively derived and validated clinical prediction rule. *Crit Care Med.* 2003;31(3):670-5.
14. Yang Z, et al. Cardiac Troponin Is a Predictor of Septic Shock Mortality in Cancer Patients in an Emergency Department: A Retrospective Cohort Study. *PLoS One.* 2016;11(4):e0153492.
15. Basar R, et al. Large-scale GMP-compliant CRISPR-Cas9-mediated deletion of the glucocorticoid receptor in multivirus-specific T cells. *Blood Adv.* 2020;4(14):3357-67.
16. Dragomir MP, et al. The non-coding RNome after splenectomy. *J Cell Mol Med.* 2019;23(11):7844-58.
17. Vandesompele J, et al. Accurate normalization of real-time quantitative RT-PCR data by geometric averaging of multiple internal control genes. *Genome Biol.* 2002;3(7):RESEARCH0034.
18. Kishimoto C, et al. Enhanced production of macrophage inflammatory protein 2 (MIP-2) by in vitro and in vivo infections with encephalomyocarditis virus and modulation of myocarditis with an antibody against MIP-2. *J Virol.* 2001;75(3):1294-300.

19. Aziz M, et al. Milk fat globule-epidermal growth factor-factor 8 attenuates neutrophil infiltration in acute lung injury via modulation of CXCR2. *J Immunol.* 2012;189(1):393-402.
20. Yu C, et al. Rhein prevents endotoxin-induced acute kidney injury by inhibiting NF-kappaB activities. *Sci Rep.* 2015;5:11822.
21. Martin EL, et al. Phosphoinositide-3 kinase gamma activity contributes to sepsis and organ damage by altering neutrophil recruitment. *Am J Respir Crit Care Med.* 2010;182(6):762-73.
22. Karamese M, et al. Anti-oxidant and anti-inflammatory effects of apigenin in a rat model of sepsis: an immunological, biochemical, and histopathological study. *Immunopharmacol Immunotoxicol.* 2016;38(3):228-37.
23. Rosenlund IA, et al. CRISPR/Cas9 to Silence Long Non-Coding RNAs. *Methods Mol Biol.* 2021;2348:175-87.
24. Tan LP, et al. A high throughput experimental approach to identify miRNA targets in human cells. *Nucleic Acids Res.* 2009;37(20):e137.
25. Clarke DJB, et al. Appyters: Turning Jupyter Notebooks into data-driven web apps. *Patterns (N Y).* 2021;2(3):100213.
26. Benjamini Y. Discovering the false discovery rate. *Journal of the Royal Statistical Society: Series B (Statistical Methodology).* 2010;72(4):405-16.
27. Gałecki A, and Burzykowski T. *Linear mixed-effects model. InLinear mixed-effects models using R.*: Springer, New York, NY.; 2013.

Supplementary Figure 13D

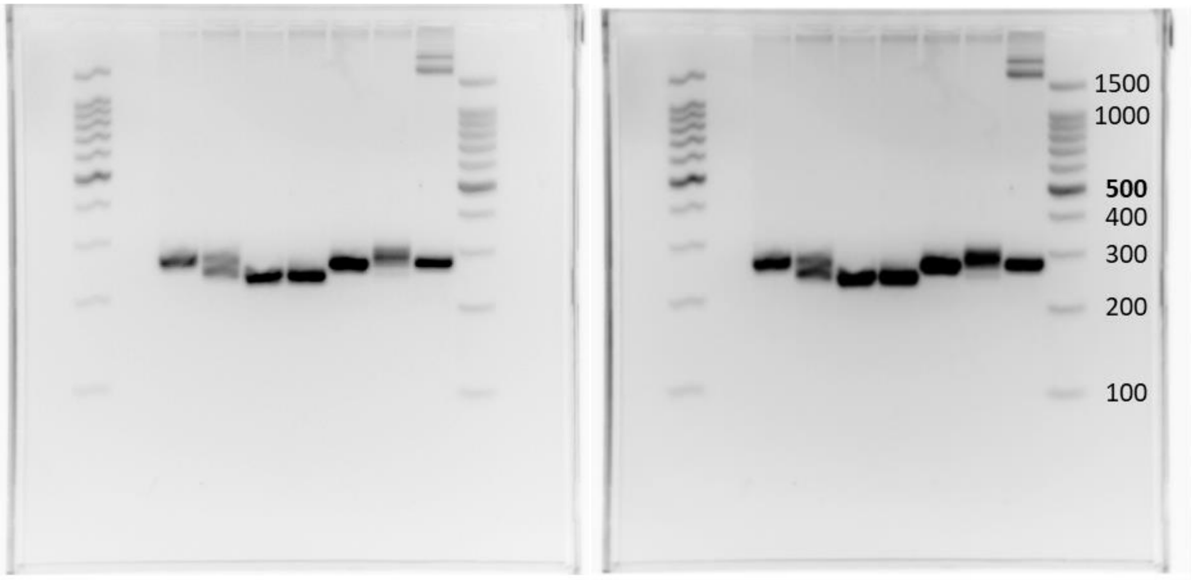

PCR product gel electrophoresis, 3% agarose gel, 100V, 5h, 100bp ladder (Promega).

<u>Samples left to right</u>: ladder, (empty), clone#2 (miR-93+/-), clone#4 (miR-93-/-), clone #6 (miR-93-/-), clone #11 (miR-93 +/+), clone #13 (miR-93+/-), clone #23 (miR-93+/+), ladder.

Supplementary Figure 13G

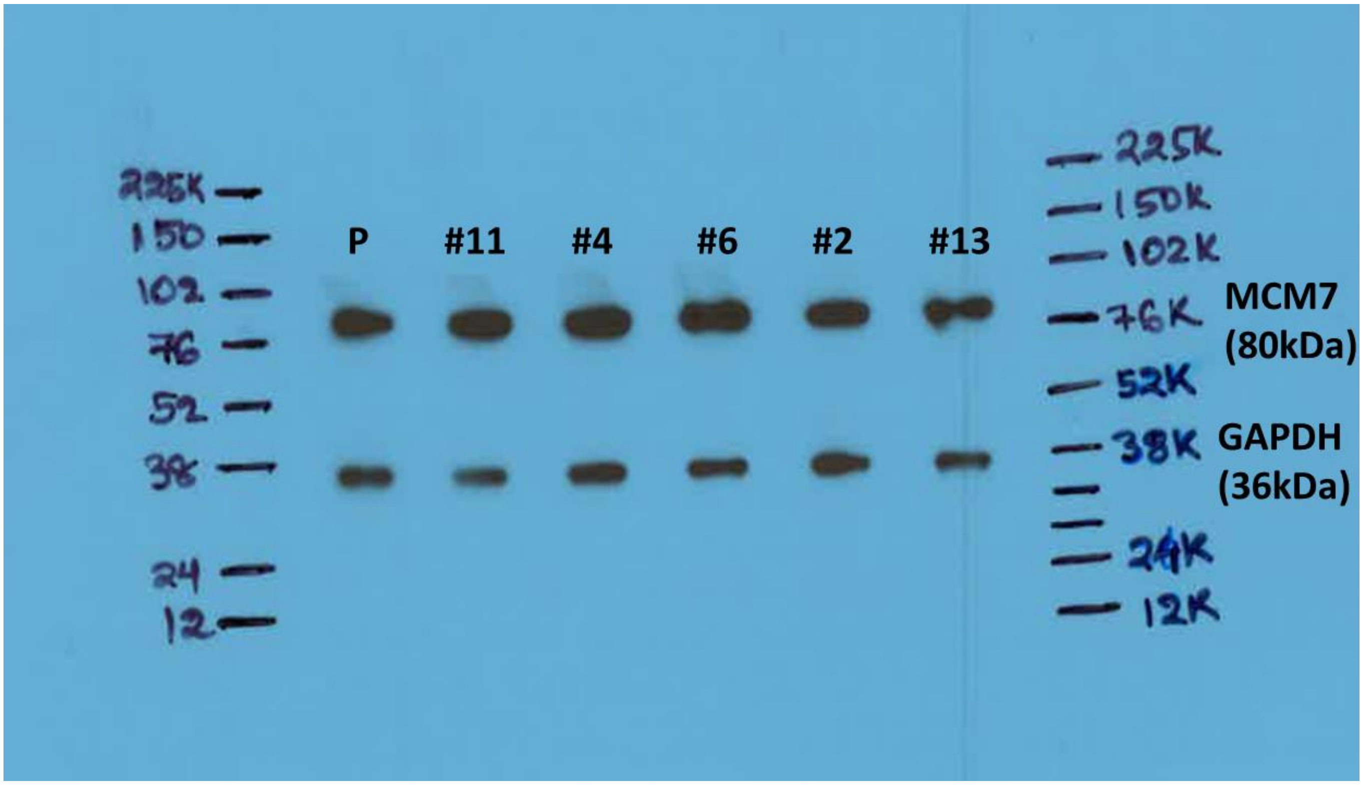

Western Blot, Amersham ECL Rainbow Marker Full Range (Sigma).

<u>Samples left to right</u>: marker, (empty), Jurkat parental, (empty), clone#11 (miR-93 +/+), (empty), clone#4 (miR-93-/-), (empty), clone #6 (miR-93-/-), (empty), clone#2 (miR-93+/-), (empty), clone #13 (miR-93+/-), (empty), marker.

Supplementary Figure 14A

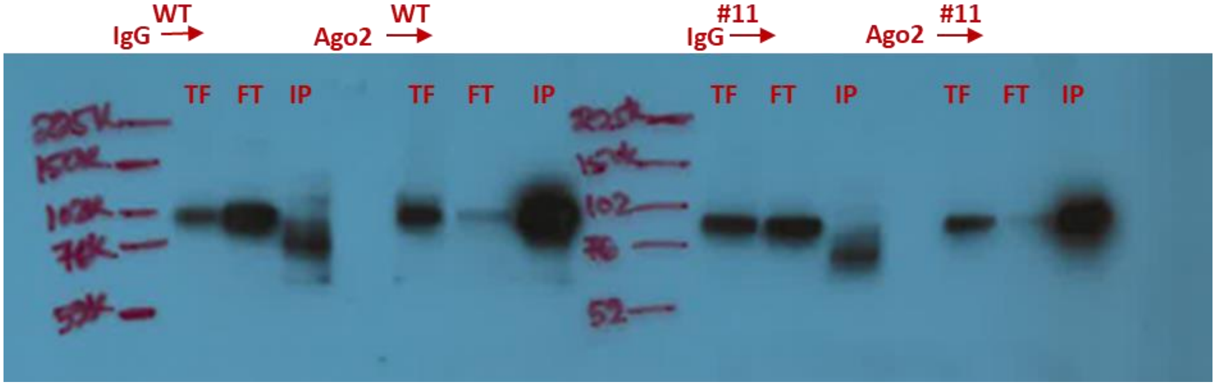

Western Blot, Amersham ECL Rainbow Marker Full Range (Sigma).

<u>Samples left to right</u>: marker, Jurkat parental total fraction (TF) IgG-IP, Jurkat parental flow through (FT) IgG-IP, Jurkat parental IP fraction IgG-IP, (empty), Jurkat parental total fraction (TF) Ago2-IP, Jurkat parental flow through (FT) Ago2-IP, Jurkat parental IP fraction Ago2-IP, (empty), marker, clone#11 (miR-93 +/+) total fraction (TF) IgG-IP, clone#11 (miR-93 +/+) flow through (FT) IgG-IP, clone#11 (miR-93 +/+) IP fraction IgG-IP, (empty), clone#11 (miR-93 +/+) total fraction (TF) Ago2-IP, clone#11 (miR-93 +/+) flow through (FT) Ago2-IP, clone#11 (miR-93 +/+) IP fraction Ago2-IP.

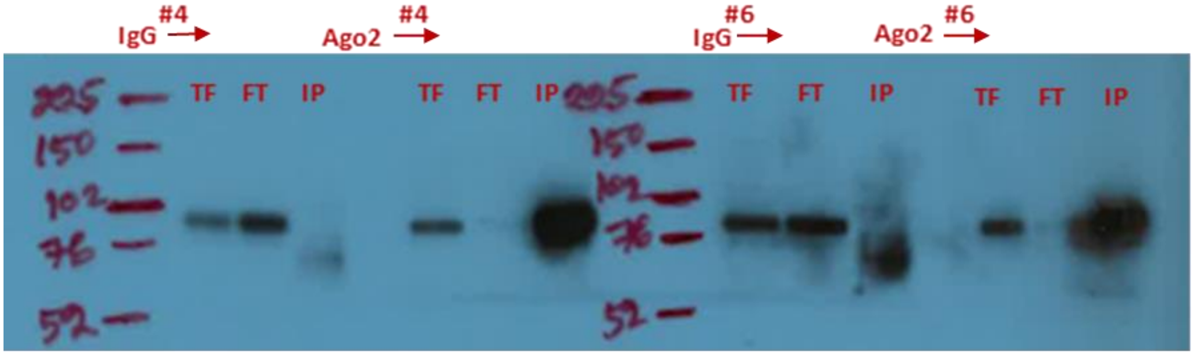

<u>Samples left to right</u>: marker,), clone#4 (miR-93-/-) total fraction (TF) IgG-IP,), clone#4 (miR-93-/-) flow through (FT) IgG-IP,), clone#4 (miR-93-/-) IP fraction IgG-IP, (empty),), clone#4 (miR-93-/-) total fraction (TF) Ago2-IP,), clone#4 (miR-93-/-) flow through (FT) Ago2-IP,), clone#4 (miR-93-/-) IP fraction Ago2-IP, (empty), marker, clone #6 (miR-93-/-) total fraction (TF) IgG-IP, clone #6 (miR-93-/-) flow through (FT) IgG-IP, clone #6 (miR-93-/-) IP fraction IgG-IP, (empty), clone #6 (miR-93-/-) total fraction (TF) Ago2-IP, clone #6 (miR-93-/-) flow through (FT) Ago2-IP, clone #6 (miR-93-/-) IP fraction Ago2-IP.

NB: the anti-mouse secondary HRP-linked antibody used also reacts with the murine normal IgG used as a control antibody for the immunoprecipitation. The band visible in the IgG-mediated IP fraction is thus the IgG heavy chain (ca. 68KDa). The band for specific for Ago2 is visible at ca. 95kDa.

Figure 5D

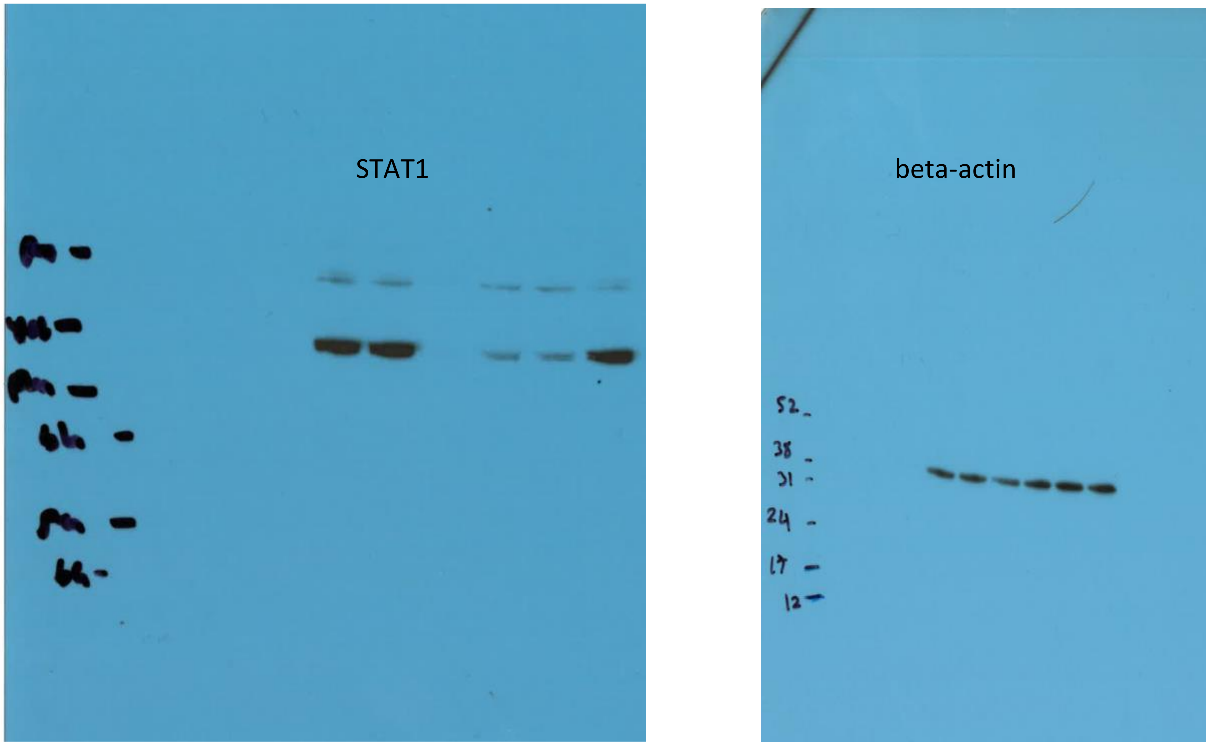

Western Blot, Amersham ECL Rainbow Marker Full Range (Sigma).

Samples left to right (for both gels): Jurkat Parental, Jurkat shControl, Jurkat shSTAT1-A, Jurkat shSTAT1-B, Jurkat shSTAT1-C, and Jurkat shSTAT1-D.

Supplementary Figure 16B

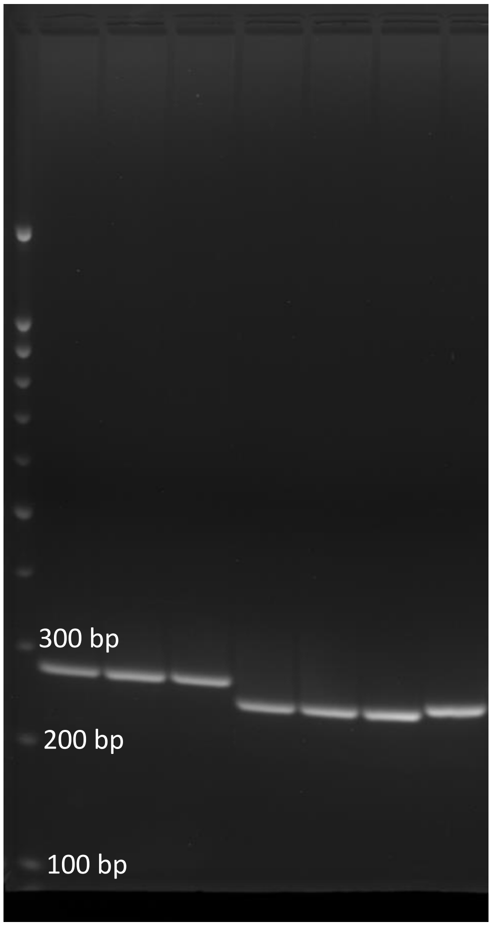

PCR product gel electrophoresis, 3% agarose gel, 100V, 5h, 100bp ladder (Promega).

Samples left to right: ladder, NB4 parental, 1-G4 (miR-93+/+), 2-F2 (miR-93+/+), 2-F10 (miR-93-/-), 2-E6 (miR-93-/-), 2-D7 (miR-93-/-), 1-D5 (miR-93-/-, with slightly different deletion size).

Supplementary Figure 16E

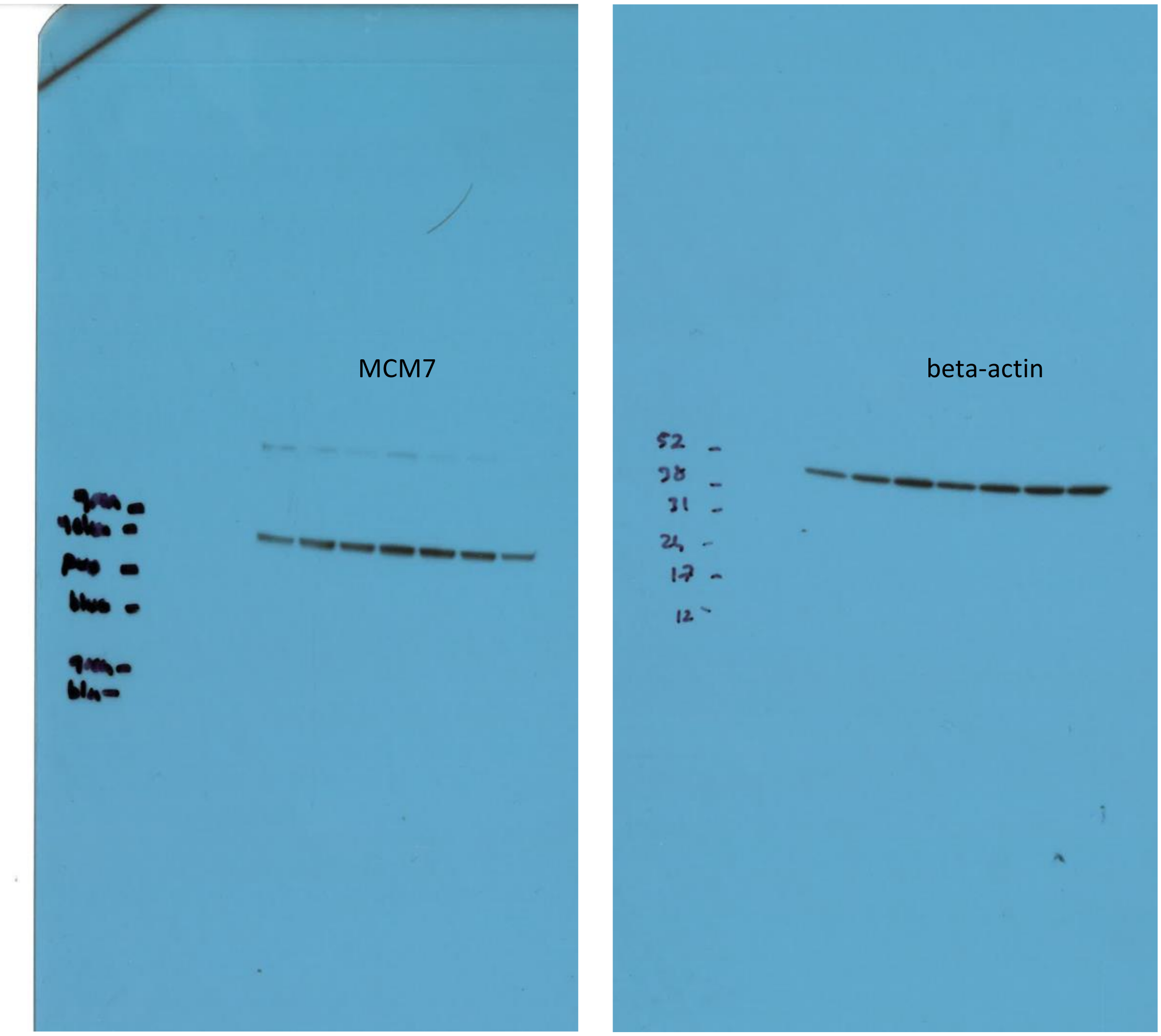

Western Blot, Amersham ECL Rainbow Marker Full Range (Sigma).

<u>Samples left to right (for both gels)</u>: marker, (empty), NB4 parental, NB4 1-G4 (miR-93+/+), NB4 2-F2 (miR-93+/+), NB4 2-F10 (miR-93-/-), NB4 2-E6 (miR93-/-), NB4 2-D7 (miR93-/-), NB4 1-D5 (miR93-/-).
